# Synthetic Nuances to Maximize n-Type Organic Electrochemical Transistor and Thermoelectric Performance in Fused Lactam Polymers

**DOI:** 10.1021/jacs.2c00735

**Published:** 2022-03-08

**Authors:** Adam Marks, Xingxing Chen, Ruiheng Wu, Reem B. Rashid, Wenlong Jin, Bryan D. Paulsen, Maximilian Moser, Xudong Ji, Sophie Griggs, Dilara Meli, Xiaocui Wu, Helen Bristow, Joseph Strzalka, Nicola Gasparini, Giovanni Costantini, Simone Fabiano, Jonathan Rivnay, Iain McCulloch

**Affiliations:** † Department of Chemistry, University of Oxford, Oxford OX1 3TA, U.K.; ‡ KAUST Solar Center (KSC), King Abdullah University of Science and Technology (KAUST), Thuwal 23955-6900, Saudi Arabia; § Department of Chemistry, Northwestern University, Evanston, Illinois 60208, United States; ∥ Department of Biomedical Engineering, Northwestern University, Evanston, Illinois 60208, United States; ⊥ Laboratory of Organic Electronics, Department of Science and Technology, Linköping University, NorrköpingSE-60174, Sweden; # Department of Material Science, Northwestern University, Evanston, Illinois 60208, United States; ∇ Department of Chemistry, University of Warwick, Coventry CV4 7AL, U.K.; ○ X-ray Science Division, Argonne National Laboratory, Lemont, Illinois 60439, United States; ◆ Department of Chemistry and Centre for Processable Electronics, Imperial College London, London W12 0BZ, U.K.

## Abstract

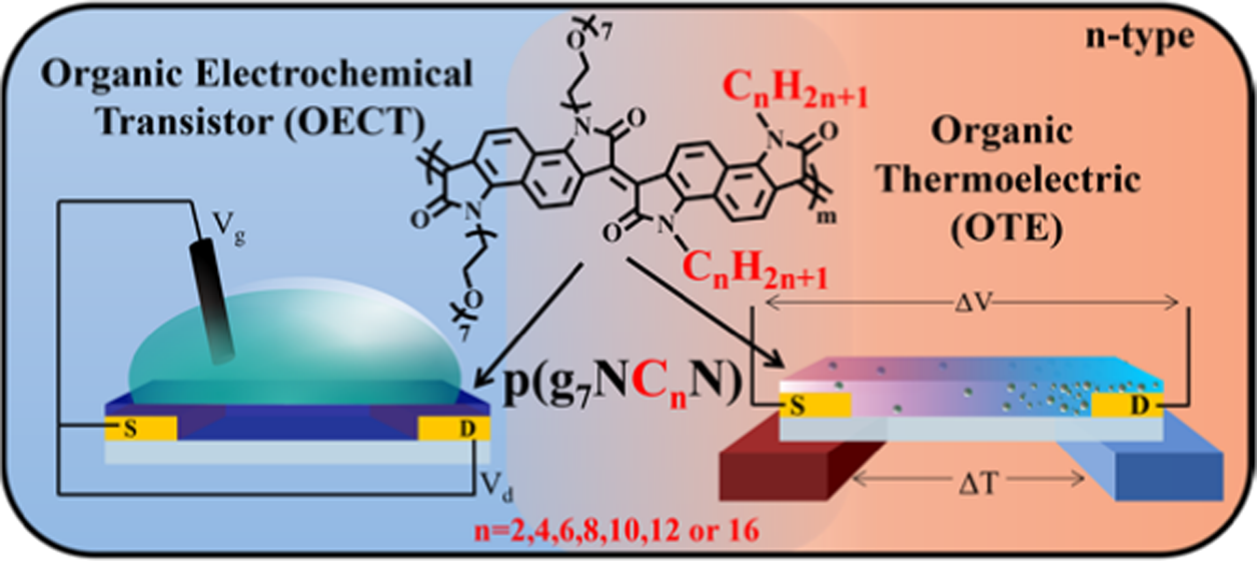

A series of fully fused n-type mixed conduction lactam polymers **p(g**_**7**_**NC***_**n**_***N)**, systematically increasing the alkyl side chain content, are synthesized via an inexpensive, nontoxic, precious-metal-free aldol polycondensation. Employing these polymers as channel materials in organic electrochemical transistors (OECTs) affords state-of-the-art n-type performance with **p(g**_**7**_**NC**_**10**_**N)** recording an OECT electron mobility of 1.20 × 10^–2^ cm^2^ V^–1^ s^–1^ and a μC* figure of merit of 1.83 F cm^–1^ V^–1^ s^–1^. In parallel to high OECT performance, upon solution doping with (4-(1,3-dimethyl-2,3-dihydro-1*H*-benzoimidazol-2-yl)phenyl)dimethylamine (N-DMBI), the highest thermoelectric performance is observed for **p(g**_**7**_**NC**_**4**_**N)**, with a maximum electrical conductivity of 7.67 S cm^–1^ and a power factor of 10.4 μW m^–1^ K^–2^. These results are among the highest reported for n-type polymers. Importantly, while this series of fused polylactam organic mixed ionic–electronic conductors (OMIECs) highlights that synthetic molecular design strategies to bolster OECT performance can be translated to also achieve high organic thermoelectric (OTE) performance, a nuanced synthetic approach must be used to optimize performance. Herein, we outline the performance metrics and provide new insights into the molecular design guidelines for the next generation of high-performance n-type materials for mixed conduction applications, presenting for the first time the results of a single polymer series within both OECT and OTE applications.

## Introduction

**Figure 1:**
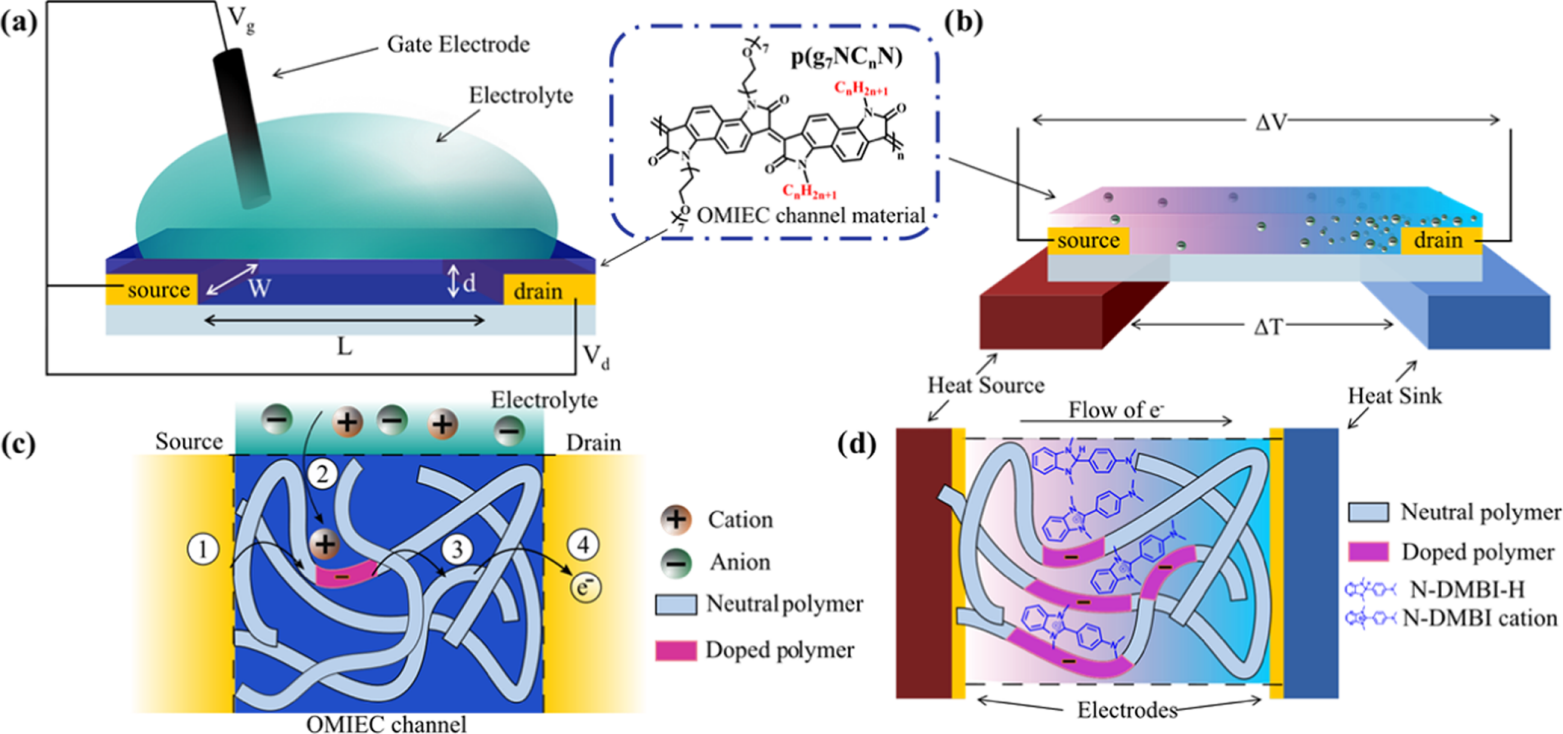
(a) Schematic diagram of an OECT device. (b) Schematic architecture of an n-type OTE generator leg, where the Seebeck coefficient is determined by measuring the thermovoltage (Δ*V*) across a thermal gradient (Δ*T*). (c) Illustration of electrochemical n-type OMIEC doping within an OECT, (1) electron injection from the source electrode, (2) electron stabilized by a dopant cation, (3) charge carrier hopping and transport, and (4) transfer of charge from the OMIEC to the drain electrode. (d) Schematic illustration of N-DMBI-doped **p(g**_**7**_**NC***_**n**_***N)** films. N-DMBI cations dope portions of the n-type OMIEC, and increased dopant concentration disturbs polymer microstructure and morphology.^27,32,38^

Organic mixed ionic–electronic conductors (OMIECs) have garnered increasing interest over the past few years due to their outstanding biocompatibility, mechanical flexibility, and operability in aqueous environments and have been employed in multiple bioelectronic devices.^1−7^ One such device is the organic electrochemical transistor (OECT), consisting of a conducting channel layer, generally an OMIEC, which resides between a source and drain electrode and is in direct contact with an aqueous electrolyte (Figure 1a). A third gate electrode is immersed in the electrolyte, and upon application of a gate voltage (*V*_G_), the OMIEC, commonly a conjugated polymer, becomes doped and ions penetrate from the electrolyte into the bulk of the transistor channel to compensate for the injected charge. Subsequently, the ionic signals can be transduced into electronic ones throughout the channel and charge carriers are extracted at the drain electrode.^8^ The transduction efficiency is quantified by the OECT transconductance *g*_m_ = μC* (*Wd*/*L)* (*V*_th_ – *V*_G_) where *W* is the width, *d* is the thickness, and *L* is the length of the OMIEC channel layer; μ is the charge carrier mobility; C* is the volumetric capacitance; *V*_th_ is the threshold voltage; and *V*_G_ is the gate voltage. As such, the transconductance is dependent upon the properties of the OMIEC, specifically, charge carrier mobility and ion miscibility.^9^ However, *g*_m_ also depends on the channel geometry and biasing conditions and describes the steady-state device performance. As a result, it does not solely represent the performance of the OMIEC, and thus the product of μ and C* has been postulated as a more appropriate figure of merit for comparing OMIEC performance within OECT devices.^10^ Indeed, the μC* product  is the material-dependent factor, which over the last 5 years has become the standard figure of merit for reporting the OECT performance between different channel materials.^10^ Hence, to improve OECT device performance, an in-depth understanding of the structure–property relationships governing OMIEC performance is crucial for both the mobility and volumetric capacitance of the OMIEC to be optimized.^11^

Currently, most reported OECT channels are composed of commercially available poly(ethylenedioxythiophene):poly(styrene sulfonate) (PEDOT:PSS) derivatives.^12^ However, the inclusion of PSS causes PEDOT to be intrinsically doped and operate in depletion mode, resulting in limited operational stability and increased power consumption.^13^ As such, recent studies have highlighted the benefits of ethylene glycol (EG) functionalized semiconducting conjugated polymers as channel materials for OECTs with the majority of these materials operating in accumulation (enhancement) mode.^14−16^ Additional benefits include: impressive OECT device performance (rivaling PEDOT derivatives) by improving C*,^10,17−19^ facile synthetic tunability,^1,17,20^ and heightened enzymatic biocompatibility allowing for direct detection of biologically pertinent metabolites.^2,21^ However, to date, the vast majority of all documented OECTs have relied on hole transport (p-type) materials, which exhibit higher performance than their electron-transporting (n-type) counterparts, an area of research that has only recently started to be explored.^22−24^ Empirical and theoretical evidence has shown that this developmental delay is mainly due to the ambient operational instability of electron-transporting n-type materials (in their doped states) and not due to differing intrinsic transport mechanisms for holes and electrons.^25−27^ This ambient instability generally arises from the vulnerability of n-type materials to react with atmospheric water and or oxygen, particularly when their electron affinity (EA) is less than ∼4.0 eV.^27−29^ Arguably, the development of high-performing electron transport materials should be prioritized over p-type materials, to increase the abundance of materials for selection in complementary logic circuits, which lower power consumption, show increased switching speeds and enhanced operational stability, and can only be realized with comparable p- and n-type material performance.^23,30,31^

Akin to the previously discussed OECT devices, OMIECs have also started to be incorporated into thermoelectric devices.^32^ Organic thermoelectric (OTE) generators convert thermal energy into power by applying a thermal gradient across organic semiconductor (OSC) layers. This thermal gradient causes charge carriers to diffuse away from the heated side of the OTE material (Figure 1b), generating a potential difference across the OMIEC channel, known as a thermovoltage, which is measured as the Seebeck coefficient (*S*), the ratio of voltage difference to temperature difference across the material.^33^ The performance of thermoelectric devices can be compared using the figure of merit *ZT* = PF/κ*T* where *T* is the temperature and *Z* combines the power factor (PF = *S*^2^σ), composed of the Seebeck coefficient, electrical conductivity (σ), and thermal conductivity (κ).^34^ This dimensionless figure of merit applies to both n- and p-type materials and highlights the importance of developing improved n-type materials, as the most efficient thermoelectric generators are composed of p- and n-type materials with similar ZT values.^35,36^ Practically, measuring the thermal conductivity of OSC films is limited and challenging. Instead, the performance of materials is often compared using PF.^27^ Similarly to OECT devices, the current library of reported n-type OTE materials lags behind their p-type counterparts, and is further constrained by the limited availability of n-type dopants, impeding the evolution of practical OTE applications.^37^ As such, developing n-type polymers with improved thermoelectric performance and formulating structure–property relationships are paramount to the growth of the field.

Notably, both OECT and OTE devices rely on doping to generate free mobile charges: the OMIEC can be doped either electrochemically (Figure 1c) or chemically (Figure 1d) for OECTs and OTEs, respectively.^38,39^ Thus, it is possible that OMIEC polymers could potentially be well matched for both applications, offering mixed conduction materials, which can be readily doped. However, key differences between dopant properties and the dynamic nature of the processes must be considered. In an OECT device, dopant ions are mobile and operate in a dynamic regime between the conductive polymer and the electrolyte.^12^ Conversely, chemical doping within OTEs is a more static process, whereby following solution chemical doping, for example, with N-DMBI, the doped film is cast and changes in polymer microstructure and morphology, compared to the neat material, are then effectively unchanged upon OTE operation.^40^ Similar levels of disruption can be observed within OECTs owing to the inclusion of a hydration shell around the dopant ions leading to polymer swelling, observed during operation.^41,42^ Despite this, OMIEC materials offer a promising future for both applications and warrant further exploration in OTE devices.^38^ As the acronym implies, OMIEC materials were specifically designed to operate within OECT devices, as the intrinsic ability to conduct both electronically and ionically is essential for OECT device operation.

Generally, OMIEC materials have been decorated with EG side chains to facilitate conduction and diffusion of aqueous ionic species, a design strategy that has also recently been deemed beneficial for OTE performance.^43−45^ This is evidenced through increased doping efficiency and electrical conductivity, which can be explained by the areas of hydrophilicity effectively confining dopant molecules within the polymers’ side chains, thereby minimizing disruption to the polymers’ π–π packing regions, which are necessary for efficient intermolecular electronic charge carrier transport.^43,44^ More broadly, these results would suggest that OMIEC materials are also well suited to OTE applications; however, the comparative performance of a single series of OMIEC polymers within both OECT and OTE devices has not been reported.^27^ Indeed, the two applications seem to be more intertwined than the common historical comparison between OECTs and organic thin-film transistors (OTFTs). As the field of bioelectronics and OECTs has grown, a common chemical design strategy has been to use high-performing OTFT materials and synthetically modify the material to enable ionic transport, commonly via the substitution of aliphatic side chains with EG side chains.^15,46^ However, this has often proven to be unsuccessful requiring vastly different side-chain compositions alongside alternative synthetic and processing techniques to be employed to develop high-performance OECT materials.^15,46−49^ In a homologous manner, initial research into n-type OTE materials began with simply doping traditional n-type polymers previously reported in OTFTs, although again this was relatively unsuccessful, with insufficient doping and unstable operational electron transport hindering performance.^37^

The current library of OECT channel materials is heavily populated with donor–acceptor (D–A) polymers, with the vast majority of these polymers containing at least one EG functionalized repeat unit.^12,27^ Typically, EG-functionalized OMIECs are synthesized via palladium-catalyzed Stille cross-coupling polymerization, involving the use of highly toxic stannylated monomers.^15,18,49,50^ Residual metallic species, such as toxic organostannanes and expensive transition-metal catalysts, can be difficult to completely remove post-polymerization and pose a further limitation toward bioelectronic applications.^51^ To combat these shortcomings, alternative polymerization strategies have been developed to yield acceptor–acceptor (A–A) polymers via a benign aldol polycondensation.^52^ Moreover, the presence of C=C double bonds linking successive aromatic cores in fused polylactam polymers minimizes torsional twists along the conjugated backbone, aiding lowest unoccupied molecular orbital (LUMO) frontier orbital delocalization across the entire polymer backbone and therefore electron mobility.^53^ In a recent report, we developed **PgNaN**, a polylactam-based polymer that showed promising OECT performance, incurring a μC* value of 0.66 ± 0.11 F cm^–1^ V^–1^ s^–1^. Although this was since superseded by an NDI derivative, **PgNaN**-based devices outperformed the majority of other n-type OECT materials.^27,49,54^

**Figure 2:**
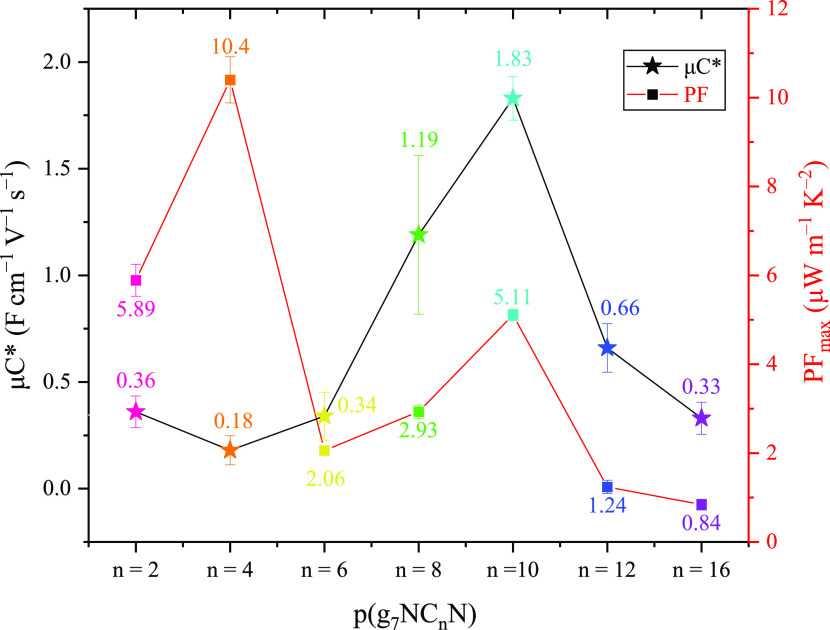
OECT (μC*) and OTE (PF_max_) figure of merit comparison and trend for the **p(g**_**7**_**NC***_**n**_***N)** series.

Herein, we set out to investigate whether the design criteria to optimize OECT performance of a series of fully fused acceptor–acceptor polylactams (**p(g**_**7**_**NC***_**n**_***N)**) translates linearly to OTE performance, producing the first cumulative report that uses the same series of materials within both applications. The impressive figure of merit device performances demonstrate that high performance can be achieved within both OECT and OTE devices; however, specific synthetic design nuances (specifically the proportion of hydrophilic to hydrophobic side chains) are required to optimize the performance metrics in both applications. The highest OECT performance is observed for **p(g**_**7**_**NC**_**10**_**N)**, recording an impressive OECT electron mobility of (1.20 ± 0.07) × 10^–2^ cm^2^ V^–1^ s^–1^ and a maximum μC* figure of merit value of 1.83 F cm^–1^ V^–1^ s^–1^. On the contrary, the highest OTE performance was recorded for **p(g**_**7**_**NC**_**4**_**N)** with a maximum electrical conductivity of 7.67 S cm^–1^ and a power factor of 10.4 μW m^–1^ K^–2^ (Figure 2).

These results highlight that the shared mixed conduction and doping requirements within the field of OECTs and OTEs allow for a broad chemical design strategy to achieve generally high performance. This constitutes a paradigm shift from the historic approach of attempting to convert high-performing OTFT materials into high-performing OECT or OTE materials through major synthetic design overhauls.

## Results and Discussion

**Figure 3:**
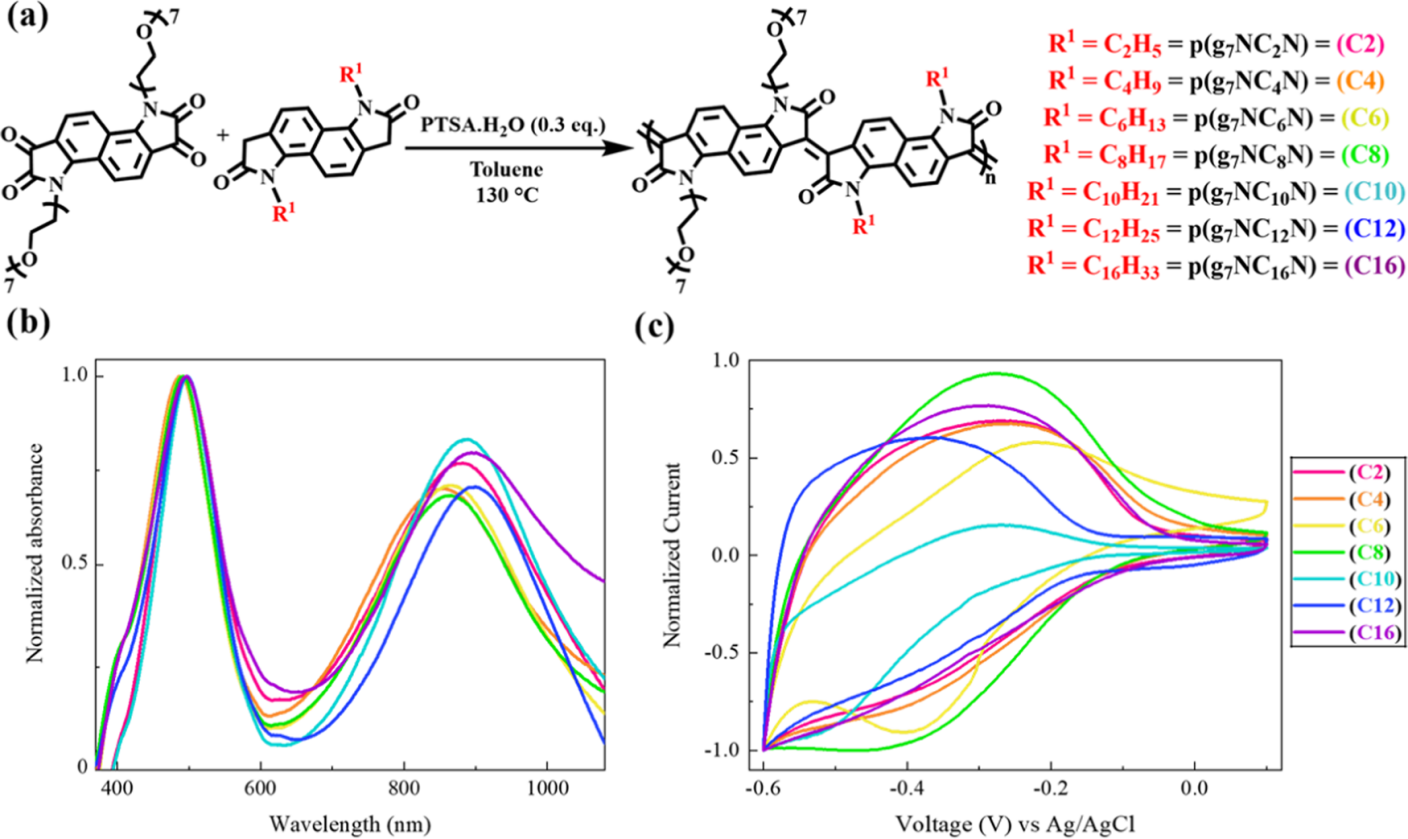
(a) General aldol condensation polymerization conditions and chemical structures of the **p(g**_**7**_**NC***_**n**_***N)** series. (b) Thin-film UV–vis spectra for the entire **p(g**_**7**_**NC***_**n**_***N)** series. (c) Organic electrolyte cyclic voltammetry spectra, obtained in 0.1 M tetrabutylammonium hexafluorophosphate in acetonitrile solution for the entire **p(g**_**7**_**NC***_**n**_***N)** series, acquired at a scan rate of 100 mV s^–1^.

In this study, the hydrophobic alkyl side chain content was synthetically manipulated to produce a series of six novel **PgNaN** derivatives, systematically lengthening the alkyl side chain from ethyl (C_2_H_5_) to hexadecyl (C_16_H_33_) (Figure 3a). These polymers have been more conveniently renamed as **p(g**_**7**_**NC***_**n**_***N)** with N denoting the naphthalene core, g_7_ for the 7-unit ethylene glycol chain and C*n* representing the length of the alkyl side chain tethered to the bis-oxindole monomer unit; for convenience, this will be abbreviated further to (C*n*). For consistency, **PgNaN** will be referred to as **p(g**_**7**_**NC**_**12**_**N)** or (C12).

The glycolated bis-isatin monomer was prepared via a nucleophilic substitution reaction between the bis-isatin core and iodine end-capped seven-unit EG chain.^54^ Bis-oxindole monomers were prepared via a streamlined procedure, avoiding the typical strategy which involves the use of acylchlorides and subsequent reduction with lithium aluminum hydride.^52,53^ Instead, the arylated amine was directly obtained from substitution reactions with the corresponding aryl bromide. Following this, each alkylated amine was subjected to chloroacetylation prior to an intramolecular Heck-type ring closure to afford the series of alkylated bis-oxindole monomers (Figure S1). Owing to the limited solubility of the shortest ethyl side chains, the C_2_H_5_ bis-oxindole monomer had to be synthesized via an alternative route utilizing the harsh Wolff–Kishner reduction, detailed within the Supporting Information (Figure S15).

Each polymer was prepared via a metal-free aldol condensation polymerization, eliminating the use of toxic organostannane monomers and the need for expensive palladium catalysts. Owing to this benign polymerization technique, this class of fully fused polymers are ideal candidates for being interfaced with biological systems.^55^ The high rigidity and polymer backbone planarity, coupled with the A–A motif significantly delocalize and deepen the LUMO frontier orbitals leading to high OECT electron mobilities with deep LUMO energy levels, below −4.2 eV, imparting n-type stability.^27^

## Polymer Properties

Notably, as the length of the alkyl side chain tethered to the bis-oxindole monomer was increased, the molecular weight (MW) of the resultant polymer also increased (Table 1), in a near-linear manner. This can be attributed to the enhanced solubility, during polymerization, imparted by the increase in hydrophobic alkyl side chains, rendering the hexadecyl functionalized (C16) derivative **p(g**_**7**_**NC**_**16**_**N)** as the polymer with the highest number-average molecular weight. The shorter-alkyl-chain polymers were not able to sustain solubility as the chain growth proceeded, leading to precipitation and chain termination. Thus, molecular weight across the series was not fully consistent. Despite this trend, all four polymers with alkyl side chains longer than octyl (C8) have comparable molecular weights and align well with the previously published (C12) derivative.^54^ On modeling the electronic properties of similar fused lactam polymers, it is also evident that even at the molecular weights reported for these polymers, they are beyond the conjugation length expected, and thus there should be no molecular weight dependence on molecular orbital energy levels.^53^ High-resolution scanning tunneling microscopy (STM) images (Figure S53) allow one to distinguish the backbone and the side chains of individual polymers and thus to sequence the molecules by visual inspection. The majority of couplings between successive repeat units is as expected: repeat units with alkyl side chains are followed by repeat units with g_7_ ethylene glycol side chains and vice versa (Figure S53).

**Table 1:** Summary of Polymer Optoelectronic and Physical Properties Including Aqueous Electrolyte Reduction Onset (*E*_red,aq_), Optical Gap (*E*_g,opt_), Ionization Potential (IP), Electron Affinity (EA), Thin-Film Absorption Maximum (λ_max,film_), Number-Average Molecular Weight (*M*_n_), and Dispersity (*Đ*)

polymer	*E*_red,aq_ [V]^a^	*E*_g,opt_ [eV]^b^	IP [eV]^c^	EA [eV]^d^	λ_max,film_ [nm]	*M*_n_ [kDa]^e^	Đ
**p(g_7_NC_2_N)**	–0.07	1.03	5.08	4.26	881	6.2	1.6
**p(g_7_NC_4_N)**	–0.04	0.97	5.10	4.28	854	8.3	1.7
**p(g_7_NC_6_N)**	–0.03	1.04	5.12	4.25	865	10.2	1.5
**p(g_7_NC_8_N)**	–0.13	1.03	5.11	4.26	865	15.0	1.3
**p(g_7_NC_10_N)**	–0.27	1.02	5.12	4.23	889	20.3	1.7
**p(g_7_NC_12_N)**(54)	–0.31	1.05	5.14	4.22	901	20.7	7.8
**p(g_7_NC_16_N)**	–0.35	0.97	5.15	4.29	894	24.2	1.9

a *E*_red,aq_ Calculated using a 0.1 M NaCl in deionized water solution.

b *E*_g,opt_ estimated optical gap using the onset of absorption in thin-film UV–vis spectra *E*_g,opt_ = 1240/ λ_ONSET_.

c IP obtained from photoelectron emission spectroscopy in air (PESA) measurements.

d EA values were calculated from cyclic voltammetry in 0.1 M TBAPF_6_ acetonitrile solution, using the onset of reduction, with respect to Fc/Fc^+^ standard.

e *M*_n_ Gel permeation chromatography (GPC) data obtained vs. polystyrene standards at 40 °C in chloroform.

The optoelectronic properties of the **p(g**_**7**_**NC***_**n**_***N)** series were evaluated through UV–vis spectroscopy, photoelectron emission spectroscopy in air (PESA), and cyclic voltammetry (CV) to determine the optical gap (*E*_g,opt_), ionization potentials (IP), and electron affinities (EA), respectively, and are summarized in Table 1. Each polymer displays two major absorption peaks within their UV–vis spectrum (Figure 3b) with a high energy absorption band in the visible region and a broad absorption band in the near-infrared (NIR) region, resulting in narrow optical gaps ranging from 0.97 to 1.05 eV (Table 1). Utilizing an identical fused naphthalene polymer backbone, previously reported computational time-dependent density functional theory calculations and solvatochromic measurements determined that the broad low-energy NIR band did not have marked charge transfer character.^52^ Instead, both hole and electron wavefunctions were sufficiently delocalized across the entire polymer backbone, which can also be expected for the **p(g**_**7**_**NC***_**n**_***N)** series. Ionization potentials were found to be comparable across the series, while a similar trend was also observed for the electron affinities, which were calculated from thin-film CV (Figure 3c). As anticipated, with no change in side-chain polarity or electronic nature, synthetically altering the length of the hydrophobic alkyl side chain had negligible effects on both the IP and EA of the resultant polymers across the series. Electrochemically calculated electron affinities are sufficiently deep (4.22–4.29 eV) to facilitate electron injection and incur operational stability over 10 cycles in an aqueous medium (Figure S35).^27^ The trend in aqueous electrolyte reduction onsets determined by CV (Table 1), which increased with increasing alkyl side chain content, is likely due to the increased hydrophobicity causing poorer miscibility for solvated ions, across the series from (C2) to (C16).^14,15,50,56^

The electrochemical activity of the series was further investigated by spectroelectrochemical measurements, monitoring the evolution of UV–vis absorbance upon reduction between 0 and −0.6 V. As the applied reduction potential was sequentially increased, the two major neutral absorption bands, localized around 500 nm and 840–900 nm, displayed a gradual reduction in intensity (Figures S36–S42). Simultaneously, the formation of new polaronic features was observed at NIR wavelengths, extending past the 1100 nm range of the detector. Approaching the threshold voltages of each device (Table 2), the neutral absorption bands for each polymer remained present but were reduced in intensity, while the polaronic NIR absorption had also emerged. The degree of polaron formation followed the aforementioned CV findings (Table 1), with an increasingly negative onset of reduction requiring a higher bias to achieve similar levels of charging.

**Table 2:** Summary of OECT Parameters and Material Figures of Merit of the Polymers under Investigation

polymer	μ_e,OECT_ [cm^2^ V^–1^ s^–1^]^a^	C* [F cm^–3^]^b^	μC* [F cm^–1^ V^–1^ s^–1^]^c^	*g*_m_′ [S cm^–1^]	*V*_th_ [mV]^a^
**p(g_7_NC_2_N)**	(2.00 ± 0.41) × 10^–3^	180 ± 16	0.36 ± 0.074	0.069 ± 0.012	230 ± 3.3
**p(g_7_NC_4_N)**	(1.46 ± 0.53) × 10^–3^	126 ± 12	0.18 ± 0.067	0.035 ± 0.013	210 ± 2.9
**p(g_7_NC_6_N)**	(2.29 ± 0.70) × 10^–3^	150 ± 4	0.34 ± 0.111	0.065 ± 0.019	210 ± 6.4
**p(g_7_NC_8_N)**	(6.01 ± 1.87) × 10^–3^	199 ± 27	1.19 ± 0.371	0.240 ± 0.076	250 ± 3.4
**p(g_7_NC_10_N)**	(1.20 ± 0.07) × 10^–2^	153 ± 34	1.83 ± 0.101	0.370 ± 0.023	300 ± 3.0
**p(g_7_NC_12_N)**(54)	(6.50 ± 1.01) × 10^–3^	100 ± 6	0.66 ± 0.113	0.212 ± 0.015	328 ± 5.3
**p(g_7_NC_16_N)**	(3.80 ± 0.59) × 10^–3^	86 ± 11	0.33 ± 0.074	0.047 ± 0.005	360 ± 10.0

a μ_e,OECT_ OECT saturation mobility and threshold voltage (*V*_th_) extracted from fits of *I*_d_^1/2^ vs *V*_G_ plots.

b C* average volumetric capacitance beyond the *V*_th_ determined by electrochemical impedance spectroscopy. Reported uncertainties are one standard deviation, with *n* = 6 devices.

c μC* extracted from the slope of saturated transfer curves at −0.6 V.

## OECT Performance

The effect of synthetically manipulating the length of the hydrophobic alkyl side chain on the resultant electrical properties was investigated by fabricating OECT devices employing each of the polymers as the active channel material. Each device was operated with the “as-cast” polymer, without the need for any additives or subsequent annealing techniques, in an aqueous 0.1 M NaCl solution with an Ag/AgCl gate electrode (Figure 1a). As mentioned in the Introduction section, the common figure of merit to define and compare OECT performance is the μC* value.^10^ However, to further facilitate comparison between the **p(g**_**7**_**NC***_**n**_***N)** series and previously reported materials, the maximum transconductance (*g*_m_) values have been normalized by the channel geometry: *g*_m_′ = *g*_m_/(*Wd*/*L*), where *W* is the width of the channel (100 μm), *L* is the length of the channel (10 μm), and *d* is the thickness of OMIEC channel layer, detailed in the Supporting Information and summarized in Table 2.

**Figure 4:**
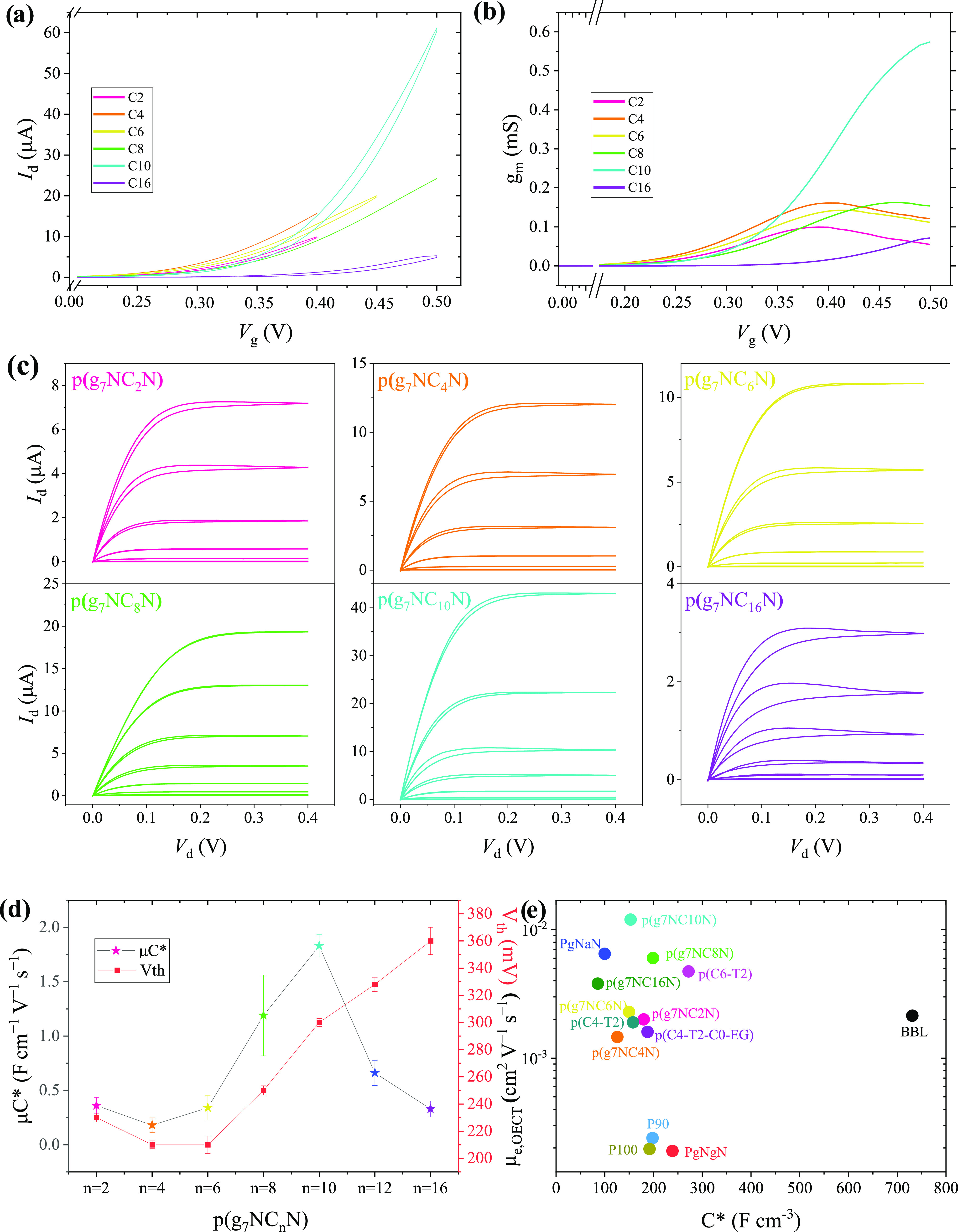
(a) Comparative transfer curves, (b) comparative voltage-dependent transconductance plots, and (c) output curves of the entire **p(g**_**7**_**NC***_**n**_***N)**-based OECT devices. (d) Comparison of μC* figure of merit values and recorded threshold voltages across the **p(g**_**7**_**NC***_**n**_***N)** series. (e) Map of OECT electron mobility versus volumetric capacitance for a selection of previously reported n-type OECT materials using comparable planar device architectures,^57^ compared to the **p(g**_**7**_**NC***_**n**_***N)** series.

The transfer curves, comparative voltage-dependent transconductance plots, and output characteristics of the entire **p(g**_**7**_**NC***_**n**_***N)** series are compared in Figure 4a–c, and the results are summarized in Table 2. Varying the length of the alkyl side chain, thereby altering the overall hydrophobic content, led to marked differences in the resultant OECT device performance. Threshold voltages (*V*_th_) tended to increase as the overall alkyl content increased; for **p(g**_**7**_**NC**_**2**_**N)**, the threshold voltage was extrapolated to be 230 ± 3.3 mV, whereas for **p(g**_**7**_**NC**_**16**_**N)**, this increased by 130 mV to 360 ± 10.0 mV (Figure 4d), on account of the thermodynamic penalty attributed to the presence of solvated ions, which manifests as a larger threshold voltage.^14^

The fully fused π-backbones, composed of only electron-deficient acceptor units, sufficiently delocalize the electron wavefunction and thus LUMO frontier orbitals along the entire polymer backbone. Moreover, the strong interchain interactions, imparted by π–π stacking of the fully fused backbone, enhance both the intrachain and interchain electron transport, exemplified by the state-of-the-art OECT electron mobility of **p(g**_**7**_**NC**_**10**_**N)** (1.20 × 10^–2^ cm^2^ V^–1^ s^–1^). However, the nature of the backbone cannot be the only factor attributed to carrier transport and overall OECT performance, as this factor remains unchanged across the series. Indeed, side-chain composition and the subsequent effects on the polymer microstructure must play an important role in both the OECT electron mobility and volumetric capacitance recorded for each material. Separating the two parameters, first, the volumetric capacitance (C*), measured by electrochemical impedance spectroscopy (EIS) (Figures S43–S44), varied from 86 to 199 F cm^–3^. The **p(g**_**7**_**NC**_**16**_**N)** derivative displayed the lowest C* value, presumably owing to the highest concentration of hydrophobic and insulating alkyl chains and thus the lowest overall hydrophilic content, which is likely to limit swellability.^14,15^ Interestingly, C* peaks for **p(g**_**7**_**NC**_**8**_**N)** and not for the highest hydrophilic content polymer **p(g**_**7**_**NC**_**2**_**N)**, demonstrating that C* is not simply related to the degree of hydrophilic content and suggests that the octyl side chains are well suited to balance both swelling and hydration.^58^ In general, as the length of the alkyl side chain is increased, C* values remain roughly comparable across the series (Table 2); however, upon increasing the hydrophobic content past (C10), up to a factor of 2 decrease is observed for C*, presumably surpassing the tipping point for hydrophilic versus hydrophobic content, limiting ionic miscibility and thus C*.^15^

Second, OECT electron mobility tends to increase as the side-chain length increases from C_2_H_5_ to C_8_H_17_, peaking for **p(g**_**7**_**NC**_**10**_**N)** at 1.20 × 10^–2^ cm^2^ V^–1^ s^–1^, double the value of the next best performers ((C8) and (C12)), before decreasing further for the (C16) derivative. Despite the trend in molecular weights of the polymers highlighted in (Table 1), it is clear that there is not an obvious trend observed in the electron mobility, indicating that molecular weight is unlikely to be the dominant parameter responsible for electrical performance. The variation in electron mobilities is likely due to different levels of swelling and morphological modulation as the ratio of hydrophobic to hydrophilic side chains is altered across the series. Furthermore, the preferential stacking microstructure of each polymer is varied, a trait which will be exacerbated during OECT operation as each material’s ability to accommodate both ions and water molecules from the electrolyte will also vary. These results suggest that the decyl (C10) alkyl chain finds the optimal balance in modulating adverse swelling effects when coupled with the seven-unit EG side-chain repeat unit. The near-parabolic trend in OECT electron mobility values across the polymer series is mirrored in the μC* values. Notably, μC* was increased from the previously reported (C12) derivative **PgNaN** (0.662 ± 0.113 F cm^–1^ V^–1^ s^–1^)^54^ by **p(g**_**7**_**NC**_**8**_**N)** (1.19 ± 0.37 F cm^–1^ V^–1^ s^–1^) and peaked for **p(g**_**7**_**NC**_**10**_**N)** at 1.83 ± 0.10 F cm^–1^ V^–1^ s^–1^, which is one of the highest reported n-type polymer μC* values to date for planar OECTs.^27^ For the case of **p(g**_**7**_**NC**_**10**_**N)**, μC* is dominated by the impressive OECT electron mobility (1.20 × 10^–2^ cm^2^ V^–1^ s^–1^), which surpasses state-of-the-art BBL and naphthalenediimide (NDI)-based materials in comparable planar OECTs by an order of magnitude (Figure 4e).^49,57,59^ The device physics of interdigitated OECTs^60^ differs from that of planar OECTs, and the size, number, and arrangement of interdigitated electrodes in interdigitated OECTs have been shown to change the value and location of peak transconductance in OMIEC materials.^57^ Thus, for the purposes of direct comparison, only planar OECTs of comparable geometries are discussed.

The OECT operational stability of each polymer was established by repeated gate pulsing to generate continuous ON–OFF cycles, as shown in (Figures S45–S50) and aligned well with CV stability measurements (Figure S35). The operational stability of both the lowest and highest hydrophobic content materials, **p(g**_**7**_**NC**_**2**_**N)** and **p(g**_**7**_**NC**_**16**_**N)**, deteriorated upon cycling with on currents stabilizing at ≈75 and 50% of the initial drain current, respectively. However, the rest of the series showed excellent operational stability, retaining up to 100% of the initial drain current upon constant pulsing for 1800 s (Figure S49). Stable long-term high n-type performance has rarely been reported, and these results further exemplify the potential of these fully fused OMIEC materials for practical applications.^27^ Both OECT and CV stability measurements suggest that increasing the side-chain length past the (C12) derivative not only hinders OECT electron mobility and overall performance but also shifts the optimal balance of hydrophilic to hydrophobic content. The same trend is observed when reducing the alkyl-side-chain length below (C8), with (C6), (C4), and (C2) derivatives all showing reduced OECT performance. The ideal balance, which provides a hydrated channel for ionic diffusion, while simultaneously moderating the degree of swelling to retain microstructure integrity, is difficult to achieve; however, the impressive performance and excellent stability of **p(g**_**7**_**NC**_**10**_**N)** accomplish both these feats. Importantly, polymer microstructure is known to greatly influence OECT device performance and can be used to elucidate structure–performance relationships.^46,49^ One well-documented method to investigate the structural properties of OMIECs is through grazing-incidence wide-angle X-ray scattering (GIWAXS) measurements.^38^ The reasons behind the parabolic trend in OECT performance, across the **p(g**_**7**_**NC***_**n**_***N)** series, are illuminated through such measurements and are detailed herein.

## Pristine “As-Cast” GIWAXS Data

**Figure 5:**
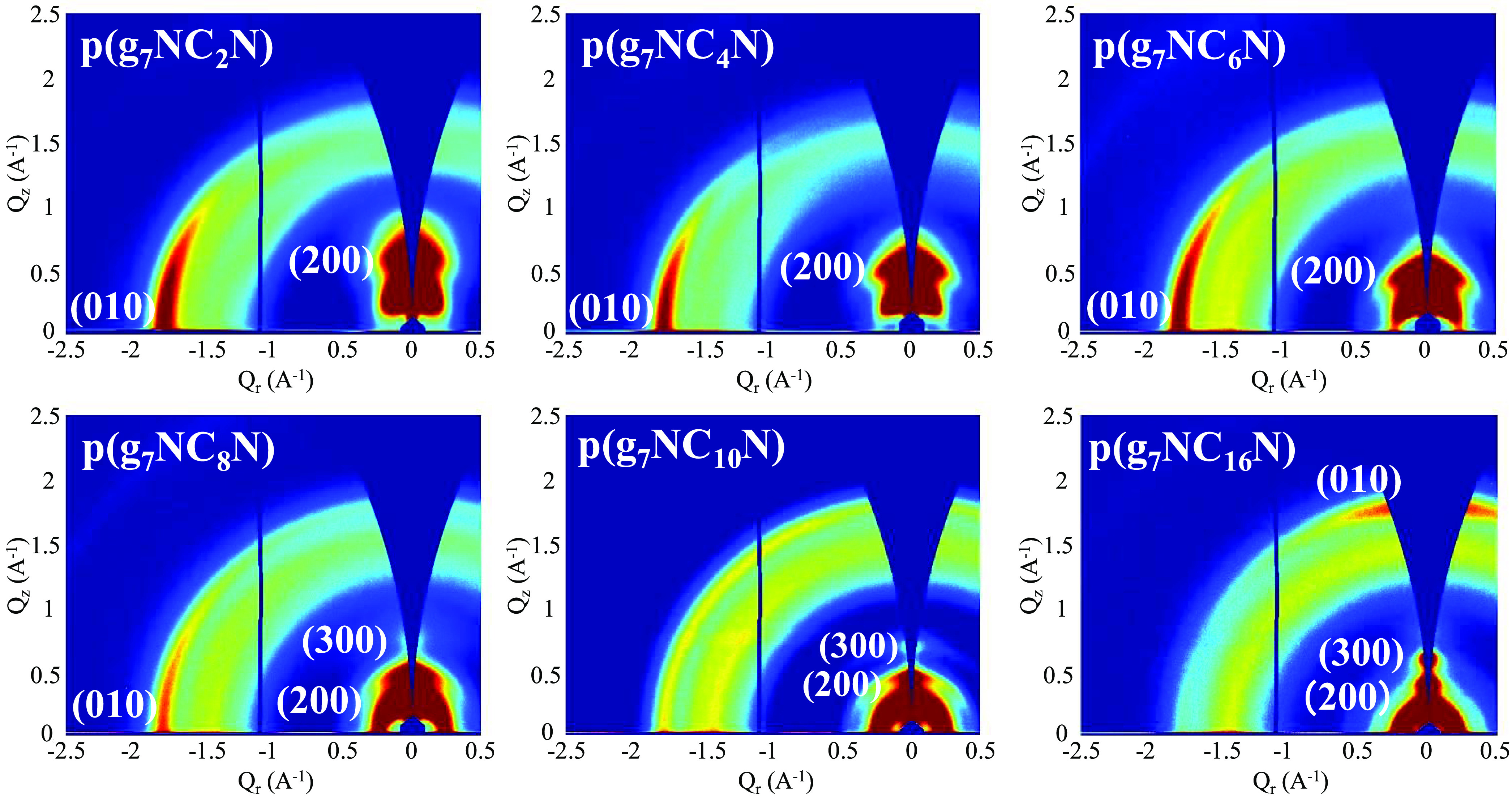
Two-dimensional GIWAXS patterns of the as-cast polymer thin films.

To investigate the effect of modulating the length of the alkyl chain on the polymer structural properties and to correlate these with the OECT device performance data, GIWAXS measurements were collected on the whole polymer series. The morphology of the polymer relative to the substrate is known to affect charge carrier transport within OECT devices and can be used to explain performance trends between materials.^9,10^ Each polymer was processed from the same solvent (CHCl_3_), from solutions of identical concentration (5 mg mL^–1^), and spun-cast at the same rate; see the Supporting Information. Despite this identical method of processing, as the alkyl side chain length increases, the polymer stacking changed from dominantly edge-on (C2, C4, C6, C8) to mixed edge-on and face-on (C10, C12), and finally to preferentially face-on (C16), as demonstrated in the 2D-GIWAXS patterns (Figure 5). These findings are in accordance with the literature, which has demonstrated that polymers with higher solubilities in the processing solvent tend not to form a high aspect ratio, discotic lamella aggregates in solution, but instead prefer to orient face-on rather than edge-on with respect to the substrate, orienting in the direction of fluid flow.^61^ We note that these GIWAXS observations further corroborate the trend in molecular weight, which also increased as the length of the alkyl chain increased, owing to the improved solubility imparted from longer alkyl chains. In addition, increasing the alkyl side chain length led to a steady increase in the lamellar spacing, from 20.6 (C2) to 32.2 Å (C16), demonstrated in the out-of-plane line cuts (Figure S52).

Each polymer, apart from **p(g**_**7**_**NC**_**2**_**N)**, displayed (010) scattering peaks (π-stacking) in both the in-plane and out-of-plane directions. The shortest π–π spacing was observed for **p(g**_**7**_**NC**_**8**_**N)**, for both in-plane (3.47 Å) and out-of-plane (3.49 Å) line cuts. However, varying the length of the alkyl side chain had a small effect on the π–stacking distances across the series, with all values obtained within a 0.06 Å range. More specifically for OECT devices, which operate in the bulk electrochemical regime, a mixed edge-on face-on orientation has been shown to improve three-dimensional (3D) charge transport.^38,47^

However, the OECT performance dependence on alkyl side chain length is a complicated interplay of ion (and solvating water) miscibility, molecular weight, and microstructural effects. Ion miscibility is necessary for electronic doping and manifests in electrochemical reduction onset in aqueous media and OECT threshold. While there is the commonly held assumption that increased molecular weight leads to improved electrical transport, here, ion miscibility and molecular weight are at cross-purposes. The increased alkyl chain length leads to increased MW (likely due to improved solubility during polymerization) but also decreased ion miscibility.

For the short-alkyl-chain-length polymers (C2, C4, C6), the variations in electron mobility, threshold voltage, aqueous electrochemical reduction onset, C*, and μC* show no strong trend compared to the rest of the series. At intermediate-alkyl-chain-length polymers (C8, C10), large successive increases in electron mobility and μC* are observed, with both maximized in the C10 polymer.

From C8 to C16, there is a consistent trend of increasing threshold voltage and decreasing C*, which is consistent with decreasing ion/water miscibility. However, the mobility precipitously drops (by almost 70%) from the C10 peak to the longest-alkyl-chain-length C16 polymer. Looking toward the microstructure to understand this unexpected trend, the peak electron mobility polymer presents a mixed edge-on and face-on microstructure that has previously been shown to correlate with peak OECT performance.^38,47^ The GIWAXS patterns for **p(g**_**7**_**NC**_**10**_**N)** corroborate this theory and the highest OECT performance, specifically the n-type OECT mobility, of the (C10) derivative can be explained by the preferential mixed edge-on and face-on stacking of the material.^14^

## Thermoelectric Investigation

The commonly measured figure of merit for thermoelectric materials is the power factor (PF = *S*^2^σ), determined by the Seebeck coefficient (*S*) and the electrical conductivity (σ = *qn*μ), where *q* is the elementary charge, *n* is the number density of charge carriers, and μ is the charge carrier mobility.^34^ As both *S* and σ are influenced by the charge carrier concentration in OSCs, OECTs with controlled or tunable charge carrier concentrations have the capability to serve as a unique platform for the investigation of potential materials for thermoelectric devices.^62^ Therefore, intuitively, the OECT performance, especially the high electron mobility outlined above (Table 2), would suggest that the fused mixed conduction polylactams are promising n-type thermoelectric candidates. Indeed, the performance of ladder-type polymers for n-type thermoelectric applications has improved considerably in recent years with PF values on the order of 10^0^–10^1^ μW m^–1^ K^–2^ having been reported.^32^ However, elucidation of structure–property relationships of conjugated ladder-type polymers for n-type OTEs has remained relatively unexplored, with a few studies evaluating the effects of rational chemical modification in relation to device performance.^63−65^ To address this, first, the electrical conductivity of each polymer was investigated. N-DMBI was chosen to solution-dope the polymers, thus rendering the doping process compatible with low-cost roll-to-roll printing techniques.^66^ Each polymer was also vapor-doped using the strong reducing agent tetrakis(dimethylamino)ethylene (TDAE), detailed in the Supporting Information (Figure S54), and maximum performance metrics are summarized in Table 3.^67^

**Table 3:** Summary of OTE Parameters and Material Figures of Merit for N-DMBI- and TDAE-Doped **p(g**_**7**_**NC***_**n**_***N)** Samples

	N-DMBI		TDAE	
polymer	σ_max_ [S cm^–1^]	PF_max_ [μW m^–1^ K^–2^]	σ_max_ [S cm^–1^]	PF_max_ [μW m^–1^ K^–2^]
**p(g_7_NC_2_N)**	4.69 ± 0.23	5.90 ± 0.36	0.43 ± 0.11	0.46 ± 0.17
**p(g_7_NC_4_N)**	7.67 ± 0.29	10.4 ± 0.52	0.31 ± 0.15	0.31 ± 0.11
**p(g_7_NC_6_N)**	0.99 ± 0.06	2.06 ± 0.08	0.21 ± 0.14	0.58 ± 0.19
**p(g_7_NC_8_N)**	1.24 ± 0.09	2.93 ± 0.15	0.33 ± 0.11	0.90 ± 0.21
**p(g_7_NC_10_N)**	1.70 ± 0.14	5.11 ± 0.13	1.39 ± 0.19	2.63 ± 0.28
**p(g_7_NC_12_N)**(54)	0.81 ± 0.15	1.24 ± 0.11	0.28 ± 0.08	0.72 ± 0.16
**p(g_7_NC_16_N)**	0.30 ± 0.01	0.84 ± 0.12	0.22 ± 0.10	0.60 ± 0.14

**Figure 6:**
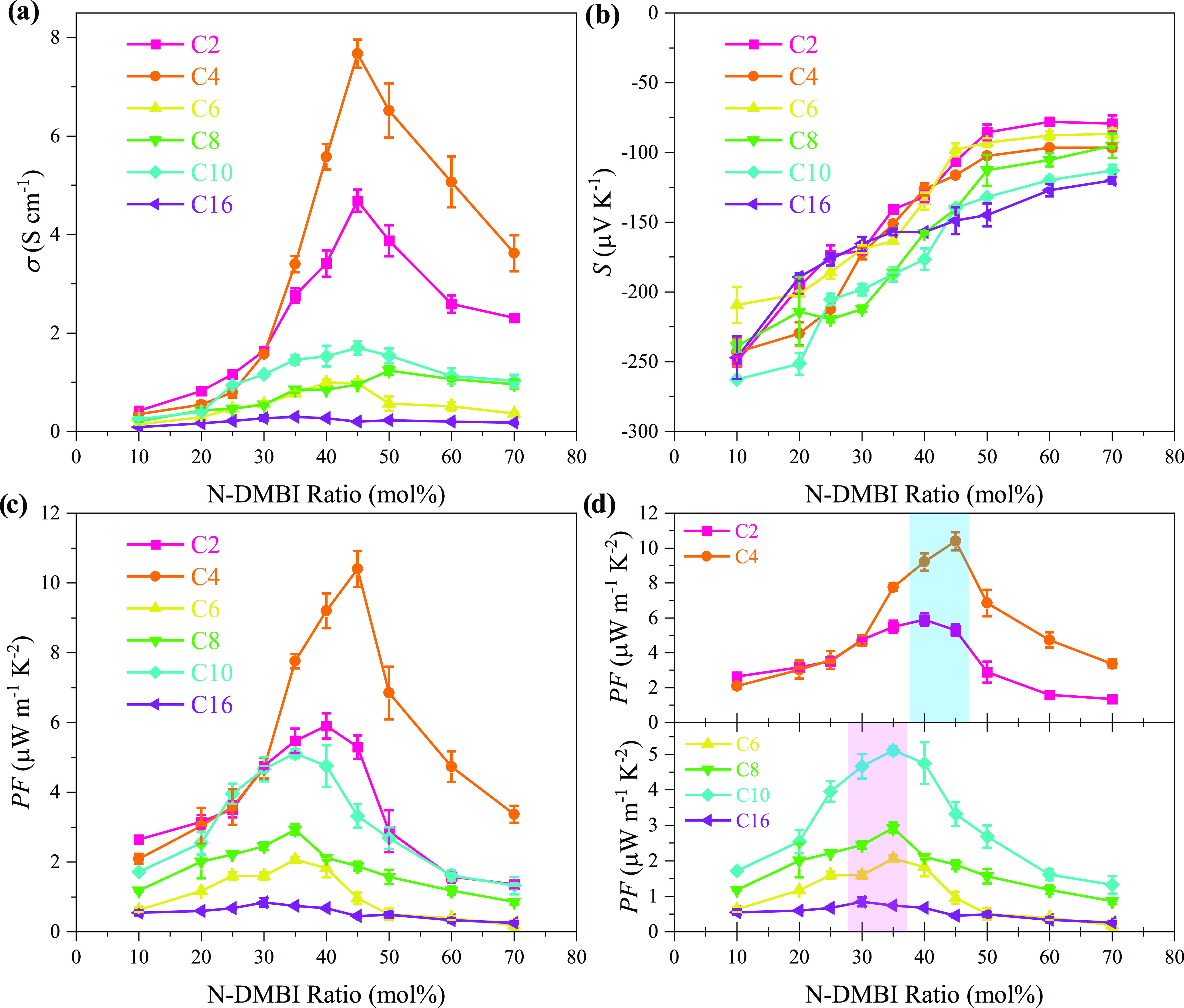
(a) Electrical conductivities, (b) Seebeck coefficients, and (c) power factors recorded for the **p(g**_**7**_**NC***_**n**_***N)** series as a function of N-DMBI dopant ratio employed. (d) Blue and pink highlights demonstrate the difference between the 30–35 mol % (C2–C4) and 40–45 mol % (C6, C8, C10, C16) dopant concentration maximum PF_max_ regimes.

As highlighted in (Figure 6a), the electrical conductivity for each polymer is lowest at the minimum N-DMBI dopant molar ratio (∼10 mol %). Upon increasing the dopant concentration, the electrical conductivity increases for each polymer, with **p(g**_**7**_**NC**_**10**_**N)** peaking at 1.70 ± 0.14 S cm^–1^ (at 35 mol %), **p(g**_**7**_**NC**_**2**_**N)** peaking at 4.69 ± 0.23 S cm^–1^ (at 45 mol %), and finally the highest σ was recorded for **p(g**_**7**_**NC**_**4**_**N)** peaking at 7. 67 ± 0.29 S cm^–1^ (at 45 mol %). Markedly, the trend in maximum electrical conductivity (σ_max_) did not follow the trend in electron mobility measured in OECT devices. **p(g**_**7**_**NC**_**10**_**N)** recorded an electron mobility that was an order of magnitude larger than the rest of the series; however, both (C2) and (C4) derivatives show ∼2.7- and 4.5-fold increases in electrical conductivity with respect to (C10), respectively. The observed σ_max_ values for both **p(g**_**7**_**NC**_**2**_**N)** and **p(g**_**7**_**NC**_**4**_**N)** can be explained by the reduction in alkyl side chain length leading to a preferential edge-on polymer microstructure (detailed later) increasing dopant miscibility and transport properties, subsequently improving electrical conductivity.^32^ Conversely, while **p(g**_**7**_**NC**_**10**_**N)** has a much higher OECT electron mobility (Table 2), we postulate that increasing dopant concentration heavily disturbs the polymer microstructure limiting charge transport, while also assuming weaker dopant miscibility due to the increased amorphous hydrophobic regions imparted by the lengthened decyl side chains.^68^ This theory is supported by the relatively similar σ values observed across the three materials at a dopant concentration of 25 mol %, upon which increasing the dopant concentration to ∼50 mol % for **p(g**_**7**_**NC**_**10**_**N)** has a comparatively minimal impact on conductivity. Conversely, for both (C2) and (C4) derivatives, the electrical conductivity significantly increases at 45 mol %, only decreasing when exceeding 50 mol %, presumably due to the difference in preferential polymer stacking microstructure, improved dopant miscibility, and reduced microstructure disruption compared to (C10).

These findings were corroborated by the results of sequential-vapor doping, using the powerful electron-donor TDAE, in which **p(g**_**7**_**NC**_**10**_**N)** showed the highest performance, across the series, reaching a PF of 2.63 μW m^–1^ K^–2^ (Table 3). Upon initial vapor deposition, the polymers retain the majority of their undoped properties, such as the order of magnitude higher electron mobility of **p(g**_**7**_**NC**_**10**_**N)**, which leads to a peak electrical conductivity of 1.4 S cm^–1^. As expected, prolonged exposure to TDAE vapor led to lower conductivities and poorer thermoelectric performances, across the entire series, principally due to disruption of polymer morphology and hence lower polymer crystallinity.^67^ The difference in highest-performing materials between chemical and vapor doping (Table 3) suggests that, as outlined above, chemical doping is much more impactful on the polymer microstructure and is dependent on the degree of hydrophobic insulating side-chain content. Notably, the highest-performing OECT material **p(g**_**7**_**NC**_**10**_**N)** was more readily disrupted upon the inclusion of a lower concentration of N-DMBI compared to **p(g**_**7**_**NC**_**2**_**N)** and **p(g**_**7**_**NC**_**4**_**N)**. Conversely, vapor doping, which has a much milder effect on polymer morphology and microstructure, at low dopant levels, ensures that **p(g**_**7**_**NC**_**10**_**N)** devices showed the highest TDAE vapor-doped OTE performance, matching the observed trend in OECT performance. These results highlight the key differences between doping processes and the measurable effects these have on the resultant polymer microstructure and subsequent device performance metrics.^40^

The Seebeck coefficients (thermopower) of all materials were measured, upon doping across a range of N-DMBI concentrations (Figure 6b). As expected, all members of the series displayed negative Seebeck coefficients, indicative of the dominant electron charge carrier, confirming the n-type nature of the series. The magnitudes of the Seebeck coefficients were the highest (most negative), for each polymer, at the lowest dopant ratio of 10 mol % and decreased progressively upon increasing the dopant ratio. As expected, the evolution of σ and *S* as a function of dopant ratio followed an opposite trend, thus signifying that the maximum power factor for the polymers was obtained at a dopant concentration that would best balance the two quantities, as highlighted in Figure 6c. The maximum PF values were recorded at different N-DMBI dopant concentrations, with longer-alkyl-chain derivatives **p(g**_**7**_**NC**_**8**_**N)**, **p(g**_**7**_**NC**_**10**_**N)**, and **p(g**_**7**_**NC**_**16**_**N)** peaking at 30–35 mol %, whereas shorter-alkyl-chain derivatives **p(g**_**7**_**NC**_**2**_**N)** and **p(g**_**7**_**NC**_**4**_**N)** peaked at 40–45 mol % (Table 3), following the aforementioned trend in electrical conductivity. The maximum power factor was achieved with **p(g**_**7**_**NC**_**4**_**N)**, at 10.40 ± 0.52 μW m^–1^ K^–2^. The PF values recorded are among the highest reported for n-type OTE materials to date and are state-of-the-art for fully fused polylactam materials.^27,53,69^

These results surpass those of previously published, fully alkylated polylactams, increasing σ_max_ by an order of magnitude and a greater than 3-fold increase in PF.^53^ The impressive performance increase, from modulating the hydrophobic alkyl content and replacing 50% of the side chains with a hydrophilic seven-unit glycol chain, supports that the inclusion of polar side chains improves the host/dopant miscibility and significantly increases the doping efficiency compared to the baseline case of alkyl side chains on both monomer units.^32,45^ Combining these studies suggests that the inclusion of polar side chains is indeed a valuable design strategy to improve thermoelectric performance, adding more weight to the ever-growing toolbox of OMIEC applications.

## OTE-Doped GIWAXS Investigation

**Figure 7:**
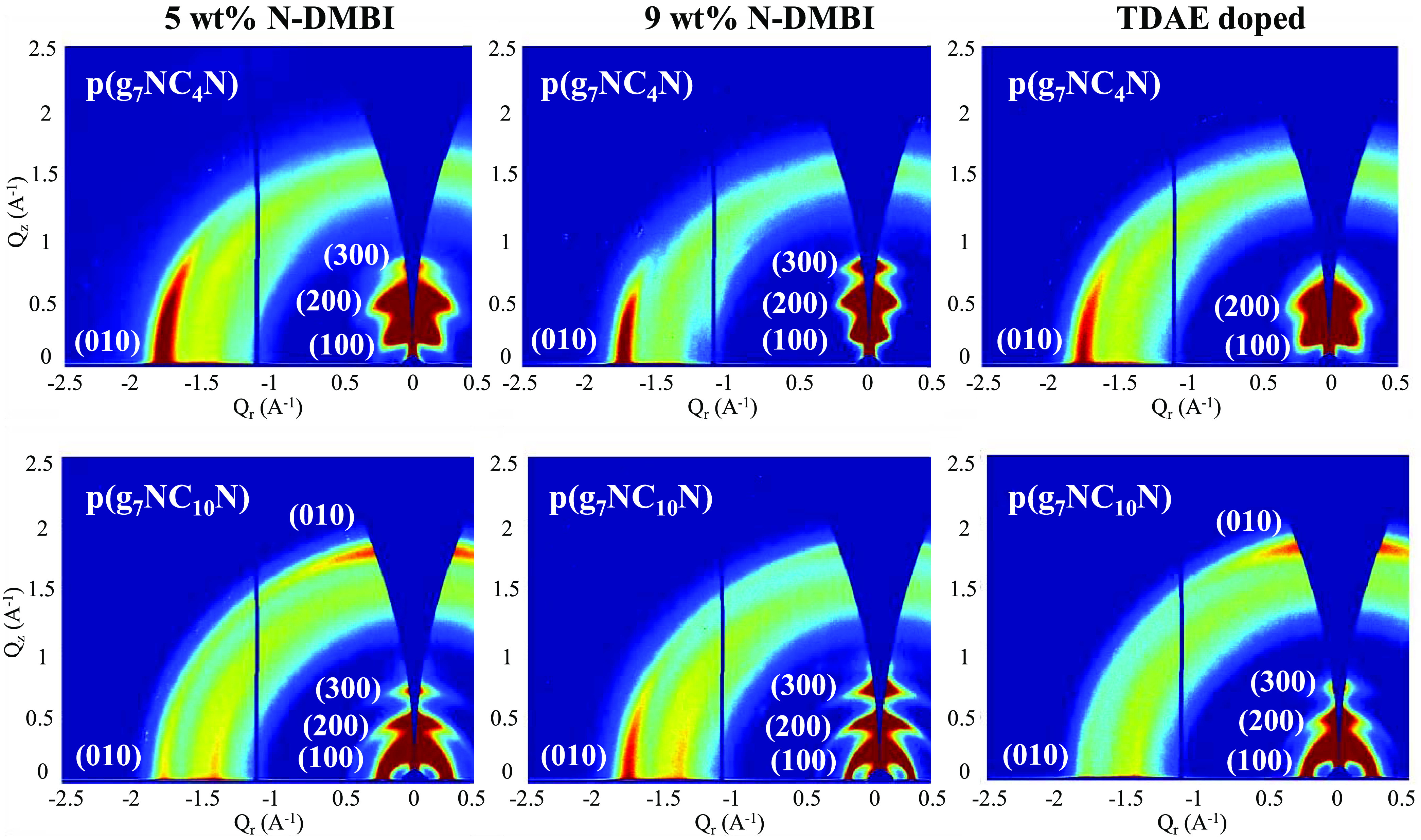
Two-dimensional GIWAXS patterns of **p(g**_**7**_**NC**_**4**_**N)** (top row) and **p(g**_**7**_**NC**_**10**_**N)** (bottom row) thin films, each doped with 35 mol % N-DMBI, 45 mol % N-DMBI, and TDAE, respectively.

In analogy to the OECT discussion above, GIWAXS was again utilized to investigate and explain the trend in thermoelectric performance across the **p(g**_**7**_**NC***_**n**_***N)** series. Here, the highest-performing OTE material **p(g**_**7**_**NC**_**4**_**N)** was directly compared to the highest-performing OECT material **p(g**_**7**_**NC**_**10**_**N)**. For each material, GIWAXS data were obtained from a neat “as-cast” film, films doped with 35 and 45 mol % N-DMBI, respectively, and finally from samples vapor-doped with TDAE (Figure 7).

The overall polymer microstructure organization remains unchanged for **p(g**_**7**_**NC**_**4**_**N)**, which maintains a preferential edge-on orientation in the undoped, 35 mol %, 45 mol %, and TDAE-doped states. However, as the N-DMBI dopant concentration is increased, the correlation length is also increased, suggesting enhanced ordering within polymer crystallites (Table S4). Additionally, edge-on orientation is preferential for facilitating electron transport, which occurs within crystalline regions and, as such, both factors combined lead to an increased electrical conductivity and, subsequently, to a maximum PF of 10.4 μW m^–1^ K^–2^ for **p(g**_**7**_**NC**_**4**_**N)**.^37,39^ In contrast, for **p(g**_**7**_**NC**_**10**_**N)**, as the dopant concentration is increased from 35 to 45 mol % N-DMBI, the polymer microstructure shifts from a mixed edge-on face-on orientation to purely edge-on orientation. While this orientation should theoretically be more beneficial for charge transport, the overall disruption to the polymer’s preferred mixed-orientation packing structure suggests that beyond 35 mol % dopant concentration, adverse dopant–polymer interactions occur, on account of the decreasing PF. This theory is supported by the TDAE vapor-doped GIWAXS data, which show that the packing motif of both **p(g**_**7**_**NC**_**4**_**N)** and **p(g**_**7**_**NC**_**10**_**N)** remains relatively unchanged when doped; here, the high electron transport properties of the extended (C10) alkyl chain derivative, which were demonstrated within OECT devices, dominate the OTE performance. These results are further corroborated by previous studies, which have highlighted the importance of polymer–dopant interactions.^32,44^ The doped GIWAXS data suggest that the shortened (C4) alkyl-chain derivative **p(g**_**7**_**NC**_**4**_**N)** enables more N-DMBI dopant molecules to intercalate within the polymer domains without disrupting the packing structure As such, dopant treatment can be endured up to 45 mol %, where the increased number of doped states leads to an increase in overall OTE performance (Figure 6). Conversely, the highest-performing OECT material **p(g**_**7**_**NC**_**10**_**N)** suffers from adverse polymer–dopant interactions, at elevated dopant concentrations, with GIWAXS of doped films showing a clear disruption of the packing microstructure explaining the drop in OTE performance from 35 mol %, where the adverse effects are not as prevalent, to 45 mol % N-DMBI (Figure 7).

## Conclusions

In summary, a series of six n-type fused lactam mixed conduction polymers, modulating the hydrophobic (alkyl)-to-hydrophilic (glycol) ratio, were synthesized for OECT and OTE device applications. Notably, this report is the first of its kind where one series is directly compared as the channel layer material in both OECT and OTE devices. Compared to traditional transition-metal-catalyzed polymerization reactions, the materials developed herein were obtained via a benign metal-free aldol polycondensation reaction, avoiding the use of expensive precious-metal-based catalysts and any highly toxic organometallic or organotin synthetic intermediates. Modulating the hydrophobic alkyl side chain density ultimately resulted in the state-of-the-art n-type OECT and OTE performance. For OECT-based applications, the optimal balance between the polar seven-unit glycol chain was met by employing decyl side chains with **p(g**_**7**_**NC**_**10**_**N)** recording an impressive electron mobility of (1.20 ± 0.07) × 10^–2^ cm^2^ V^–1^ s^–1^ and a state-of-the-art n-type polymer μC* figure of merit value of 1.83 F cm^–1^ V^–1^ s^–1^. Reducing the overall alkyl content to afford **p(g**_**7**_**NC**_**4**_**N)** allowed for a high dopant concentration to be employed without disrupting the polymer microstructure, recording a maximum electrical conductivity of 7.67 S cm^–1^ and a power factor of 10.4 μW m^–1^ K^–2^. The PF value recorded for **p(g**_**7**_**NC**_**4**_**N)** is among the highest reported for n-type polymers and surpasses previously reported fully alkylated fused polylactam polymers by more than 3 times.^27,53^

Evaluating both the OECT and OTE performance across the **p(g**_**7**_**NC***_**n**_***N)** series clearly demonstrates that the A–A polylactam motif can operate as a high-performing channel layer material in both mixed conduction applications. However, the impact of molecular weight remains difficult to interpret and should also be considered. Moreover, while a single synthetic design principle can be utilized, to obtain the optimal performing OECT and OTE channel materials, synthetic nuance relating to the polymer’s resultant microstructure and dopant miscibility must be carefully controlled. Furthermore, the simple modulation of alkyl side chain content presents a more straightforward strategy than those previously employed, whereby entire side chains were redesigned when transitioning from OTFT to OECT applications and where high charge carrier mobility within OTFT devices generally does not translate to high performance in OECTs.^27^ As such, the bulk transportation nature within OECTs renders this device a much more viable and informative platform to draw from for OTE research, which also operates in a bulk regime, compared to the interfacial behavior of OTFT devices. The OECT and OTE results detailed throughout allow for structure–property–performance relationships to be elucidated for both mixed conduction applications, whereas previous publications have focused exclusively on the OECT or OTE performance of OMIECs independently. The results reported in this study highlight that selectively altering the hydrophobic alkyl content, for this series of fused all acceptor polylactams, is a highly effective molecular design strategy to optimize the OECT and OTE device performance, thus also providing new insights into the molecular design guidelines for the next generation of high-performance n-type materials. The expected biocompatibility conferred from the benign metal-free aldol polycondensation, along with the state-of-the-art device performance further suggests that fully fused all acceptor polylactams are a promising and exciting platform for future OECT and OTE applications.

## Supporting Information Available

Including monomer and polymer syntheses and characterization: UV–vis spectra, GPC traces, electrochemical characterization of the polymers, spectroelectrochemical measurements, OECT measurements, STM images, OTE measurements, and GIWAXS measurements (PDF)

## References

[ref1] Moser M.; Ponder J. F.; Wadsworth A.; Giovannitti A.; McCulloch I. Materials in Organic Electrochemical Transistors for Bioelectronic Applications: Past, Present, and Future. Adv. Funct. Mater. 2019, 29, 1807033 10.1002/adfm.201807033.

[ref2] Pappa A. M.; Ohayon D.; Giovannitti A.; Maria I. P.; Savva A.; Uguz I.; Rivnay J.; McCulloch I.; Owens R. M.; Inal S. Direct Metabolite Detection with an N-Type Accumulation Mode Organic Electrochemical Transistor. Sci. Adv. 2018, 4, eaat0911 10.1126/sciadv.aat0911. 29942860 PMC6014717

[ref3] Rivnay J.; Owens R. M.; Malliaras G. G. The Rise of Organic Bioelectronics. Chem. Mater. 2014, 26, 679–685. 10.1021/cm4022003.

[ref4] Kelly K. L.; Coronado E.; Zhao L. L.; Schatz G. C. The Optical Properties of Metal Nanoparticles: The Influence of Size, Shape, and Dielectric Environment. J. Phys. Chem. B 2003, 107, 668–677. 10.1021/jp026731y.

[ref5] Pappa A.-M.; Curto V. F.; Braendlein M.; Strakosas X.; Donahue M. J.; Fiocchi M.; Malliaras G. G.; Owens R. M. Organic Transistor Arrays Integrated with Finger-Powered Microfluidics for Multianalyte Saliva Testing. Adv. Healthc. Mater. 2016, 5, 2295–2302. 10.1002/adhm.201600494. 27385673

[ref6] Paulsen B. D.; Fabiano S.; Rivnay J. Mixed Ionic-Electronic Transport in Polymers. Annu. Rev. Mater. Res. 2021, 51, 73–99. 10.1146/annurev-matsci-080619-101319.

[ref7] Marks A.; Griggs S.; Gasparini N.; Moser M. Organic Electrochemical Transistors: An Emerging Technology for Biosensing. Adv. Mater. Interfaces 2022, 9, 2102039 10.1002/admi.202102039.

[ref8] Rivnay J.; Inal S.; Collins B. A.; Sessolo M.; Stavrinidou E.; Strakosas X.; Tassone C.; Delongchamp D. M.; Malliaras G. G. Structural Control of Mixed Ionic and Electronic Transport in Conducting Polymers. Nat. Commun. 2016, 7, 11287 10.1038/ncomms11287. 27090156 PMC4838877

[ref9] Friedlein J. T.; McLeod R. R.; Rivnay J. Device Physics of Organic Electrochemical Transistors. Org. Electron. 2018, 63, 398–414. 10.1016/j.orgel.2018.09.010.

[ref10] Inal S.; Malliaras G. G.; Rivnay J. Benchmarking Organic Mixed Conductors for Transistors. Nat. Commun. 2017, 8, 1767 10.1038/s41467-017-01812-w. 29176599 PMC5701155

[ref11] Kukhta N. A.; Marks A.; Luscombe C. K. Molecular Design Strategies toward Improvement of Charge Injection and Ionic Conduction in Organic Mixed Ionic–Electronic Conductors for Organic Electrochemical Transistors. Chem. Rev. 2021, 122, 4325–4355. 10.1021/acs.chemrev.1c00266. 34902244 PMC8874907

[ref12] Paudel P. R.; Tropp J.; Kaphle V.; Azoulay J. D.; Lüssem B. Organic Electrochemical Transistors – from Device Models to a Targeted Design of Materials. J. Mater. Chem. C 2021, 9, 9761–9790. 10.1039/D1TC01601F.

[ref13] Keene S. T.; Pol T. P. A.; Zakhidov D.; Weijtens C. H. L.; Janssen R. A. J.; Salleo A.; Burgt Y. Enhancement-Mode PEDOT:PSS Organic Electrochemical Transistors Using Molecular De-Doping. Adv. Mater. 2020, 32, 2000270 10.1002/adma.202000270. 32202010

[ref14] Savva A.; Hallani R.; Cendra C.; Surgailis J.; Hidalgo T. C.; Wustoni S.; Sheelamanthula R.; Chen X.; Kirkus M.; Giovannitti A.; Salleo A.; McCulloch I.; Inal S. Balancing Ionic and Electronic Conduction for High-Performance Organic Electrochemical Transistors. Adv. Funct. Mater. 2020, 30, 1907657 10.1002/adfm.201907657.

[ref15] Giovannitti A.; Maria I. P.; Hanifi D.; Donahue M. J.; Bryant D.; Barth K. J.; Makdah B. E.; Savva A.; Moia D.; Zetek M. M. M.; Barnes P. R. F. F.; Reid O. G.; Inal S.; Rumbles G.; Malliaras G. G.; Nelson J.; Rivnay J.; Mcculloch I. The Role of the Side Chain on the Performance of N-Type Conjugated Polymers in Aqueous Electrolytes. Chem. Mater. 2018, 30, 2945–2953. 10.1021/acs.chemmater.8b00321. 29780208 PMC5953566

[ref16] Wang Y.; Zeglio E.; Liao H.; Xu J.; Liu F.; Li Z.; Maria I. P.; Mawad D.; Herland A.; McCulloch I.; Yue W. Hybrid Alkyl–Ethylene Glycol Side Chains Enhance Substrate Adhesion and Operational Stability in Accumulation Mode Organic Electrochemical Transistors. Chem. Mater. 2019, 31, 9797–9806. 10.1021/acs.chemmater.9b03798.

[ref17] Nielsen C. B.; Giovannitti A.; Sbircea D.-T. T.; Bandiello E.; Niazi M. R.; Hanifi D. A.; Sessolo M.; Amassian A.; Malliaras G. G.; Rivnay J.; McCulloch I. Molecular Design of Semiconducting Polymers for High-Performance Organic Electrochemical Transistors. J. Am. Chem. Soc. 2016, 138, 10252–10259. 10.1021/jacs.6b05280. 27444189 PMC4991841

[ref18] Moser M.; Hidalgo T. C.; Surgailis J.; Gladisch J.; Ghosh S.; Sheelamanthula R.; Thiburce Q.; Giovannitti A.; Salleo A.; Gasparini N.; Wadsworth A.; Zozoulenko I.; Berggren M.; Stavrinidou E.; Inal S.; McCulloch I. Side Chain Redistribution as a Strategy to Boost Organic Electrochemical Transistor Performance and Stability. Adv. Mater. 2020, 32, 2002748 10.1002/adma.202002748. 32754923

[ref19] Kim S.-M.; Kim C.-H.; Kim Y.; Kim N.; Lee W.-J.; Lee E.-H.; Kim D.; Park S.; Lee K.; Rivnay J.; Yoon M.-H. Influence of PEDOT:PSS Crystallinity and Composition on Electrochemical Transistor Performance and Long-Term Stability. Nat. Commun. 2018, 9, 3858 10.1038/s41467-018-06084-6. 30242224 PMC6155079

[ref20] Giovannitti A.; Sbircea D.-T.; Inal S.; Nielsen C. B.; Bandiello E.; Hanifi D. A.; Sessolo M.; Malliaras G. G.; McCulloch I.; Rivnay J. Controlling the Mode of Operation of Organic Transistors through Side-Chain Engineering. Proc. Natl. Acad. Sci. U.S.A. 2016, 113, 12017–12022. 10.1073/pnas.1608780113. 27790983 PMC5087003

[ref21] Pappa A.-M.; Parlak O.; Scheiblin G.; Mailley P.; Salleo A.; Owens R. M. Organic Electronics for Point-of-Care Metabolite Monitoring. Trends Biotechnol. 2018, 36, 45–59. 10.1016/j.tibtech.2017.10.022. 29196057

[ref22] Moia D.; Giovannitti A.; Szumska A. A.; Maria I. P.; Rezasoltani E.; Sachs M.; Schnurr M.; Barnes P. R. F. F.; McCulloch I.; Nelson J. Design and Evaluation of Conjugated Polymers with Polar Side Chains as Electrode Materials for Electrochemical Energy Storage in Aqueous Electrolytes. Energy Environ. Sci 2019, 12, 1349–1357. 10.1039/C8EE03518K.

[ref23] Sun H.; Vagin M.; Wang S.; Crispin X.; Forchheimer R.; Berggren M.; Fabiano S. Complementary Logic Circuits Based on High-Performance n-Type Organic Electrochemical Transistors. Adv. Mater. 2018, 30, 1704916 10.1002/adma.201704916. 29318706

[ref24] Giovannitti A.; Nielsen C. B.; Sbircea D.-T.; Inal S.; Donahue M.; Niazi M. R.; Hanifi D. A.; Amassian A.; Malliaras G. G.; Rivnay J.; McCulloch I. N-Type Organic Electrochemical Transistors with Stability in Water. Nat. Commun. 2016, 7, 13066 10.1038/ncomms13066. 27713414 PMC5059848

[ref25] Lin P.; Yan F.; Yu J.; Chan H. L. W.; Yang M. The Application of Organic Electrochemical Transistors in Cell-Based Biosensors. Adv. Mater. 2010, 22, 3655–3660. 10.1002/adma.201000971. 20661950

[ref26] Lin P.; Luo X.; Hsing I.-M.; Yan F. Organic Electrochemical Transistors Integrated in Flexible Microfluidic Systems and Used for Label-Free DNA Sensing. Adv. Mater. 2011, 23, 4035–4040. 10.1002/adma.201102017. 21793055

[ref27] Griggs S.; Marks A.; Bristow H.; McCulloch I. N-Type Organic Semiconducting Polymers: Stability Limitations, Design Considerations and Applications. J. Mater. Chem. C 2021, 9, 8099–8128. 10.1039/D1TC02048J. PMC8264852 34277009

[ref28] De Leeuw D. M.; Simenon M. M. J.; Brown A. R.; Einerhand R. E. F. Stability of N-Type Doped Conducting Polymers and Consequences for Polymeric Microelectronic Devices. Synth. Met 1997, 87, 53–59. 10.1016/S0379-6779(97)80097-5.

[ref29] Zhan X.; Facchetti A.; Barlow S.; Marks T. J.; Ratner M. A.; Wasielewski M. R.; Marder S. R. Rylene and Related Diimides for Organic Electronics. Adv. Mater. 2011, 23, 268–284. 10.1002/adma.201001402. 21154741

[ref30] Sun H.; Gerasimov J.; Berggren M.; Fabiano S. N-Type Organic Electrochemical Transistors: Materials and Challenges. J. Mater. Chem. C 2018, 6, 11778–11784. 10.1039/C8TC03185A.

[ref31] Takeda Y.; Hayasaka K.; Shiwaku R.; Yokosawa K.; Shiba T.; Mamada M.; Kumaki D.; Fukuda K.; Tokito S. Fabrication of Ultra-Thin Printed Organic TFT CMOS Logic Circuits Optimized for Low-Voltage Wearable Sensor Applications. Sci. Rep. 2016, 6, 25714 10.1038/srep25714. 27157914 PMC4860580

[ref32] Lu Y.; Yu Z.-D.; Liu Y.; Ding Y.-F.; Yang C.-Y.; Yao Z.-F.; Wang Z.-Y.; You H.-Y.; Cheng X.-F.; Tang B.; Wang J.-Y.; Pei J. The Critical Role of Dopant Cations in Electrical Conductivity and Thermoelectric Performance of N-Doped Polymers. J. Am. Chem. Soc. 2020, 142, 15340–15348. 10.1021/jacs.0c05699. 32786750

[ref33] Massetti M.; Jiao F.; Ferguson A. J.; Zhao D.; Wijeratne K.; Würger A.; Blackburn J. L.; Crispin X.; Fabiano S. Unconventional Thermoelectric Materials for Energy Harvesting and Sensing Applications. Chem. Rev. 2021, 121, 12465–12547. 10.1021/acs.chemrev.1c00218. 34702037

[ref34] Kim H. S.; Liu W.; Chen G.; Chu C.-W.; Ren Z. Relationship between Thermoelectric Figure of Merit and Energy Conversion Efficiency. Proc. Natl. Acad. Sci. U.S.A. 2015, 112, 8205–8210. 10.1073/pnas.1510231112. 26100905 PMC4500231

[ref35] Snyder G. J.; Snyder A. H. Figure of Merit ZT of a Thermoelectric Device Defined from Materials Properties. Energy Environ. Sci 2017, 10, 2280 10.1039/C7EE02007D.

[ref36] Zebarjadi M.; Esfarjani K.; Dresselhaus M. S.; Ren Z. F.; Chen G. Perspectives on Thermoelectrics: From Fundamentals to Device Applications. Energy Environ. Sci. 2012, 5, 5147–5162. 10.1039/C1EE02497C.

[ref37] Meng B.; Liu J.; Wang L. Recent Development of N-Type Thermoelectric Materials Based on Conjugated Polymers. Nano Mater. Sci. 2021, 3, 113–123. 10.1016/j.nanoms.2020.10.002.

[ref38] Paulsen B. D.; Tybrandt K.; Stavrinidou E.; Rivnay J. Organic Mixed Ionic–Electronic Conductors. Nat. Mater. 2020, 19, 13–26. 10.1038/s41563-019-0435-z. 31427743

[ref39] Tam T. L. D.; Xu J. Strategies and Concepts in N-Doped Conjugated Polymer Thermoelectrics. J. Mater. Chem. A 2021, 9, 5149–5163. 10.1039/D0TA12166E.

[ref40] Lu Y.; Wang J. Y.; Pei J. Strategies to Enhance the Conductivity of N-Type Polymer Thermoelectric Materials. Chem. Mater. 2019, 31, 6412–6423. 10.1021/acs.chemmater.9b01422.

[ref41] Savva A.; Cendra C.; Giugni A.; Torre B.; Surgailis J.; Ohayon D.; Giovannitti A.; McCulloch I.; Di Fabrizio E.; Salleo A.; Rivnay J.; Inal S. Influence of Water on the Performance of Organic Electrochemical Transistors. Chem. Mater. 2019, 31, 927–937. 10.1021/acs.chemmater.8b04335.

[ref42] Szumska A. A.; Maria I. P.; Flagg L. Q.; Savva A.; Surgailis J.; Paulsen B. D.; Moia D.; Chen X.; Griggs S.; Mefford J. T.; Rashid R. B.; Marks A.; Inal S.; Ginger D. S.; Giovannitti A.; Nelson J. Reversible Electrochemical Charging of N-Type Conjugated Polymer Electrodes in Aqueous Electrolytes. J. Am. Chem. Soc. 2021, 143, 14795–14805. 10.1021/jacs.1c06713. 34469688 PMC8447255

[ref43] Kiefer D.; Giovannitti A.; Sun H.; Biskup T.; Hofmann A.; Koopmans M.; Cendra C.; Weber S.; Anton Koster L. J.; Olsson E.; Rivnay J.; Fabiano S.; McCulloch I.; Müller C. Enhanced N-Doping Efficiency of a Naphthalenediimide-Based Copolymer through Polar Side Chains for Organic Thermoelectrics. ACS Energy Lett. 2018, 3, 278–285. 10.1021/acsenergylett.7b01146. 29457139 PMC5809982

[ref44] Liu J.; Ye G.; Potgieser H. G. O.; Koopmans M.; Sami S.; Nugraha M. I.; Villalva D. R.; Sun H.; Dong J.; Yang X.; Qiu X.; Yao C.; Portale G.; Fabiano S.; Anthopoulos T. D.; Baran D.; Havenith R. W. A.; Chiechi R. C.; Koster L. J. A. Amphipathic Side Chain of a Conjugated Polymer Optimizes Dopant Location toward Efficient N-Type Organic Thermoelectrics. Adv. Mater. 2021, 33, 2006694 10.1002/adma.202006694. PMC11468643 33306230

[ref45] Ye G.; Liu J.; Qiu X.; Stäter S.; Qiu L.; Liu Y.; Yang X.; Hildner R.; Jan Anton Koster L.; Chiechi R. C. Controlling N-Type Molecular Doping via Regiochemistry and Polarity of Pendant Groups on Low Band Gap Donor–Acceptor Copolymers. Macromolecules 2021, 54, 3886–3896. 10.1021/acs.macromol.1c00317. 34054145 PMC8154869

[ref46] Hallani R. K.; Paulsen B. D.; Petty A. J.; Sheelamanthula R.; Moser M.; Thorley K. J.; Sohn W.; Rashid R. B.; Savva A.; Moro S.; Parker J. P.; Drury O.; Alsufyani M.; Neophytou M.; Kosco J.; Inal S.; Costantini G.; Rivnay J.; McCulloch I. Regiochemistry-Driven Organic Electrochemical Transistor Performance Enhancement in Ethylene Glycol-Functionalized Polythiophenes. J. Am. Chem. Soc. 2021, 143, 11007–11018. 10.1021/jacs.1c03516. 34192463

[ref47] Moser M.; Savagian L. R.; Savva A.; Matta M.; Ponder J. F.; Hidalgo T. C.; Ohayon D.; Hallani R.; Reisjalali M.; Troisi A.; Wadsworth A.; Reynolds J. R.; Inal S.; McCulloch I. Ethylene Glycol-Based Side Chain Length Engineering in Polythiophenes and Its Impact on Organic Electrochemical Transistor Performance. Chem. Mater. 2020, 32, 6618–6628. 10.1021/acs.chemmater.0c02041.

[ref48] Moser M.; Savva A.; Thorley K.; Paulsen B. D.; Hidalgo T. C.; Ohayon D.; Chen H.; Giovannitti A.; Marks A.; Gasparini N.; Wadsworth A.; Rivnay J.; Inal S.; McCulloch I. Polaron Delocalization in Donor–Acceptor Polymers and Its Impact on Organic Electrochemical Transistor Performance. Angew. Chem. 2021, 133, 7856–7864. 10.1002/ange.202014078. 33259685

[ref49] Ohayon D.; Savva A.; Du W.; Paulsen B. D.; Uguz I.; Ashraf R. S.; Rivnay J.; McCulloch I.; Inal S. Influence of Side Chains on the N-Type Organic Electrochemical Transistor Performance. ACS Appl. Mater. Interfaces 2021, 13, 4253–4266. 10.1021/acsami.0c18599. 33439636

[ref50] Maria I. P.; Paulsen B. D.; Savva A.; Ohayon D.; Wu R.; Hallani R.; Basu A.; Du W.; Anthopoulos T. D.; Inal S.; Rivnay J.; McCulloch I.; Giovannitti A. The Effect of Alkyl Spacers on the Mixed Ionic-Electronic Conduction Properties of N-Type Polymers. Adv. Funct. Mater. 2021, 31, 2008718 10.1002/adfm.202008718.

[ref51] Krauss G.; Meichsner F.; Hochgesang A.; Mohanraj J.; Salehi S.; Schmode P.; Thelakkat M. Polydiketopyrrolopyrroles Carrying Ethylene Glycol Substituents as Efficient Mixed Ion-Electron Conductors for Biocompatible Organic Electrochemical Transistors. Adv. Funct. Mater. 2021, 31, 2010048 10.1002/adfm.202010048.

[ref52] Onwubiko A.; Yue W.; Jellett C.; Xiao M.; Chen H.-Y.; Ravva M. K.; Hanifi D. A.; Knall A.-C.; Purushothaman B.; Nikolka M.; Flores J.-C.; Salleo A.; Bredas J.-L.; Sirringhaus H.; Hayoz P.; Mcculloch I. Fused Electron Deficient Semiconducting Polymers for Air Stable Electron Transport. Nat. Commun. 2018, 9, 416 10.1038/s41467-018-02852-6. 29379022 PMC5789062

[ref53] Chen H.; Moser M.; Wang S.; Jellett C.; Thorley K.; Harrison G. T.; Jiao X.; Xiao M.; Purushothaman B.; Alsufyani M.; Bristow H.; De Wolf S.; Gasparini N.; Wadsworth A.; McNeill C. R.; Sirringhaus H.; Fabiano S.; McCulloch I. Acene Ring Size Optimization in Fused Lactam Polymers Enabling High N-Type Organic Thermoelectric Performance. J. Am. Chem. Soc. 2021, 143, 260–268. 10.1021/jacs.0c10365. 33350307

[ref54] Chen X.; Marks A.; Paulsen B. D.; Wu R.; Rashid R. B.; Chen H.; Alsufyani M.; Rivnay J.; McCulloch I. N -Type Rigid Semiconducting Polymers Bearing Oligo(Ethylene Glycol) Side Chains for High-Performance Organic Electrochemical Transistors. Angew. Chem., Int. Ed. 2021, 60, 9368–9373. 10.1002/anie.202013998. 33368944

[ref55] Ohayon D.; Inal S. Organic Bioelectronics: From Functional Materials to Next-Generation Devices and Power Sources. Adv. Mater. 2020, 1, 2001439 10.1002/adma.202001439. 32691880

[ref56] Stavrinidou E.; Leleux P.; Rajaona H.; Khodagholy D.; Rivnay J.; Lindau M.; Sanaur S.; Malliaras G. G. Direct Measurement of Ion Mobility in a Conducting Polymer. Adv. Mater. 2013, 25, 4488–4493. 10.1002/adma.201301240. 23784809

[ref57] Liang Y.; Brings F.; Maybeck V.; Ingebrandt S.; Wolfrum B.; Pich A.; Offenhäusser A.; Mayer D. Tuning Channel Architecture of Interdigitated Organic Electrochemical Transistors for Recording the Action Potentials of Electrogenic Cells. Adv. Funct. Mater. 2019, 29, 1902085 10.1002/adfm.201902085.

[ref58] Flagg L. Q.; Bischak C. G.; Onorato J. W.; Rashid R. B.; Luscombe C. K.; Ginger D. S. Polymer Crystallinity Controls Water Uptake in Glycol Side-Chain Polymer Organic Electrochemical Transistors. J. Am. Chem. Soc. 2019, 141, 4345–4354. 10.1021/jacs.8b12640. 30779568

[ref59] Surgailis J.; Savva A.; Druet V.; Paulsen B. D.; Wu R.; Hamidi-Sakr A.; Ohayon D.; Nikiforidis G.; Chen X.; McCulloch I.; Rivnay J.; Inal S. Mixed Conduction in an N-Type Organic Semiconductor in the Absence of Hydrophilic Side-Chains. Adv. Funct. Mater. 2021, 31, 2010165 10.1002/adfm.202010165.

[ref60] Feng K.; Shan W.; Ma S.; Wu Z.; Chen J.; Guo H.; Liu B.; Wang J.; Li B.; Woo H. Y.; Fabiano S.; Huang W.; Guo X. Fused Bithiophene Imide Dimer-Based N-Type Polymers for High-Performance Organic Electrochemical Transistors. Angew. Chem., Int. Ed. 2021, 60, 24198–24205. 10.1002/anie.202109281. 34467624

[ref61] DeLongchamp D. M.; Vogel B. M.; Jung Y.; Gurau M. C.; Richter C. A.; Kirillov O. A.; Obrzut J.; Fischer D. A.; Sambasivan S.; Richter L. J.; Lin E. K. Variations in Semiconducting Polymer Microstructure and Hole Mobility with Spin-Coating Speed. Chem. Mater. 2005, 17, 5610–5612. 10.1021/cm0513637.

[ref62] Shen H.; Abtahi A.; Lussem B.; Boudouris B. W.; Mei J. Device Engineering in Organic Electrochemical Transistors toward Multifunctional Applications. ACS Appl. Electron. Mater. 2021, 3, 2434–2448. 10.1021/acsaelm.1c00312.

[ref63] Shi K.; Zhang F.; Di C.-A. A.; Yan T.-W. W.; Zou Y.; Zhou X.; Zhu D.; Wang J.-Y. Y.; Pei J. Toward High Performance N-Type Thermoelectric Materials by Rational Modification of BDPPV Backbones. J. Am. Chem. Soc. 2015, 137, 6979–6982. 10.1021/jacs.5b00945. 25997085

[ref64] Zhao X.; Madan D.; Cheng Y.; Zhou J.; Li H.; Thon S. M.; Bragg A. E.; DeCoster M. E.; Hopkins P. E.; Katz H. E. High Conductivity and Electron-Transfer Validation in an n-Type Fluoride-Anion-Doped Polymer for Thermoelectrics in Air. Adv. Mater. 2017, 29, 1606928 10.1002/adma.201606928. 28707300

[ref65] Lu Y.; Yu Z.; Zhang R.; Yao Z.; You H.; Jiang L.; Un H.; Dong B.; Xiong M.; Wang J.; Pei J. Rigid Coplanar Polymers for Stable N-Type Polymer Thermoelectrics. Angew. Chem., Int. Ed. 2019, 58, 11390–11394. 10.1002/anie.201905835. 31187584

[ref66] Wei P.; Oh J. H.; Dong G.; Bao Z. Use of a 1 H -Benzoimidazole Derivative as an n -Type Dopant and To Enable Air-Stable Solution-Processed n -Channel Organic Thin-Film Transistors. J. Am. Chem. Soc. 2010, 132, 8852–8853. 10.1021/ja103173m. 20552967

[ref67] Wang S.; Sun H.; Ail U.; Vagin M.; Persson P. O. Å. Å.; Andreasen J. W.; Thiel W.; Berggren M.; Crispin X.; Fazzi D.; Fabiano S. Thermoelectric Properties of Solution-Processed n-Doped Ladder-Type Conducting Polymers. Adv. Mater. 2016, 28, 10764–10771. 10.1002/adma.201603731. 27787927

[ref68] Culebras M.; Uriol B.; Gómez C. M.; Cantarero A. Controlling the Thermoelectric Properties of Polymers: Application to PEDOT and Polypyrrole. Phys. Chem. Chem. Phys. 2015, 17, 15140–15145. 10.1039/C5CP01940K. 25990660

[ref69] Dong C.; Deng S.; Meng B.; Liu J.; Wang L. A Distannylated Monomer of a Strong Electron-Accepting Organoboron Building Block: Enabling Acceptor–Acceptor-Type Conjugated Polymers for N-Type Thermoelectric Applications. Angew. Chem., Int. Ed. 2021, 60, 16184–16190. 10.1002/anie.202105127. 33956396

