# Supplementary material for: Synthetic Nuances to Maximize n-Type Organic Electrochemical Transistor and Thermoelectric Performance in Fused Lactam Polymers
Source: J Am Chem Soc. 2022 Mar 8;144(10):4642–56. doi: 10.1021/jacs.2c00735 (PMC9084553; doi:10.1021/jacs.2c00735)
Supplement: Supplementary file 1 — ja2c00735_si_001.pdf [file ja2c00735_si_001.pdf]

SUPPORTING INFORMATION

Synthetic nuances to maximize n-type organic electrochemical transistor and thermoelectric performance in fused lactam polymers

Adam Marks [a*‡†], Xingxing Chen [b*‡], Ruiheng Wu [c], Reem B. Rashid [d], Wenlong Jin [e], Bryan D. Paulsen [d], Maximilian Moser [a], Xudong Ji [d], Sophie Griggs [a], Dilara Meli [f], Xiaocui Wu [g], Helen Bristow [a], Joseph Strzalka [h], Nicola Gasparini [i], Giovanni Costantini [g], Simone Fabiano [e], Jonathan Rivnay [d] and Iain McCulloch [a*]

[a] Department of Chemistry, University of Oxford, Oxford OX1 3TA, UK

[b] KAUST Solar Center (KSC), King Abdullah University of Science and Technology (KAUST), Thuwal 23955-6900, Saudi Arabia

[c] Department of Chemistry, Northwestern University, Evanston, IL, 60208, USA

[d] Department of Biomedical Engineering, Northwestern University, Evanston, IL, 60208, USA

[e] Laboratory of Organic Electronics, Department of Science and Technology, Linköping University, Norrköping SE-60174, Sweden

[f] Department of Material Science, Northwestern University, Evanston, IL, 60208, USA

[g] Department of Chemistry, University of Warwick, Coventry CV4 7AL, UK

[h] X-Ray Science Division, Argonne National Laboratory, Lemont, IL 60439, USA

[i] Department of Chemistry and Centre for Processable Electronics, Imperial College London, London W12 0BZ, UK.

*Email: am21@stanford.edu , xingxing.chen@kaust.edu.sa and iain.mcculloch@chem.ox.ac.uk

†Department of Materials Science and Engineering, Stanford University, Stanford, CA, USA.

‡A. Marks and X.Chen contributed equally to this manuscript.

Table of Contents

1. EXPERIMENTAL SECTION 3

1.1. General methods: 3

1.2. GIWAXS sample preparation and measurements: 4

1.3. Spectroelectrochemical sample preparation and measurements: 4

1.4. OECT device fabrication and testing: 5

1.5. Thermoelectric performance characterization: 5

2. SYNTHESIS & CHARACTERIZATION......6
2.1. General synthesis:......6
2.2. Monomer synthesis:......6
2.2.1. General procedure for step (i)......7
2.2.2. General procedure for step (ii)......8
2.2.3. General procedure for step (iii)......10
2.2.4. Synthesis of C_2N monomer:......18
2.3. Polymer synthesis:......21
2.3.1. General polymerization procedure (iv):......22
2.3.2. p(g_7NC_2N):......23
2.3.3. p(g_7NC_4N):......24
2.3.4. p(g_7NC_6N):......24
2.3.5. p(g_7NC_8N):......26
2.3.6. p($g_7NC_{10}N$):......27
2.3.7. p($g_7NC_{16}N$):......28
3. POLYMER PROPERTIES......29
3.1. UV-Vis Absorption Spectroscopy......29
3.2. Cyclic Voltammetry......30
3.3. Spectroelectrochemistry......31
4. ORGANIC ELECTROCHEMICAL TRANSISTOR DATA......35
4.1. Electrochemical Impedance Spectroscopy......35
4.2. OECT figures of merit Summary......36
4.3. Transfer, Output & Stability Curves......37
4.3.1. p(g_7NC_2N)......37
4.3.2. p(g_7NC_4N)......37
4.3.3. p(g_7NC_6N)......38
4.3.4. p(g_7NC_8N)......39
4.3.5. p($g_7NC_{10}N$)......40
4.3.6. p($g_7NC_{16}N$)......41
5. NEAT GRAZING-INCIDENCE WIDE-ANGLE X-RAY SCATTERING DATA..41
6. STM IMAGING......43
7. THERMOELECTRIC MEASUREMENTS......44
8. DOPED GRAZING-INCIDENCE WIDE-ANGLE X-RAY SCATTERING DATA47
9. REFERENCES......49

1. EXPERIMENTAL SECTION

1.1. General methods:

All reactants and reagents were purchased from Sigma-Aldrich (UK) and were used without further purification. Solvents used were purified by standard methods. All reactions, unless otherwise noted, were carried out on a standard Schlenk line technique under N_2 gas using oven-dried glassware. NMR spectra were acquired with B400 Bruker Avance III 400 MHz spectrometer, using TMS as an internal standard (0.00 ppm). Mass measurements were acquired with a Micromass MALDI-ToF using matrix-assisted laser desorption ionization (MALDI) techniques on linear acquisition operation mode and a positive voltage polarity, using trans-2-[3-(4-tert-Butylphenyl)-2-methyl-2-propenylidene]-malononitrile (DCTB) as the matrix. Ultraviolet-visible (UV-Vis) spectra were recorded on a Shimadzu UV-1800 spectrophotometer from solutions of $CHCl_3$. Cyclic voltammetry (CV) was recorded using an PGSTAT101 with a standard working (ITO glass slide or polymer coated platinum disk electrode), reference ((Ag/Ag+) calibrated against ferrocene (Fc/Fc^+)) and counter electrode (platinum mesh) setup. Measurements were conducted in degassed 0.1 M tetra-n-butylammonium hexafluorophosphate (NBu_4PF_6) anhydrous acetonitrile solution (organic) or a 0.1 M NaCl aqueous solution as the supporting electrolyte at a scan rate of 100 mV s^{-1}. Prior to organic CV measurements in acetonitrile, all glassware was oven dried at 100 °C, the cell was purged with nitrogen during the experiment to reduce oxygen contamination. Ionization potentials (IP) were obtained using the equation IP = (E_{ox} – E_{Fc/Fc^+} + 4.8V) where appropriate electron affinities (EA) were calculated from the onset of the first reduction using equation EA = -(4.8 + E_{red} - E_{Fc/Fc^+}). Gel Permeation Chromatography (GPC) was performed with an Agilent Technologies 1200 Series with refractive index detection, chloroform solvent, 40 °C column temperature and polystyrene standard.

1.2. GIWAXS sample preparation and measurements:

2D GIWAXS patterns were collected from films spun at 600 rpm for 60 seconds, from polymer solutions (5 mg ml^{-1} in chloroform) on polished Si wafer substrates (University Wafer). Scattering was carried out at the Advanced Photon Source at Argonne National Laboratory on beam line 8-ID-E at room temperature under vacuum with 10.92 keV (λ = 1.135 Å) synchrotron radiation, with a 0.14° incident angle, and measured with a Pilatus 1M hybrid pixel array detector during 10 second exposures. All data analysis was carried out with GIXSGUI Matlab toolbox.[1]

1.3. Spectroelectrochemical sample preparation and measurements:

To prepare spectroelectrochemical samples, polymers were spin-coated from a 5 mg/mL chloroform solution at 600 rpm for 60 s on pieces of 1 × 3 cm2 Indium tin oxide (ITO) coated glasses (15-20 ohm sq^{-1}) (University Wafer) that prior to spinning had been successively sonicated in acetone and isopropyl alcohol, and cleaned with UV-ozone exposure. Spectroelectrochemistry measurements of the polymer coated ITO electrodes were carried out in 100 mM aqueous NaCl in a PMMA cuvette with an Ag/AgCl pellet (Warner Instruments) reference/counter electrode. Potential control and current measurement were carried out with a potentiostat (Ivium). Simultaneous absorption spectroscopy was recorded with a halogen white light source (Ocean Optics, DH-2000-BAL) and an optical fiber light path split to separate UV-visible (Ocean Optics, FLAME-S) and near-infrared (Ocean Optics, NQ512) spectrometers, with 30 ms and 120 ms integration times, respectively. Electrochemical and spectroscopic data were recorded with Iviumsoft and OceanView software, respectively. EA (LUMO level) was estimated from the onset of reduction. The Ag/AgCl pellet electrode used was calibrated against a 3M NaCl Ag/AgCl reference electrode known to be +0.206 V vs NHE, with NHE lying at -4.44 eV on the vacuum scale.

1.4. OECT device fabrication and testing:

OECTs test chips were prepared following microfabrication techniques previously reported.[2] OECT channels were fabricated by drop casting from chloroform (5 mg/mL) onto OECT test chips at room temperature, followed by patterning via peeling a sacrificial parylene layer, and a rinse in deionized water. OECTs were gated with aqueous 100 mM NaCl using an Ag/AgCl pellet as the faradaic gate electrode.[3] Electrical characterization (output, transfer, and pulsed stability) of the OECTs were carried out using NI source-measure units controlled by custom LabView code. Capacitance of individual drop cast channels was determined via EIS using a Metrohm potentiostat with frequency response analyzer with an Ag/AgCl pellet functioning as a combined reference and counter electrode.[3] μC* was calculated from the slope of the transfer curves, which is the gate transconductance ($gm = \partial I_d/\partial V_g$), using the relation $gm = Wd/L \times \mu C^* \times (V_t - V_g)$.

1.5. Thermoelectric performance characterization:

All processes related to film deposition and doping were carried out in the glovebox with oxygen and water level below 1 ppm. Glass substrates were sequentially sonicated in deionized water, acetone, and isopropanol for 5 minutes, followed by blow drying with nitrogen gas. Organic semiconductors (C2~16) were dissolved in chloroform at a concentration of 5 mg mL^{-1} and stirred for at least 5 hours at room temperature. Different weight percentages of dopant (N-DMBI dissolved in chloroform) were then added to the polymer solution and stirred for 30 minutes to guarantee sufficient mixing, then spin-coated on the substrate at 1000 rpm and then annealed at 150 °C on a hotplate for 8 hours.[4,5] The samples for TDAE vapor doping were directly spin-coated from neat organic semiconductor solutions at 1000 rpm.

Both electrical conductivity (σ) and Seebeck coefficient (S) were measured with a Keithley 4200 SCS semiconductor parameter analyzer on the same sample deposited on a glass substrate. Parallel electrodes (Cr/Au, 5/30 nm) were preprepared by photolithography with length, width and distance between the two electrodes of 10,000 μm, 50 μm and 500 μm, respectively.

Electrical conductivity was measured by standard four-probe method. Seebeck coefficient was obtained by fitting the thermal voltage difference (ΔV) versus temperature difference (ΔT) between hot and cold sides, where temperature difference was generated by two Peltier modules. For TDAE vapor doping, the neat polymer films were exposed to TDAE vapor in a vial (20 mL in volume filled with 1 mL liquid TDAE) for a specified time, then the device was taken out of the vial and electrical conductivity and Seebeck coefficients were measured.[6]

2. SYNTHESIS & CHARACTERIZATION

2.1. General synthesis:

Monomer **g_7N** was synthesized following previously reported methods.[7]

2.2. Monomer synthesis:

Each monomer was synthesized following an identical synthetic route apart from **C_2N** which was synthesized following an alternative route (**Figure S15**) due to the limited solubility hindering the final C-H activated ring closure step (**Figure S1**).

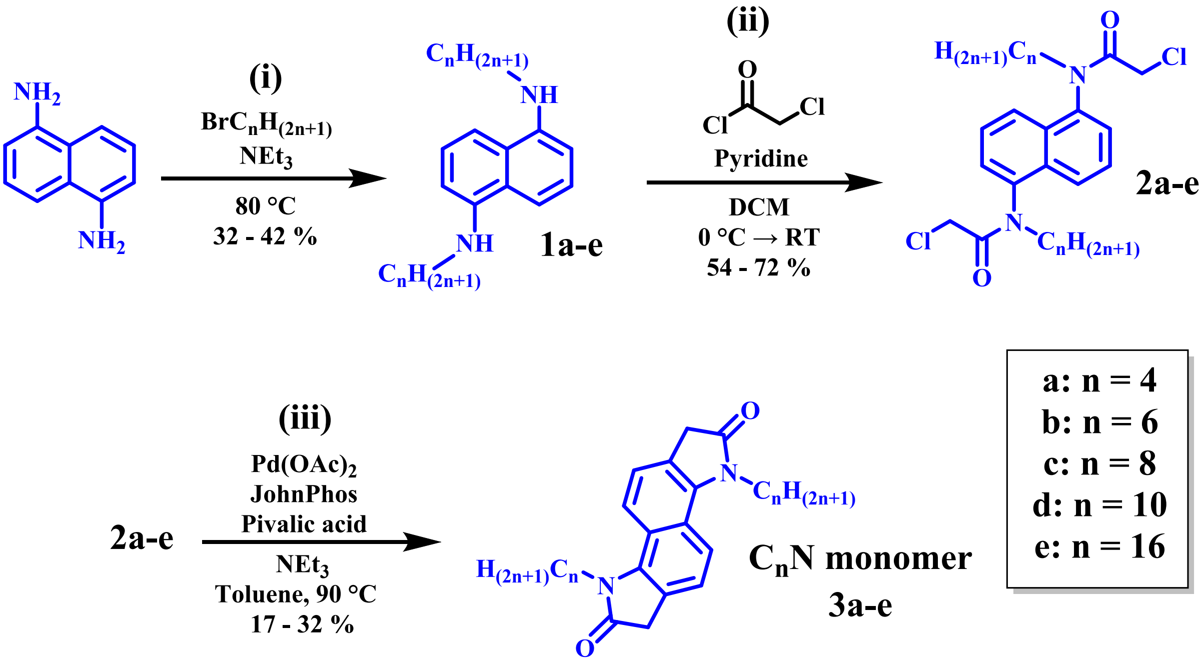

Figure S1. Full reaction scheme to afford alkylated bis-oxindole monomers **3a-e**.

2.2.1. General procedure for step (i)

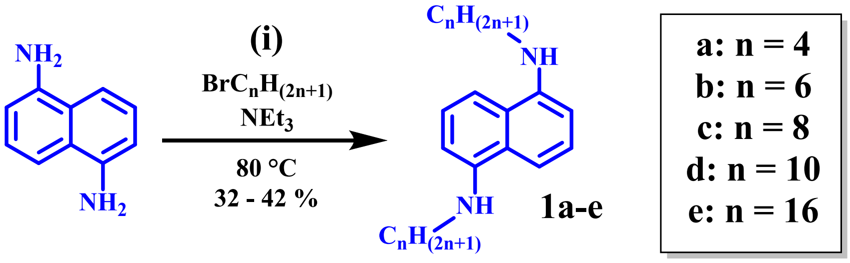

Figure S2. General conditions for the N-alkylation of 1,5-diaminonaphthalene to afford **1a-e**.

1,5-diaminonaphthalene (1.0 eq.) was suspended in 300 mL of ethanol, prior to the dropwise addition of triethylamine (3.07 eq.). The solution was heated to reflux and stirred under nitrogen for 30 minutes. The corresponding 1-bromoalkyl (2.74 eq.) was added dropwise and the solution was stirred, at reflux, overnight. Upon cooling to room temperature, the precipitate was filtered and washed with toluene (300 mL), the off-white solid was subsequently purified by hot filtration from a boiling hexane solution to afford a candyfloss pink crystalline solid.

***N^1,N^5*-dibutylnaphthalene-1,5-diamine (1a):** was synthesized following the general procedure for step (i), 10.56 g, 39.05 mmol, 36 %. ^{1}H NMR (400 MHz, $CDCl_3$) δ 7.31 (t, *J* = 8.0 Hz, 2H), 7.15 (d, *J* = 8.4 Hz, 2H), 6.61 (d, *J* = 7.5 Hz, 2H), 4.31 (s, 2H), 3.27 (t, *J* = 7.1 Hz, 4H), 1.76 (p, *J* = 7.2 Hz, 4H), 1.54 (dt, *J* = 14.8, 7.4 Hz, 4H), 1.01 (t, *J* = 7.4 Hz, 6H). $^{13}C\{^1H\}$ NMR (101 MHz, $CDCl_3$) δ 144.4, 125.6, 124.0, 108.6, 104.4, 44.1, 31.7, 20.7, 14.1.

***N^1,N^5*-dihexylnaphthalene-1,5-diamine (1b):** was synthesized following the general procedure for step (i), 6.45 g, 19.75 mmol, 42 %. ^{1}H NMR (400 MHz, $CDCl_3$) δ 7.90 (dt, *J* = 8.0, 1.2 Hz, 2H), 7.64 (dd, *J* = 8.5, 7.3 Hz, 2H), 7.50 (dd, *J* = 7.2, 1.0 Hz, 2H), 4.30 (ddt, *J* = 13.2, 10.1, 5.7 Hz, 2H), 3.75 (dd, *J* = 13.3, 6.9 Hz, 2H), 3.65 (dd, *J* = 13.3, 6.7 Hz, 2H), 3.27 (tdd, *J* = 12.7, 10.1, 5.1 Hz, 2H), 1.63 – 1.48 (m, 2H), 1.32 – 1.21 (m, 12H), 0.85 (t, *J* = 1.7 Hz, 6H). $^{13}C\{^1H\}$ NMR (101 MHz, $CDCl_3$) δ 166.6, 166.6, 138.3, 131.9, 128.1, 128.0, 127.7, 127.7, 123.9, 123.8, 50.3, 50.2, 42.2, 42.1, 31.7, 31.6, 28.1, 28.0, 26.6, 22.8, 22.7, 14.2, 14.1.

***N^1,N^5*-dioctylnaphthalene-1,5-diamine (1c):** was synthesized following the general procedure for step (i), 7.99 g, 20.88 mmol, 32 %. ^{1}H NMR (400 MHz, $CDCl_3$) δ 7.30 (dd, *J* = 8.4, 7.5 Hz,

2H), 7.14 (d, *J* = 8.4 Hz, 2H), 6.60 (d, *J* = 7.5 Hz, 2H), 4.31 (s, 2H), 3.25 (t, *J* = 7.1 Hz, 4H), 1.76 (p, *J* = 7.2 Hz, 4H), 1.48 (tt, *J* = 9.2, 5.9 Hz, 4H), 1.43 – 1.23 (m, 16H), 0.94 – 0.85 (m, 6H). $^{13}C\{^1H\}$ NMR (101 MHz, $CDCl_3$) δ 144.4, 125.6, 124.0, 108.6, 104.4, 77.4, 44.4, 32.0, 29.6, 29.4, 27.5, 22.8, 14.3.

***N¹*,*N⁵*-didecylnaphthalene-1,5-diamine (1d):** was synthesized following the general procedure for step (i), 18.44 g, 42.03 mmol, 33 %. ^1H NMR (400 MHz, $CDCl_3$) δ 7.31 (t, *J* = 8.0 Hz, 2H), 7.15 (d, *J* = 8.4 Hz, 2H), 6.60 (d, *J* = 7.6 Hz, 2H), 4.31 (s, 2H), 3.25 (t, *J* = 7.1 Hz, 4H), 1.76 (p, *J* = 7.2 Hz, 4H), 1.58 – 1.47 (m, 2H), 1.50 – 1.43 (m, 2H), 1.43 – 1.36 (m, 2H), 1.36 – 1.32 (m, 4H), 1.38 – 1.25 (m, 18H), 0.89 (t, 6H). $^{13}C\{^1H\}$ NMR (101 MHz, $CDCl_3$) δ 144.4, 125.6, 124.0, 108.6, 104.4, 44.4, 32.1, 29.8, 29.7, 29.7, 29.6, 29.5, 27.5, 22.8, 14.3.

***N¹*,*N⁵*-dihexadecylnaphthalene-1,5-diamine (1e):** was synthesized following the general procedure for step (i), 12.42 g, 20.44 mmol, 39 %. ^1H NMR (400 MHz, $CDCl_3$) δ 7.31 (dd, J = 8.5, 7.3 Hz, 2H), 7.25 (d, J = 8.6 Hz, 2H), 6.62 (d, J = 7.3 Hz, 2H), 4.88 (br, 2H), 3.86 (t, J = 5.16 Hz, 4H), 3.74 – 3.63 (m, 20H), 3.56 – 3.52 (m, 4H), 3.44 (t, J = 5.2 Hz, 4H), 3.36 (s, 6H). $^{13}C\{^1H\}$ NMR (101 MHz, $CDCl_3$): δ 144.0, 125.4, 124.4, 109.5, 104.9, 71.9, 70.6, 70.3, 69.4, 59.1, 43.8, 31.7, 31.6, 28.1, 28.0, 26.6, 22.8, 22.7, 14.2, 14.1.

2.2.2. General procedure for step (ii)

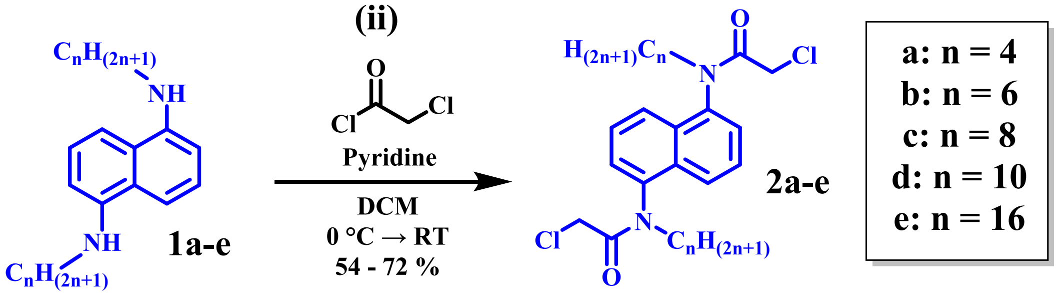

Figure S3. General conditions for the chloroacetylation of N-alkylated compounds **1a-e** to afford products **2a-e**.

*N*¹,*N*⁵-dialkylnaphthalene-1,5-diamine (1.0 eq.) was suspended in 30 mL dry DCM, cooled to 0 °C under a nitrogen atmosphere. Pyridine (3.0 eq.) and 2-chloroacetyl chloride (2.5 eq.) was added dropwise, then warmed to room temperature over 90 minutes. The mixture was poured

into water, washed with brine and saturated sodium bicarbonate solution, extracted into DCM. Once separated the organic phase was dried over $MgSO_4$, solvent removed under reduced pressure. The brown residue was purified using column chromatography, on silica, using DCM:acetone (99:1) as the eluent system to afford an off-white solid.

***N,N′*-(naphthalene-1,5-diyl)bis(N-butyl-2-chloroacetamide) (2a):** was synthesized following the general procedure for step (ii), 6.29 g, 14.86 mmol, 67 %. ^{1}H NMR (400 MHz, $CDCl_3$) δ 7.90 (dt, *J* = 8.0, 1.2 Hz, 2H), 7.64 (ddd, *J* = 8.2, 7.2, 0.8 Hz, 2H), 7.50 (dd, *J* = 7.2, 1.0 Hz, 2H), 4.31 (dddd, *J* = 13.2, 10.0, 6.1, 4.9 Hz, 2H), 3.75 (dd, *J* = 13.3, 6.4 Hz, 2H), 3.65 (dd, *J* = 13.2, 6.3 Hz, 2H), 3.27 (dddd, *J* = 13.2, 11.6, 10.0, 5.1 Hz, 2H), 1.76 – 1.45 (m, 4H), 1.45 – 1.22 (m, 4H), 0.90 (t, *J* = 4.5 Hz, 6H). $^{13}C\{^1H\}$ NMR (101 MHz, $CDCl_3$) δ 166.6, 166.6, 138.2, 131.9, 128.1, 128.1, 127.7, 127.7, 123.8, 123.8, 77.4, 50.0, 49.9, 42.2, 42.1, 30.1, 30.1, 20.2, 13.9.

***N,N′*-(naphthalene-1,5-diyl)bis(2-chloro-N-hexylacetamide) (2b):** was synthesized following the general procedure for step (ii), 6.41 g, 13.37 mmol, 62 %. ^{1}H NMR (400 MHz, $CDCl_3$) δ 7.94 – 7.85 (m, 2H), 7.65 (dd, *J* = 8.5, 7.3 Hz, 2H), 7.50 (dd, *J* = 7.2, 1.0 Hz, 2H), 4.31 (ddt, *J* = 13.2, 10.1, 5.7 Hz, 2H), 3.75 (dd, *J* = 13.3, 6.8 Hz, 2H), 3.65 (dd, *J* = 13.2, 6.6 Hz, 2H), 3.27 (tdd, *J* = 12.8, 10.1, 5.1 Hz, 2H), 1.74 – 1.50 (m, 2H), 1.41 – 1.22 (m, 14H), 0.85 (t, *J* = 1.8 Hz, 6H). $^{13}C\{^1H\}$ NMR (101 MHz, $CDCl_3$) δ 166.6, 138.3, 131.9, 128.1, 127.7, 127.7, 123.9, 123.8, 50.3, 50.2, 42.2, 42.1, 31.6, 28.1, 28.0, 26.6, 22.7, 14.1.

***N,N′*-(naphthalene-1,5-diyl)bis(2-chloro-N-octylacetamide) (2c):** was synthesized following the general procedure for step (ii), 3.76 g, 7.02 mmol, 54 %. ^{1}H NMR (400 MHz, $CDCl_3$) δ 7.90 (dt, *J* = 8.0, 1.2 Hz, 2H), 7.65 (dd, *J* = 8.3, 7.1 Hz, 2H), 7.50 (dd, *J* = 7.2, 1.0 Hz, 2H), 4.30 (ddt, *J* = 13.2, 10.1, 5.7 Hz, 2H), 3.75 (dd, *J* = 13.2, 7.0 Hz, 2H), 3.65 (dd, *J* = 13.3, 6.8 Hz, 2H), 3.27 (tdd, *J* = 13.0, 10.1, 5.1 Hz, 2H), 1.39 – 1.20 (m, 24H), 0.85 (t, *J* = 4.5 Hz, 6H).

$^{13}C\{^{1}H\}$ NMR (101 MHz, $CDCl_3$) δ 166.6, 166.6, 138.3, 131.9, 128.1, 127.7, 127.7, 123.9, 123.8, 50.3, 50.3, 42.2, 42.1, 31.9, 29.4, 29.3, 28.1, 28.1, 26.9, 22.7, 14.2.

***N,N′*-(naphthalene-1,5-diyl)bis(2-chloro-N-decylacetamide) (2d):** was synthesized following the general procedure for step (ii), 7.45 g, 12.59 mmol, 69 %. ^{1}H NMR (400 MHz, $CDCl_3$) δ 7.90 (d, *J* = 8.5 Hz, 2H), 7.64 (t, *J* = 7.9 Hz, 2H), 7.50 (d, *J* = 7.2 Hz, 2H), 4.30 (ddd, *J* = 12.7, 10.2, 5.7 Hz, 2H), 3.75 (dd, *J* = 13.3, 6.9 Hz, 2H), 3.65 (dd, *J* = 13.3, 6.7 Hz, 2H), 3.26 (dtd, *J* = 18.0, 11.6, 5.0 Hz, 2H), 1.74 – 1.60 (m, 2H), 1.55 (ddq, *J* = 15.3, 10.3, 5.4 Hz, 2H), 1.31 – 1.20 (m, 28H), 0.86 (t, *J* = 6.7 Hz, 6H). $^{13}C\{^{1}H\}$ NMR (101 MHz, $CDCl_3$) δ 166.6, 166.5, 138.3, 131.9, 128.1, 128.0, 127.7, 127.6, 123.9, 123.8, 77.4, 50.3, 50.2, 42.2, 42.1, 31.9, 29.7, 29.6, 29.5, 29.4, 28.1, 28.0, 26.9, 22.8, 14.2.

***N,N′*-(naphthalene-1,5-diyl)bis(2-chloro-N-hexadecylacetamide) (2e):** was synthesized following the general procedure for step (ii), 8.15 g, 10.73 mmol, 72 %. ^{1}H NMR (400 MHz, $CDCl_3$) δ 7.94 – 7.87 (m, 2H), 7.67 – 7.62 (m, 2H), 7.50 (dd, *J* = 7.2, 1.0 Hz, 2H), 4.40 – 4.22 (m, 2H), 3.79 – 3.59 (m, 4H), 3.36 – 3.19 (m, 2H), 1.62 – 1.50 (m, 2H), 1.25 (t, *J* = 8.3 Hz, 54H), 0.87 (t, *J* = 6.9 Hz, 6H). $^{13}C\{^{1}H\}$ NMR (101 MHz, $CDCl_3$) δ 166.6, 138.3, 128.1, 127.7, 123.9, 50.4, 42.1, 32.1, 29.8, 29.8, 29.7, 29.7, 29.5, 28.1, 27.0, 22.8, 14.3.

2.2.3. General procedure for step (iii)

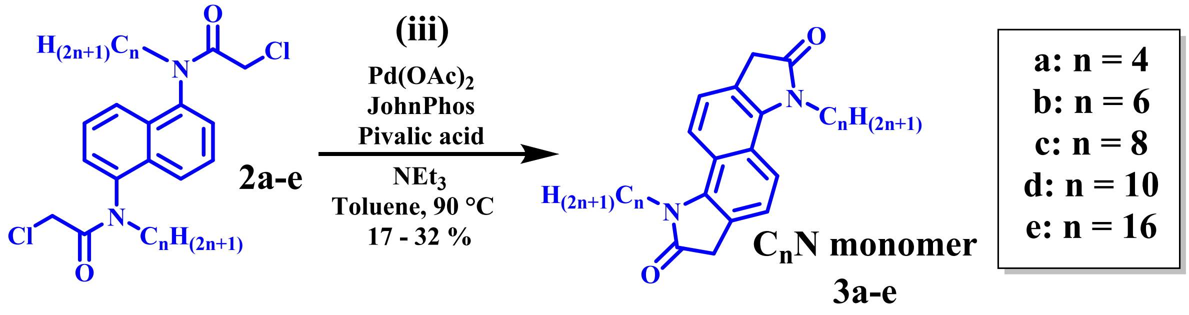

Figure S4. General conditions for the C-H activated ring closure to afford **C_nN** monomers **3a-e**.

An oven dried 10 mL microwave vial was charged with *N,N′*-(naphthalene-1,5-diyl)bis(2-chloro-N-alkylacetamide) (1.0 eq.), $Pd(OAc)_2$ (0.12 eq.), JohnPhos (0.24 eq.) and pivalic acid (0.61 eq.). The cap was sealed and degassed with nitrogen for 10 minutes, prior to the addition

of 6 mL anhydrous toluene. Triethylamine (2.94 eq.) was slowly added, the reaction vial was placed into a pre-heated oil bath (90 °C) and stirred rapidly overnight. The mixture was cooled to room temperature, passed through a short pad of silica, eluting with DCM, solvent removed under reduced pressure. The crude brown residue was purified via column chromatography on silica using ethyl acetate:hexane (1:5) as the eluent, recrystallisation from hot hexane afforded a beige solid.

3,8-dibutyl-1,3,6,8-tetrahydroindolo[7,6-*g*]indole-2,7-dione (3a) C_4N monomer: was synthesized following the general procedure for step (iii), 146 mg, 0.45 mmol, 18 %. ^1H NMR (400 MHz, $CDCl_3$) δ 7.94 (d, J = 8.6 Hz, 2H), 7.40 (d, J = 8.6 Hz, 2H), 4.30 – 4.22 (m, 4H), 3.67 (s, 4H), 1.87 – 1.75 (m, 4H), 1.48 (h, J = 7.4 Hz, 4H), 0.99 (t, J = 7.4 Hz, 6H). $^{13}C\{^1H\}$ NMR (101 MHz, $CDCl_3$) δ 176.7, 140.75, 122.1, 121.9, 120.5, 115.9, 42.7, 36.2, 31.6, 20.2, 13.9. MALDI-TOF: $[M+H]^+$ calc. ($C_{22}H_{26}N_2O_2$): 350.20 found: 351.47.

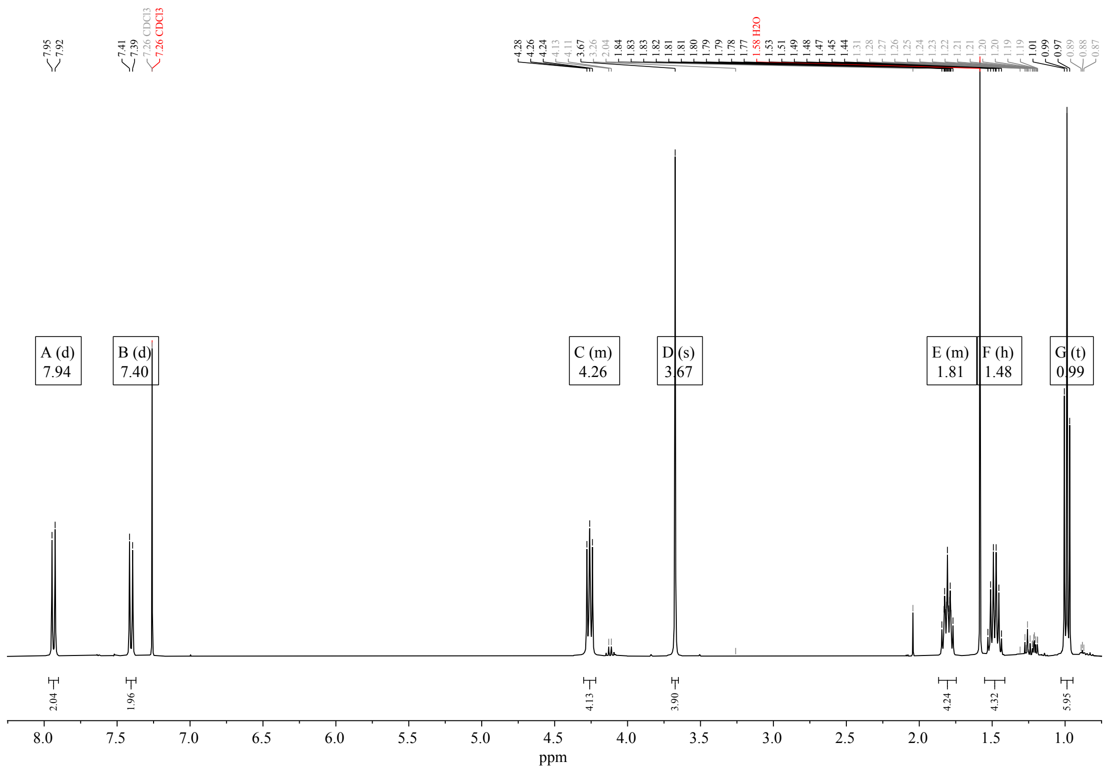

Figure S5. ^1H NMR spectrum of compound **(3a) C_4N** monomer.

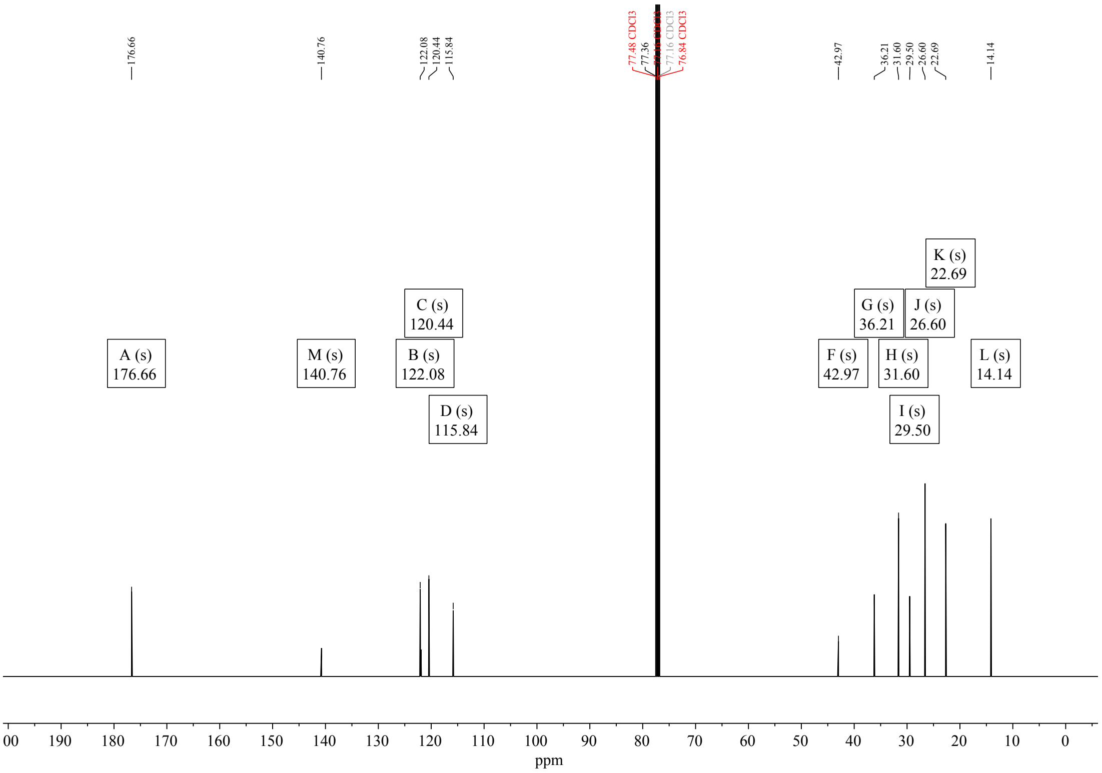

Figure S6. ^{13}C NMR spectrum of compound **(3a) C$_4$N** monomer.

3,8-dihexyl-1,3,6,8-tetrahydroindolo[7,6-*g*]indole-2,7-dione (3b) C$_6$N monomer: was synthesized following the general procedure for step (iii), 141 mg, 0.35 mmol, 17 %. ^1H NMR (400 MHz, $CDCl_3$) δ 7.93 (d, J = 8.6 Hz, 2H), 7.40 (d, J = 8.6 Hz, 2H), 4.28 – 4.20 (m, 4H), 3.67 (s, 4H), 1.87 – 1.75 (m, 4H), 1.52 – 1.41 (m, 4H), 1.38 – 1.14 (m, 8H), 0.89 (t, J = 6.9 Hz, 6H). $^{13}C\{^1H\}$ NMR (101 MHz, $CDCl_3$) δ 176.5, 140.6, 121.9, 121.8, 120.3, 115.7, 42.8, 36.1, 31.5, 29.4, 26.5, 22.6, 14.0. MALDI-TOF: $[M+H]^+$ calc. ($C_{26}H_{34}N_2O_2$): 406.26 found: 407.27.

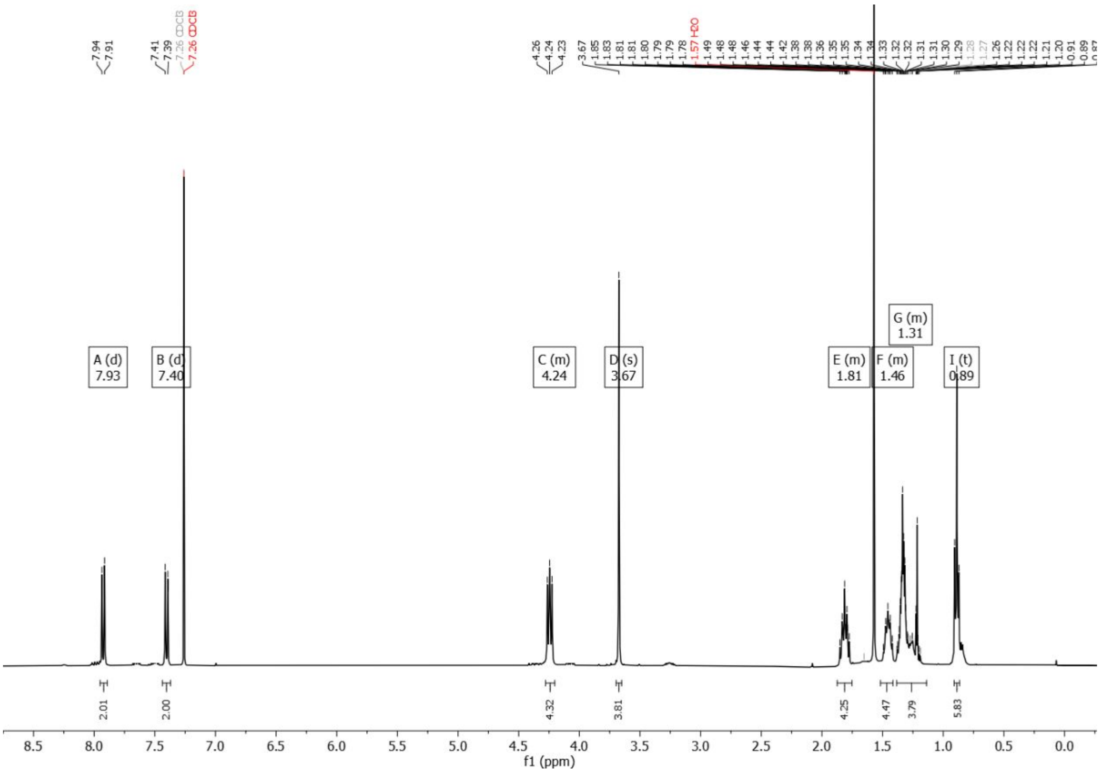

Figure S7. ^{1}H NMR spectrum of compound **(3b) C$_6$N** monomer.

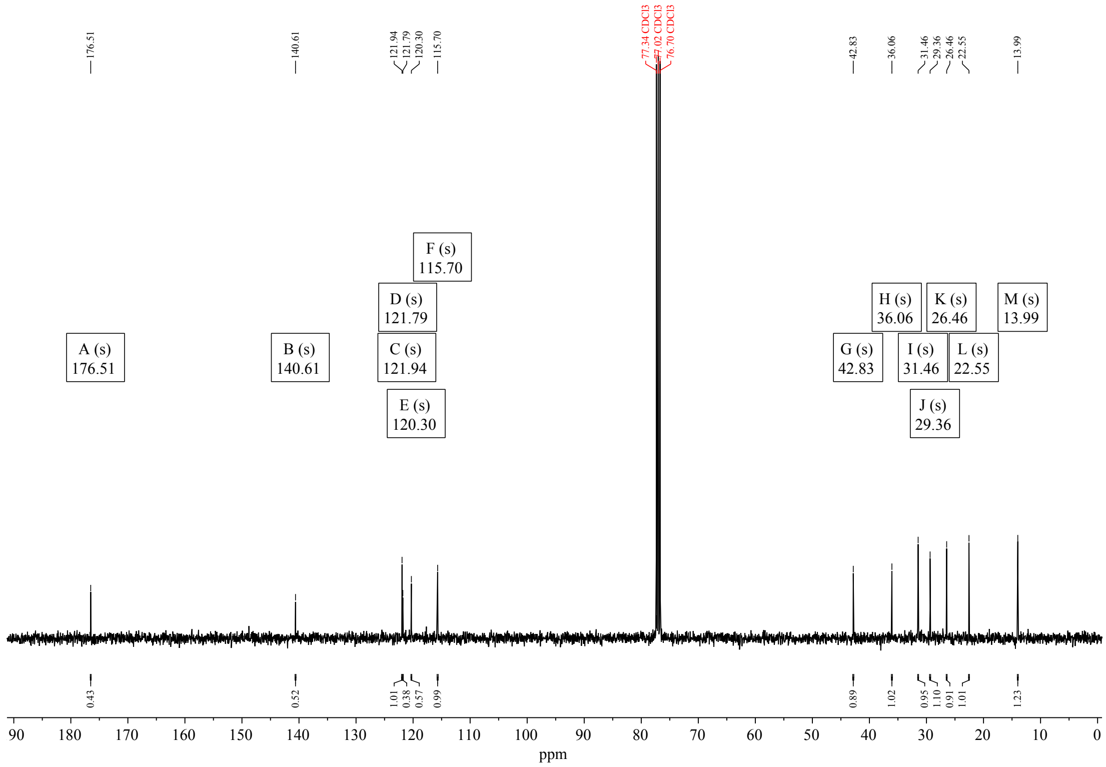

Figure S8. ^{13}C NMR spectrum of compound **(3b) C$_6$N** monomer.

3,8-dioctyl-1,3,6,8-tetrahydroindolo[7,6-*g*]indole-2,7-dione (3c) C$_8$N monomer: was synthesized following the general procedure for step (iii), 227 mg, 0.49 mmol, 26 %. ^{1}H NMR (400 MHz, $CDCl_3$) δ 7.92 (d, J = 8.6 Hz, 2H), 7.40 (d, J = 8.6 Hz, 2H), 4.28 – 4.20 (m, 4H),

3.67 (s, 4H), 1.81 (p, J = 7.6 Hz, 4H), 1.51 – 1.39 (m, 4H), 1.39 – 1.30 (m, 4H), 1.33 – 1.21 (m, 12H), 0.87 (t, J = 7.1 Hz, 6H). $^{13}C\{^1H\}$ NMR (101 MHz, $CDCl_3$) δ 176.7, 140.8, 122.1, 121.9, 120.4, 115.8, 43.0, 36.2, 31.9, 29.5, 29.4, 29.3, 26.9, 22.8, 14.2. MALDI-TOF: $[M+H]^+$ calc. ($C_{30}H_{42}N_2O_2$): 462.32 found: 463.33.

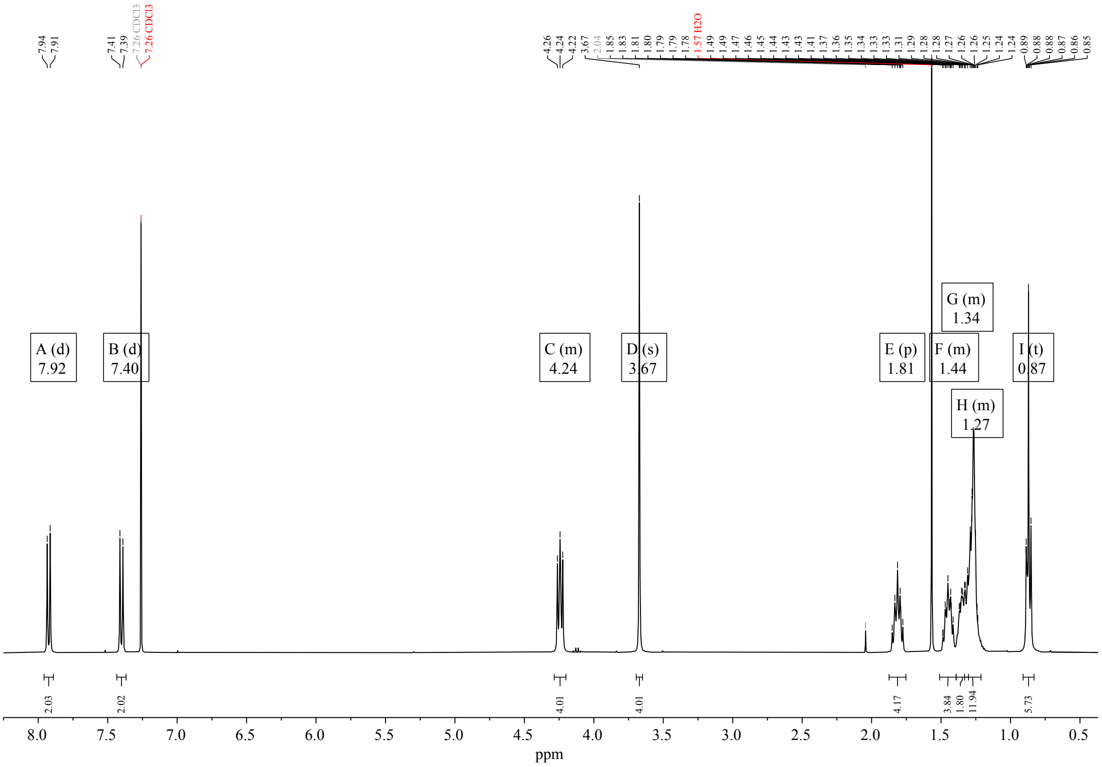

Figure S9. ^{1}H NMR spectrum of compound **(3c) C_8N** monomer.

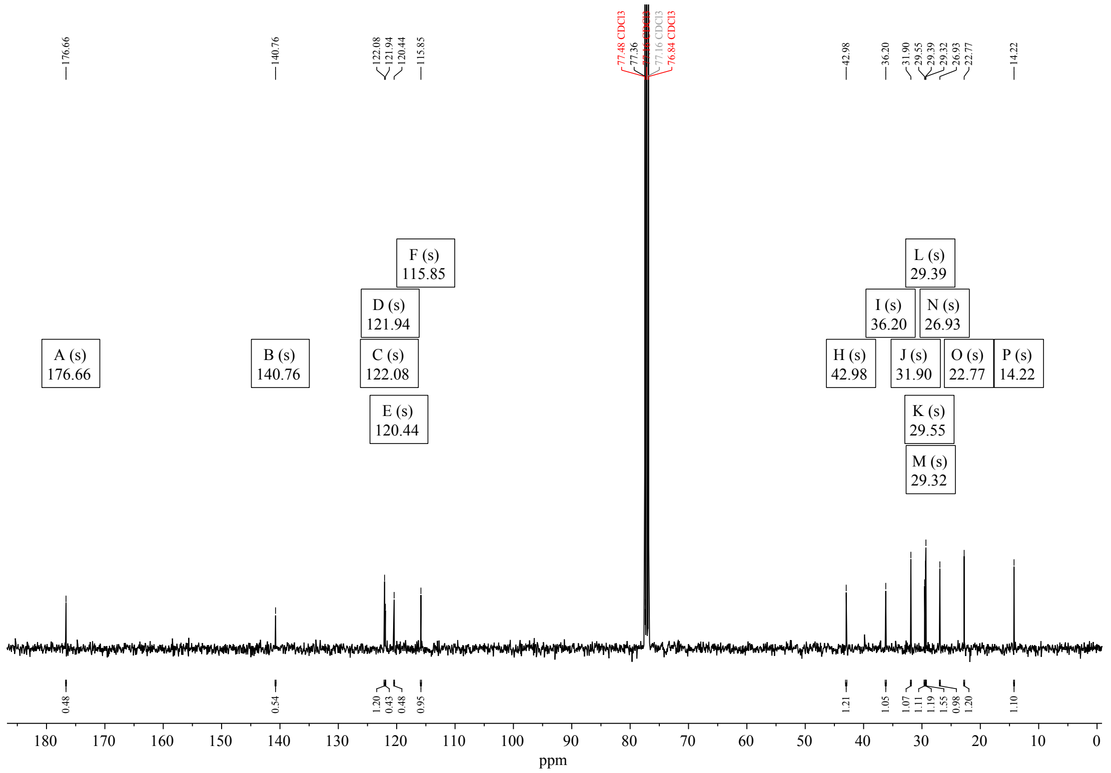

Figure S10. ^{13}C NMR spectrum of compound **(3c) C_8N** monomer.

3,8-didecyl-1,3,6,8-tetrahydroindolo[7,6-*g*]indole-2,7-dione (3d) C_{10}N monomer: was synthesized following the general procedure for step (iii), 469 mg, 0.90 mmol, 32 %. ^1H NMR (400 MHz, $CDCl_3$) δ 7.92 (d, J = 8.6 Hz, 2H), 7.40 (d, J = 8.6 Hz, 2H), 4.24 (t, J = 7.7 Hz, 4H), 3.67 (s, 4H), 1.81 (p, J = 7.6 Hz, 4H), 1.51 – 1.31 (m, 4H), 1.31 – 1.23 (m, 24H), 0.87 (t, J = 6.7 Hz, 6H). $^{13}C\{^1H\}$ NMR (101 MHz, $CDCl_3$) δ 176.7, 140.8, 122.1, 121.9, 120.4, 115.8, 43.0, 36.2, 32.0, 29.7, 29.6, 29.4, 26.9, 22.8, 14.3. MALDI-TOF: $[M+H]^+$ calc. ($C_{34}H_{50}N_2O_2$): 518.39 found: 519.40.

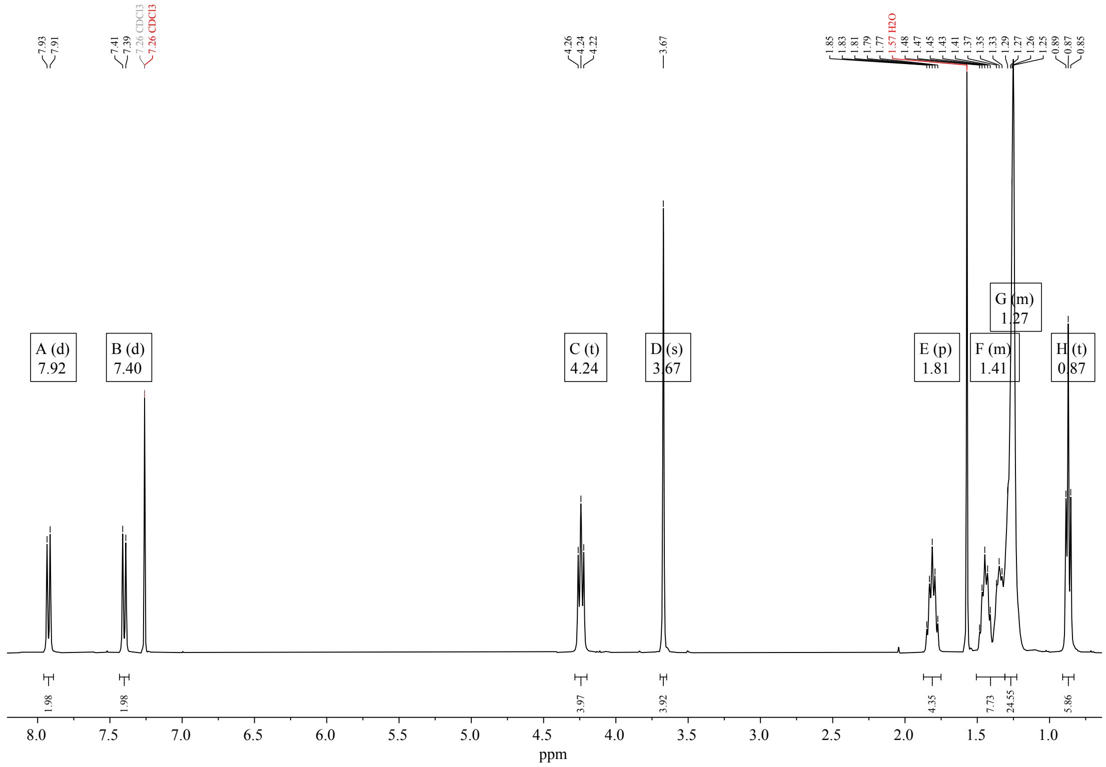

Figure S11. ^{1}H NMR spectrum of compound **(3d) $C_{10}N$** monomer.

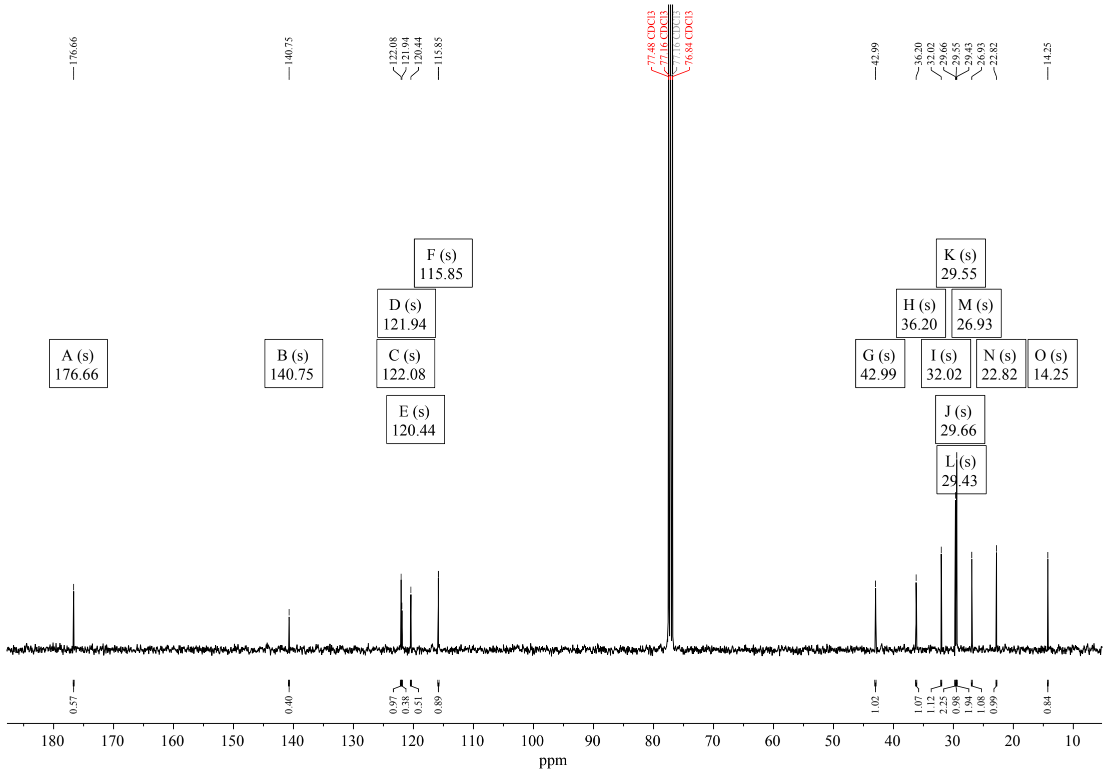

Figure S12. ^{13}C NMR spectrum of compound **(3d) $C_{10}N$** monomer.

3,8-dihexadecyl-1,3,6,8-tetrahydroindolo[7,6-*g*]indole-2,7-dione (3e) $C_{16}N$ monomer: was synthesized following the general procedure for step (iii), 224 mg, 0.33 mmol, 22 %. ^{1}H NMR (400 MHz, $CDCl_3$) δ 7.92 (d, *J* = 8.6 Hz, 2H), 7.40 (d, *J* = 8.6 Hz, 2H), 4.28 – 4.20 (m, 4H),

3.67 (s, 4H), 1.81 (p, J = 7.6 Hz, 4H), 1.44 (td, J = 8.8, 4.9 Hz, 4H), 1.26 – 1.23 (m, 48H), 0.88 (t, J = 6.8 Hz 6H). $^{13}C\{^1H\}$ NMR (101 MHz, $CDCl_3$) δ 176.6, 140.8, 122.1, 121.9, 120.4, 115.8, 42.9, 36.2, 32.1, 29.8, 29.8, 29.8, 29.7, 29.7, 29.6 29.5, 29.5, 26.9, 22.8, 14.3. MALDI-TOF: $[M+H]^+$ calc. ($C_{46}H_{74}N_2O_2$): 686.58 found: 687.59.

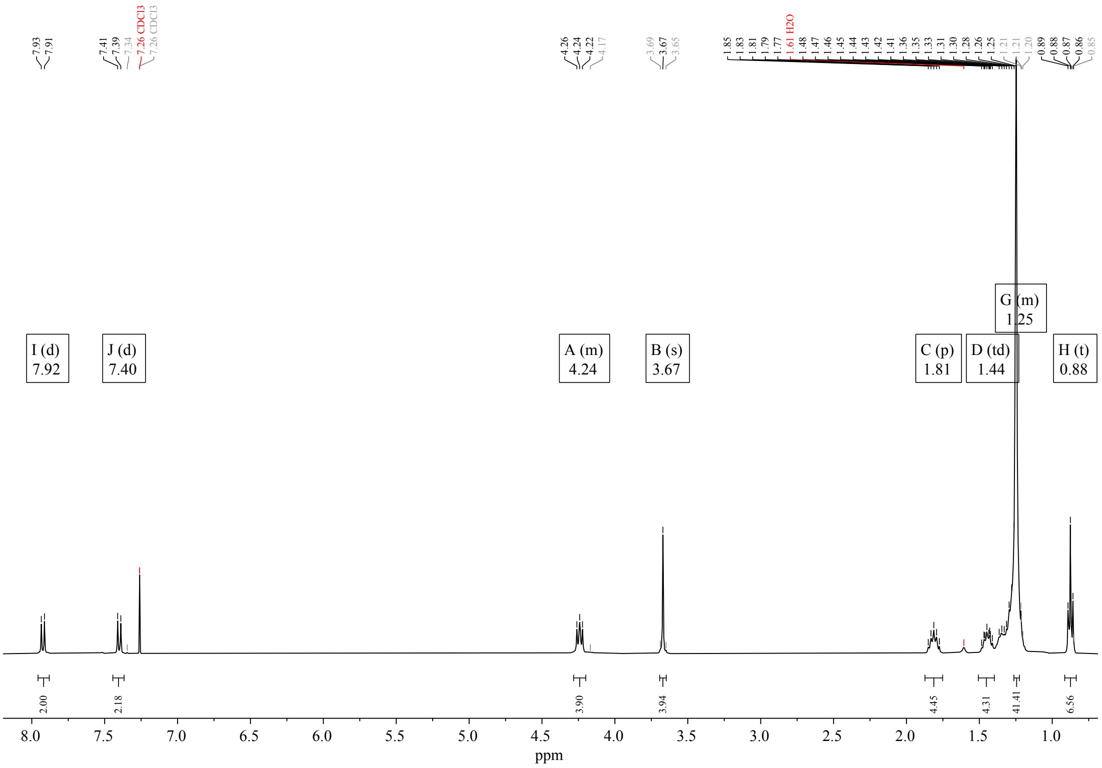

Figure S13. ^{1}H NMR spectrum of compound **(3e) C$_{16}$N** monomer.

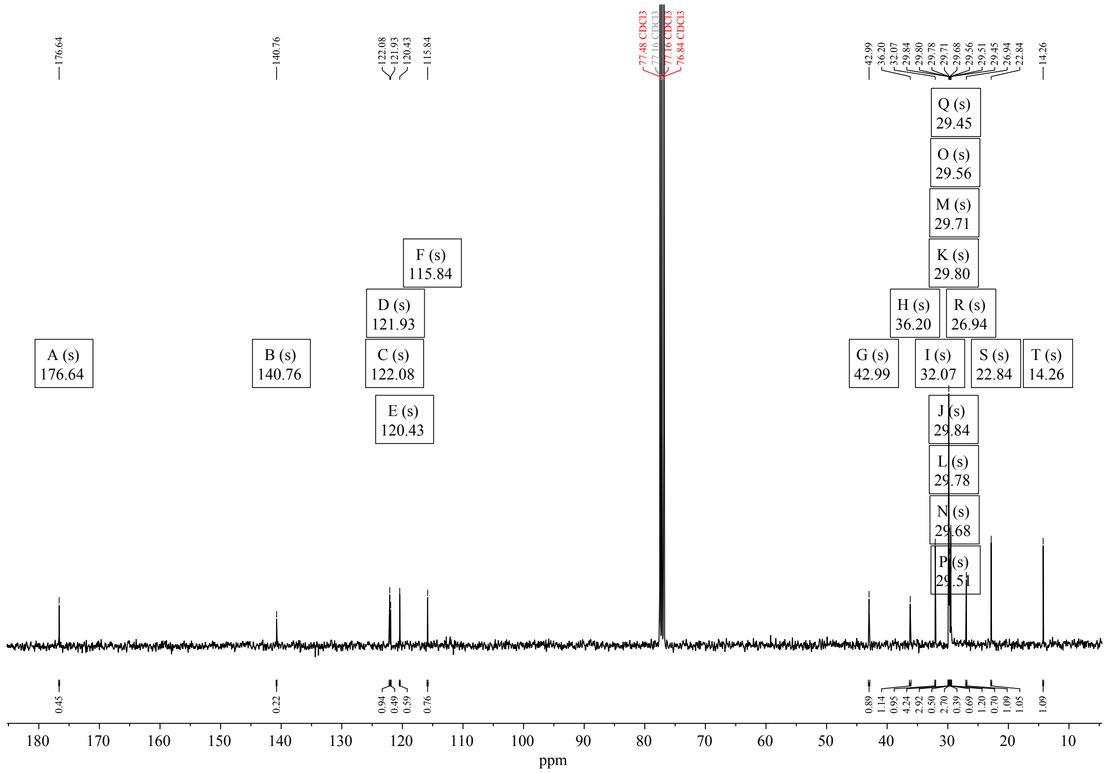

Figure S14. ^{13}C NMR spectrum of compound **(3e)** $C_{16}N$ monomer.

2.2.4. Synthesis of C_2N monomer:

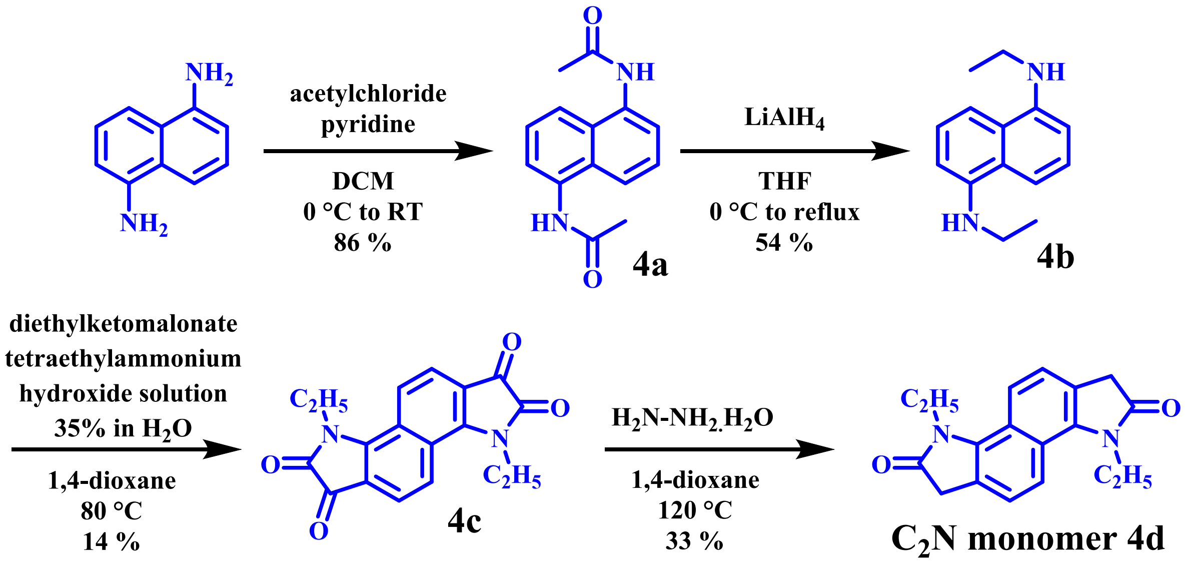

Figure S15. Full synthetic pathway from 1,5-diaminonaphthalene starting material to afford C_2N monomer **4d**.

***N,N'*-(naphthalene-1,5-diyl)diacetamide (4a):** 1,5-diaminonaphthalene (13.06 g, 82.6 mmol, 1.0 eq.) was suspended in 100 mL of anhydrous DCM and cooled to 0 °C. Pyridine (20.1 mL, 197.7 mmol, 2.4 eq.) was added dropwise followed by the dropwise addition of acetylchloride (12.96 mL, 181.6 mmol, 2.2 eq.). The reaction was stirred overnight before being filtered

through a frit and washed with copious amounts of hexane (300 mL). The solid was collected and oven dried to afford a white solid which was used without further purification, 16.1 g, 66.45 mmol, 86 %.

***N1,N5*-diethylnaphthalene-1,5-diamine (4b):** Compound **2a** (12 g, 49.5 mmol, 1.0 eq.) was suspended in 250 mL of anhydrous THF and cooled to 0 ºC. $LiAlH_4$ (2M in THF) (100 mL, 200 mmol 4.0 eq.) was slowly added dropwise, the mixture was then heated to reflux and stirred overnight. The reaction was then cooled back to 0 ºC, NaOH was carefully added dropwise until effervescence ceased. The reaction mixture was poured into water, washed with brine and extracted into DCM. The organic layer was separated and dried over $MgSO_4$. Solvent was removed under reduced pressure to yield a beige solid which was suspended in hexane and filtered to obtain the product was a beige powder, 5.7 g, 26.60 mmol, 54 %, which was used without further purification. ^{1}H NMR (400 MHz, $CDCl_3$) δ 7.34 – 7.21 (m, 4H), 7.15 (d, *J* = 8.7 Hz, 2H), 6.59 (d, *J* = 7.5 Hz, 2H), 3.29 (q, *J* = 7.1 Hz, 4H), 1.38 (t, *J* = 7.1 Hz, 6H). ^{13}C{^{1}H} NMR (101 MHz, $CDCl_3$) δ 144.6, 125.6, 124.0, 108.8, 104.5, 38.9, 14.9.

3,8-diethyl-3,8-dihydroindolo[7,6-*g*]indole-1,2,6,7-tetraone (4c): Compound **2b** (2 g, 9.3 mmol, 1.0 eq.) was dissolved in 15 mL of glacial acetic acid then heated and stirred at 150 ºC for ten minutes. Diethylketomalonate (6.4 mL, 42 mmol, 4.5 eq.), dissolved in 5 mL of glacial acetic acid was added dropwise and the reaction was stirred overnight at 150 ºC. The reaction mixture was cooled to room temperature and the solvent was removed under reduced pressure. The deep red mixture was passed through a shirt pad of silica, eluting with ethyl acetate. The solvent was removed, and the deep red solid was then dissolved in 60 mL of 1,4-dioxane, the reaction was stirred above a pre-heated oil bath. Tetraethylammonium hydroxide (35 % in H_2O, 15 mL) was added, and the reaction was placed into the pre-heated 80 oil bath and stirred for seven minutes. The reaction was quickly cooled to 0 and quenched with 2M HCl, before being poured into water and extracted into DCM. The organic layer was separated and dried over

$MgSO_4$. The solvent was once again removed under reduced pressure to yield the crude product as a deep purple solid. The crude was purified by column chromatography on silica gel using DCM:acetone (98:2) as the eluent. The deep blue product was suspended in hot hexane and filtered to afford the product as a cerulean powder, 414 mg, 1.28 mmol, 14 %. ^{1}H NMR (400 MHz, $CDCl_3$) δ 7.99 (d, *J* = 8.7 Hz, 2H), 7.69 (d, *J* = 8.6 Hz, 2H), 4.33 (q, *J* = 7.2 Hz, 4H), 1.49 (t, *J* = 7.2 Hz, 6H).

3,8-diethyl-1,3,6,8-tetrahydroindolo[7,6-*g*]indole-2,7-dione (4d) C_2N monomer: Compound **2c** (150 mg, 0.47 mmol, 1.0 eq.) was added to a 10 mL oven dried microwave vial. The vial was capped and degassed for 5 minutes prior to the addition of 2 mL anhydrous 1,4-dioxane, followed by the addition of 1.5 mL of hydrazine monohydrate. The vial was placed into an oil bath and heated overnight at 120 ºC, the vial was then cooled to 0 ºC before being poured into 100 mL of brine. The organic layer was extracted into DCM and dried over $MgSO_4$. Solvent was removed under reduced pressure and the crude residue was purified by column chromatography, on silica gel, using DCM:acetone (98:2) as the eluent system. The pure product was obtained as a beige powder, 41.1 mg, 0.14 mmol, 33 %. ^{1}H NMR (400 MHz, $CDCl_3$) δ 7.97 (d, *J* = 8.6 Hz, 2H), 7.41 (d, *J* = 8.6 Hz, 2H), 4.33 (q, *J* = 7.1 Hz, 4H), 3.67 (s, 4H), 1.45 (t, *J* = 7.1 Hz, 6H). ^{13}C{^{1}H} NMR (101 MHz, $CDCl_3$) δ 176.5, 140.7, 122.2, 121.8, 120.5, 115.8, 37.8, 36.3, 14.9. MALDI-TOF: $[M+H]^+$ calc. ($C_{18}H_{18}N_2O_2$): 294.14 found: 295.15.

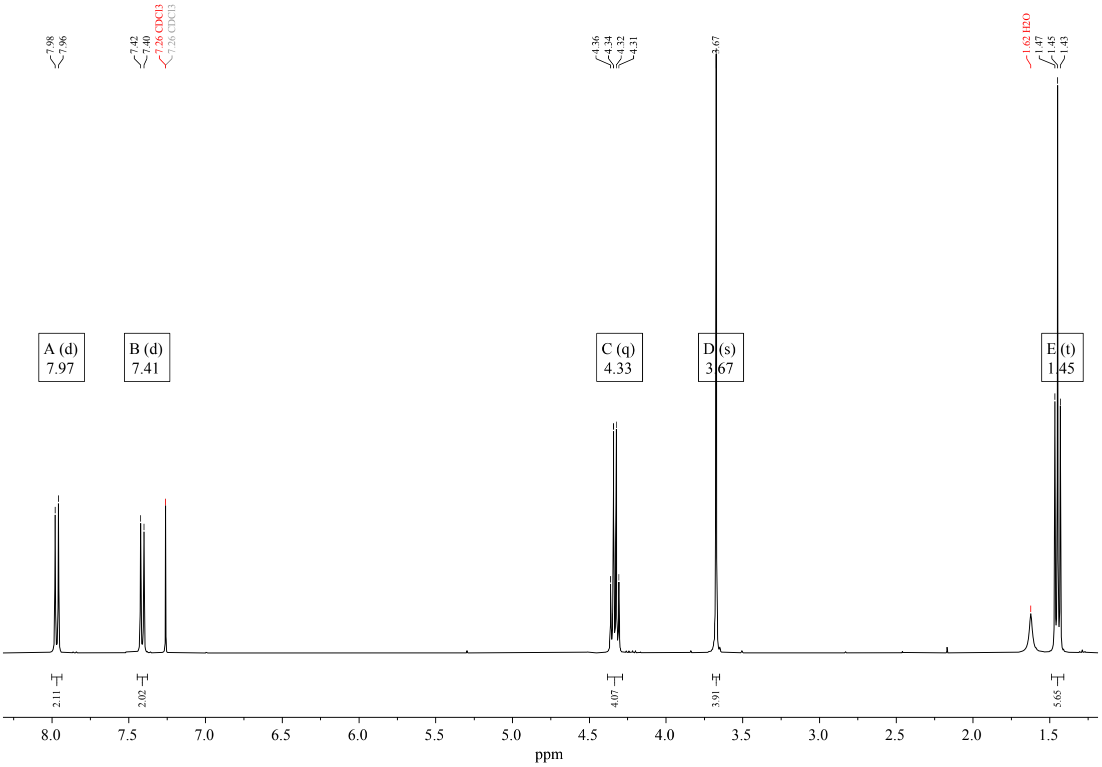

Figure S16. ^{1}H NMR spectrum of compound **(4d) C_2N** monomer.

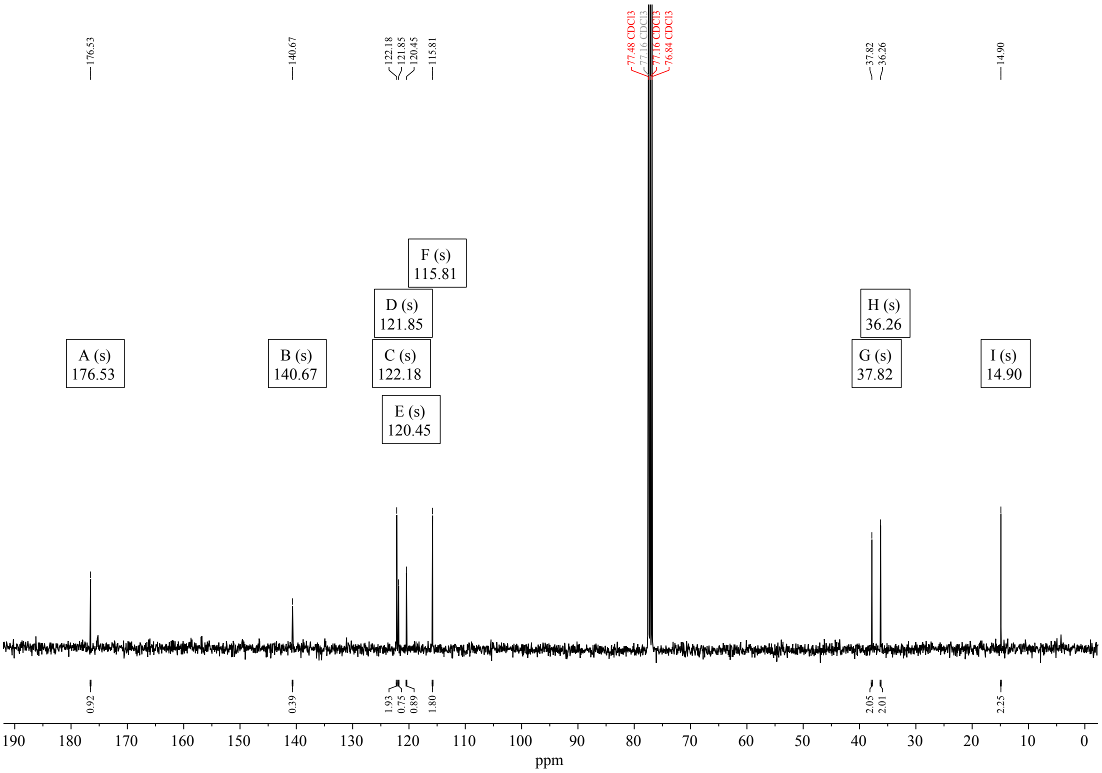

Figure S17. ^{13}C NMR spectrum of compound **(4d) C_2N** monomer.

2.3. Polymer synthesis:

2.3.1. General polymerization procedure (iv):

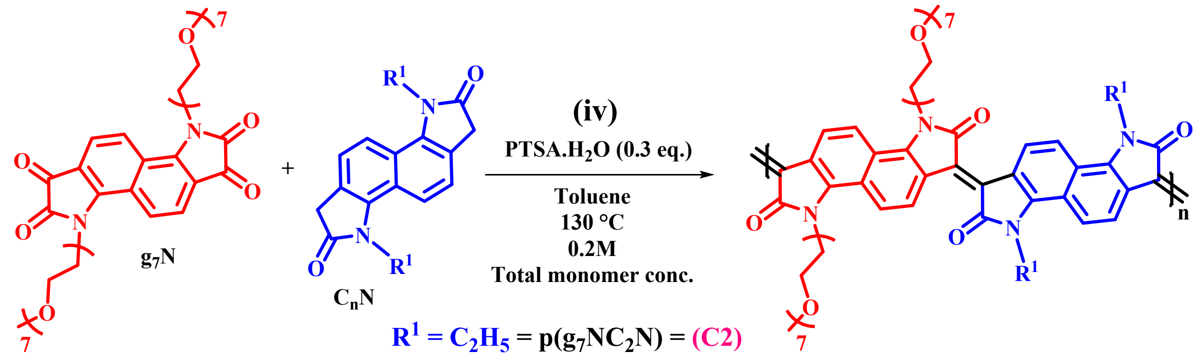

$R^1 = C_2H_5$ = p(g_7NC_2N) = (C2)
$R^1 = C_4H_9$ = p(g_7NC_4N) = (C4)
$R^1 = C_6H_{13}$ = p(g_7NC_6N) = (C6)
$R^1 = C_8H_{17}$ = p(g_7NC_8N) = (C8)
$R^1 = C_{10}H_{21}$ = p($g_7NC_{10}N$) = (C10)
$R^1 = C_{12}H_{25}$ = p($g_7NC_{12}N$) = (C12)
$R^1 = C_{16}H_{33}$ = p($g_7NC_{16}N$) = (C16)

Figure S18. General aldol condensation polymerization conditions to afford **p(g_7NC_nN)** polymers.

An oven dried 10 mL microwave vial was charged with monomer **g_7N** (1.0 eq.), monomer **C_nN** (1.0 eq.) and p-toluenesulfonic acid monohydrate (0.3 eq.). The cap was sealed, the vial was then purged with nitrogen for 10 minutes. Degassed anhydrous toluene (3.5 mL) was added, the vial was placed into a pre-heated oil bath (120 °C) and stirred for 22 hours. Upon cooling to room temperature, the dark purple solution was precipitated into 80 mL of methanol, filtered into a thimble, and purified by successive Soxhlet extraction with methanol, acetone, hexane and chloroform. The chloroform fraction was collected and reduced under vacuum, the polymer was re-precipitated into methanol, filtered and dried to obtain a dark purple film.

2.3.2. p(g_7NC_2N): was synthesized following the general polymerization procedure (iv) combining g7N and C2N monomers, 64 mg, 53.45 μmol, 70 %. GPC (chloroform, 40 °C): Mn 6.2 kDa, Mw 9.7 kDa.

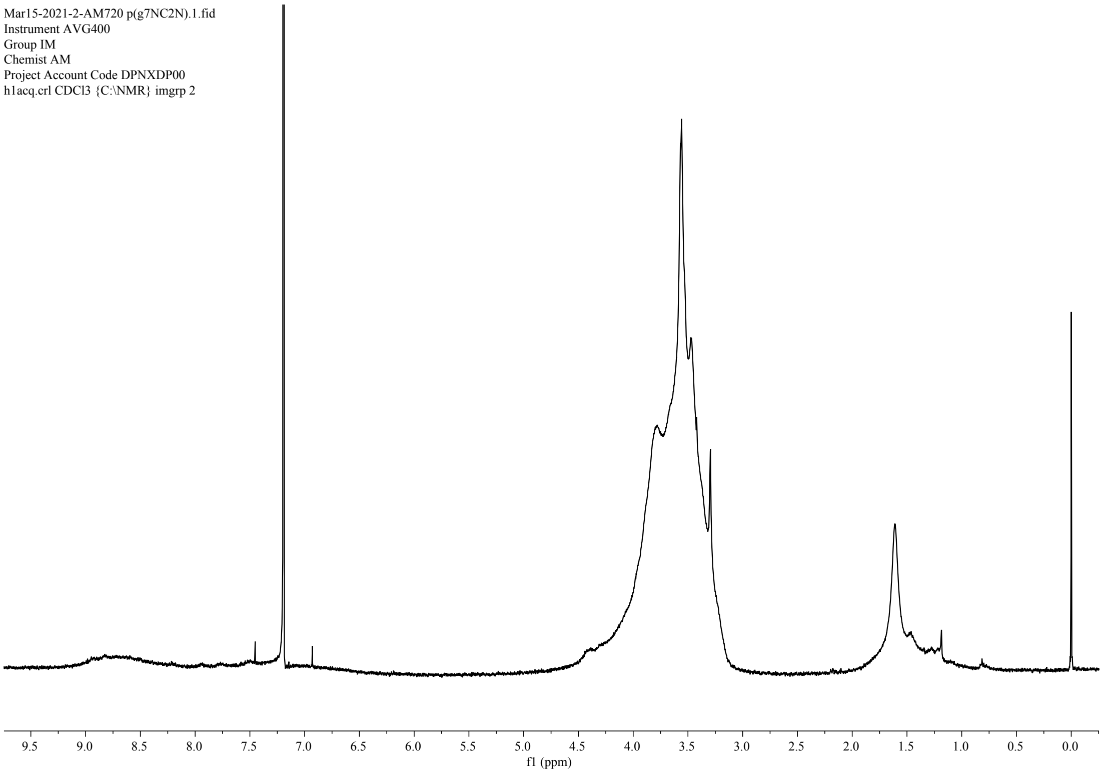

Figure S19. ^{1}H NMR of **p(g_7NC_2N)**.

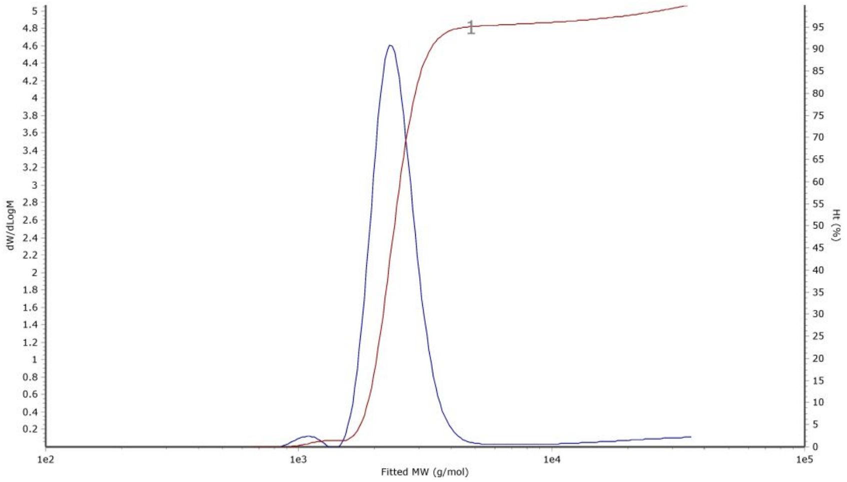

Figure S20. Gel-Permeation Chromatogram (GPC) spectra for p(g7NC2N). Mn, Mw, and PDI (Mw/Mn) were determined by GPC using low-D (<1.10) polystyrene standards and chloroform as the eluent at 40 °C.

2.3.3. p(g_7NC_4N): was synthesized following the general polymerization procedure (iv) combining g7N and C4N monomers, 88 mg, 70.20 µmol, 84 %. Mn 8.3 kDa, Mw 13.9 kDa.

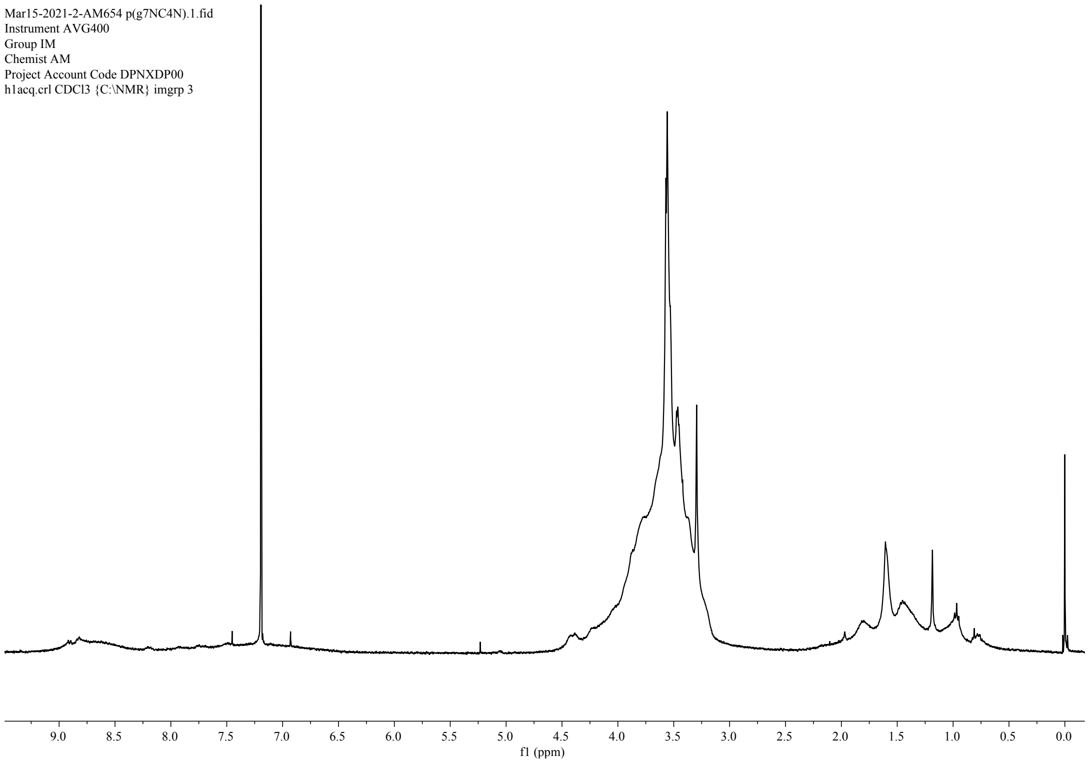

Figure S21. ^{1}H NMR of **p(g_7NC_4N)**.

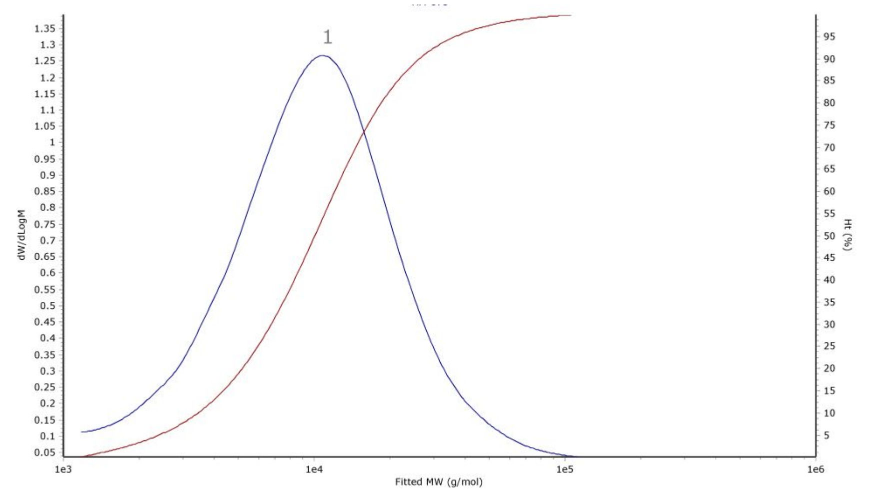

Figure S22. Gel-Permeation Chromatogram (GPC) spectra for **p(g_7NC_4N)**. Mn, Mw, and PDI (Mw/Mn) were determined by GPC using low-D (<1.10) polystyrene standards and chloroform as the eluent at 40 °C.

2.3.4. p(g_7NC_6N): was synthesized following the general polymerization procedure (iv) combining g_7N and C_6N monomers, 61 mg, 46.58 µmol, 70 %. Mn 10.2 kDa, Mw 15.1 kDa.

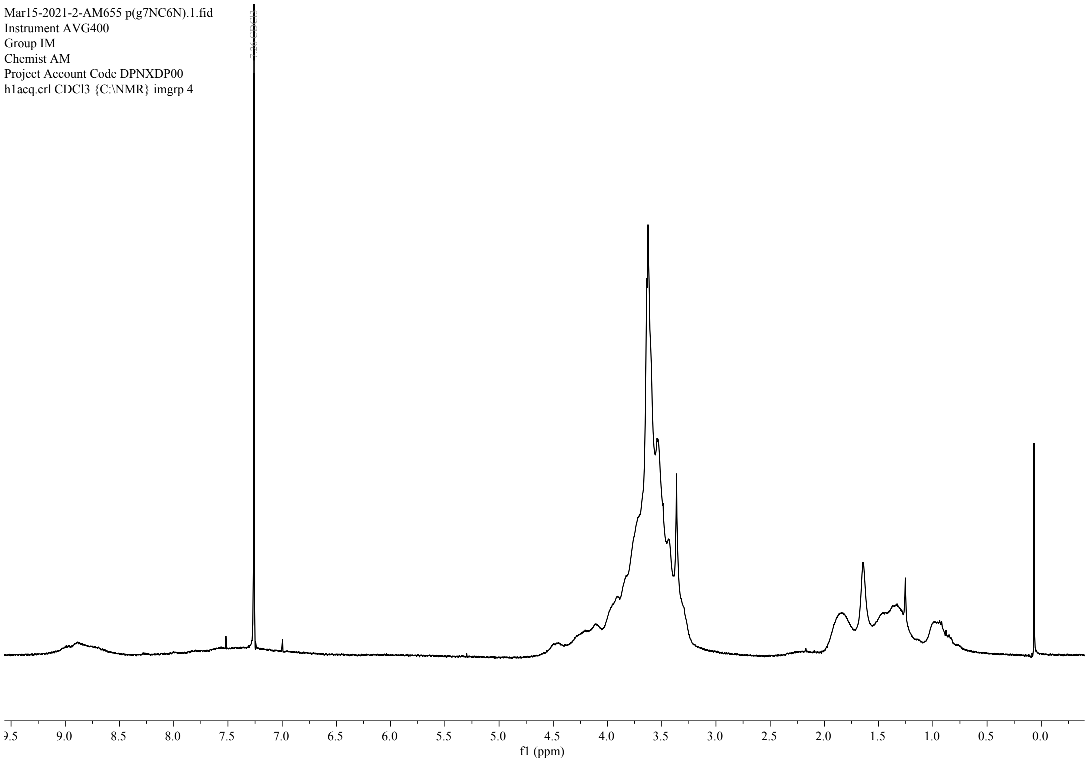

Figure S23. ^{1}H NMR of **p(g$_7$NC$_6$N)**.

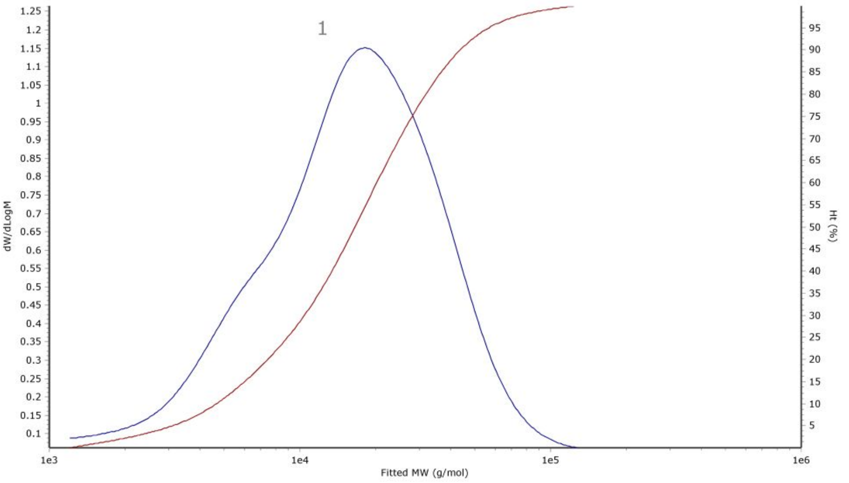

Figure S24. Gel-Permeation Chromatogram (GPC) spectra for **p(g$_7$NC$_6$N)**. Mn, Mw, and PDI (Mw/Mn) were determined by GPC using low-D (<1.10) polystyrene standards and chloroform as the eluent at 40 °C.

2.3.5. p(g_7NC_8N): was synthesized following the general polymerization procedure (iv) combining g_7N and C_8N monomers, 82 mg, 60.04 µmol, 77 %. Mn 15.0 kDa, Mw 19.9 kDa.

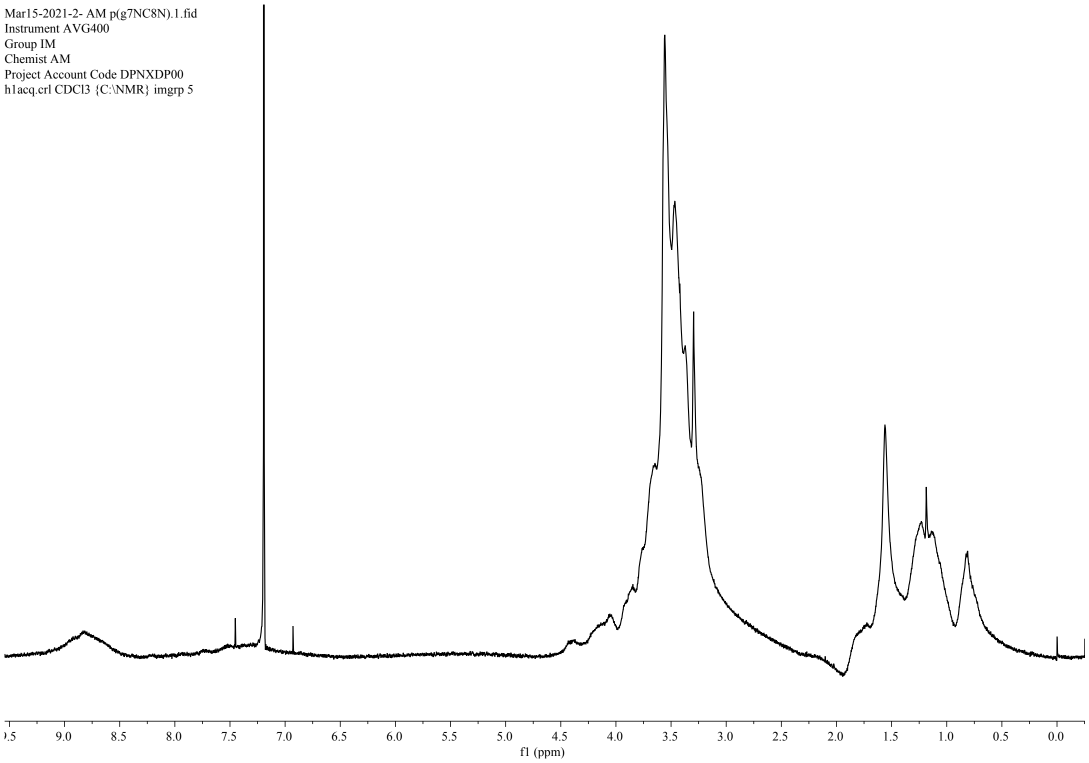

Figure S25. ^{1}H NMR of **p(g_7NC_8N)**.

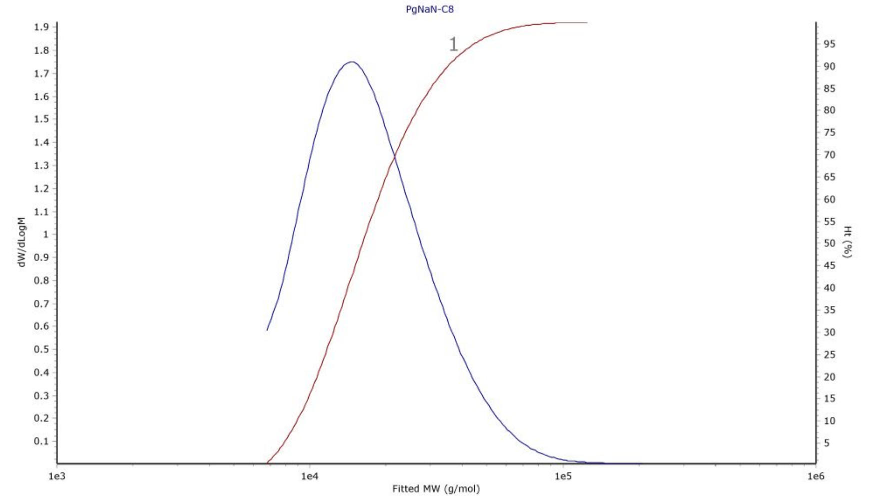

Figure S26. Gel-Permeation Chromatogram (GPC) spectra for **p(g_7NC_8N)**. Mn, Mw, and PDI (Mw/Mn) were determined by GPC using low-D (<1.10) polystyrene standards and chloroform as the eluent at 40 °C.

2.3.6. p($g_7NC_{10}N$): was synthesized following the general polymerization procedure (iv) combining g_7N and $C_{10}N$ monomers, 89 mg, 62.59 µmol, 79 %. Mn 20.3 kDa, Mw 34.8 kDa.

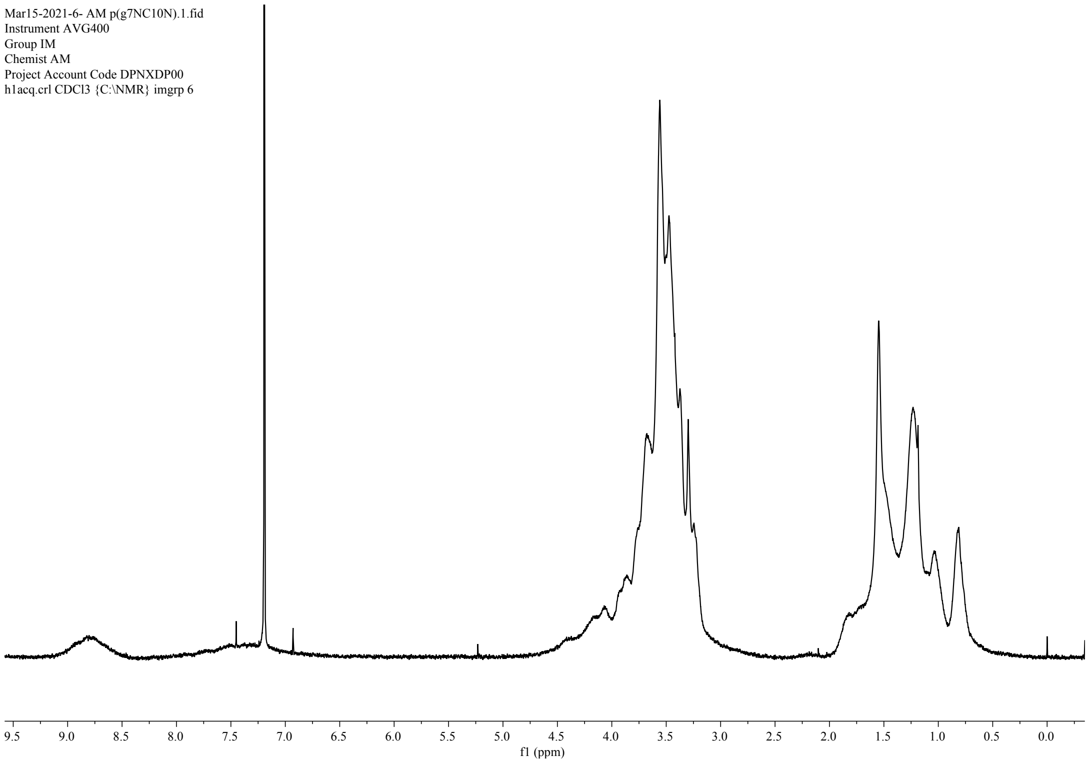

Figure S27. ^{1}H NMR of **p($g_7NC_{10}N$)**.

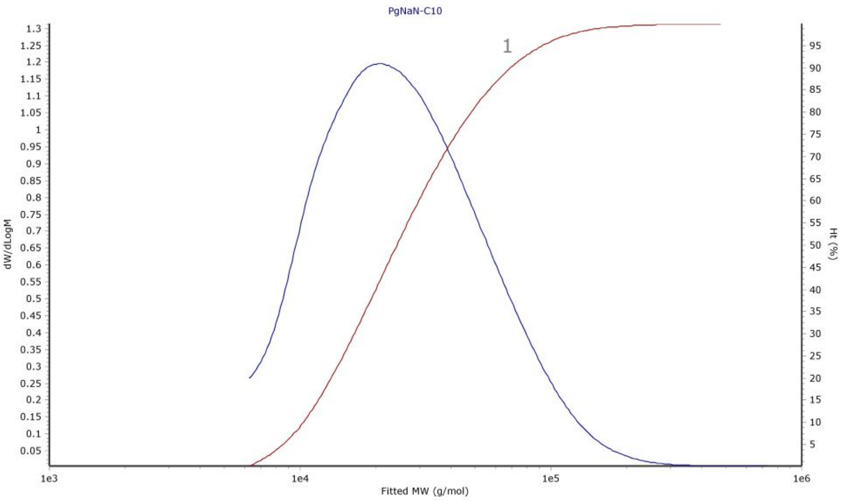

Figure S28. Gel-Permeation Chromatogram (GPC) spectra for **p($g_7NC_{10}N$)**. Mn, Mw, and PDI (Mw/Mn) were determined by GPC using low-D (<1.10) polystyrene standards and chloroform as the eluent at 40 °C.

2.3.7. p($g_7NC_{16}N$): was synthesized following the general polymerization procedure (iv) combining g_7N and $C_{16}N$ monomers, 55 mg, 34.59 µmol, 78 %. Mn 24.2 kDa, Mw 45.8 kDa.

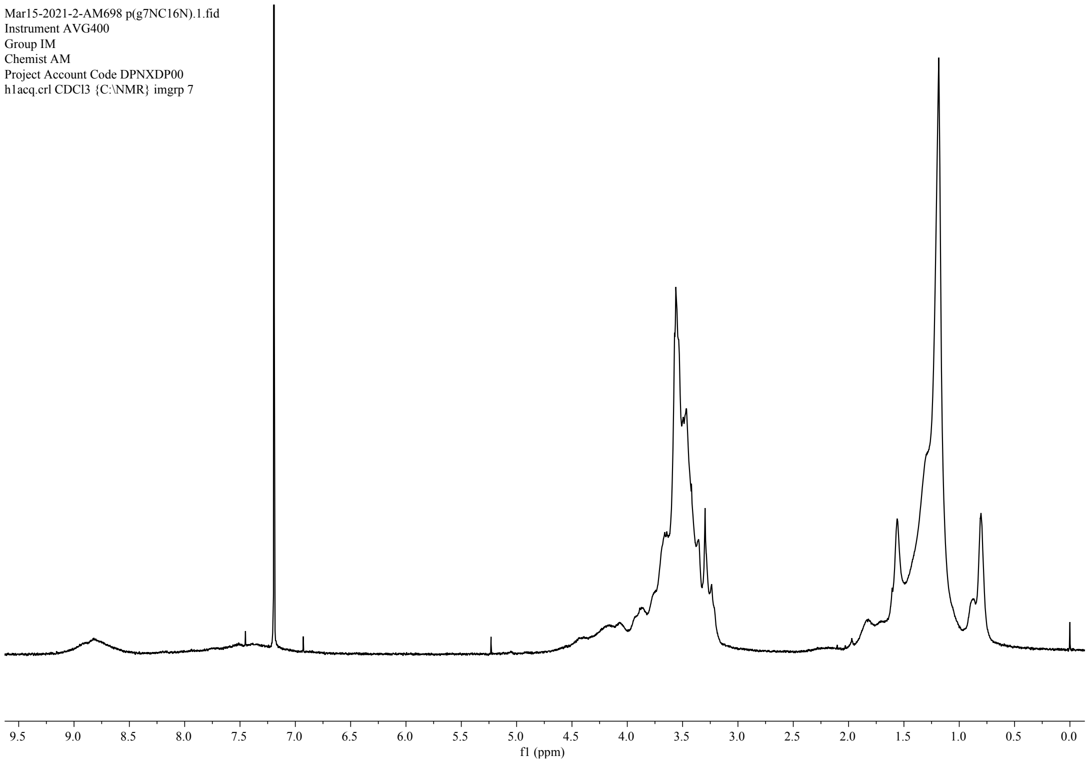

Figure S29. ^{1}H NMR of **p($g_7NC_{16}N$)**.

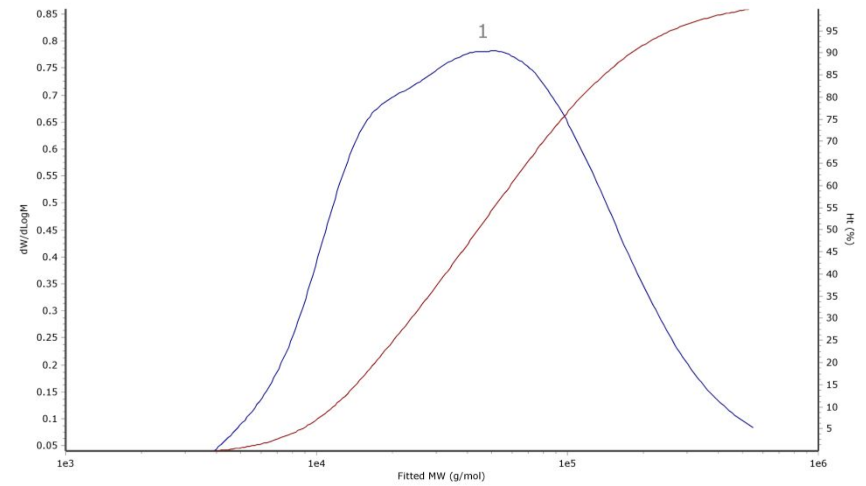

Figure S30. Gel-Permeation Chromatogram (GPC) spectra for **p($g_7NC_{16}N$)**. Mn, Mw, and PDI (Mw/Mn) were determined by GPC using low-D (<1.10) polystyrene standards and chloroform as the eluent at 40 °C.

3. POLYMER PROPERTIES

3.1. UV-Vis Absorption Spectroscopy

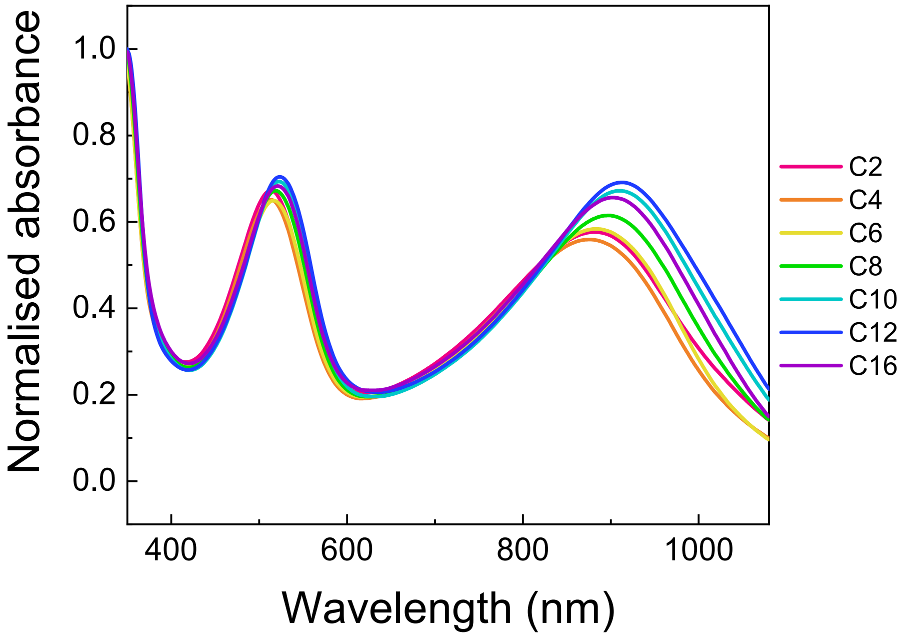

Figure S31. Solution UV-Vis absorption spectra for the entire **p(g$_7$NC$_n$N)** series.

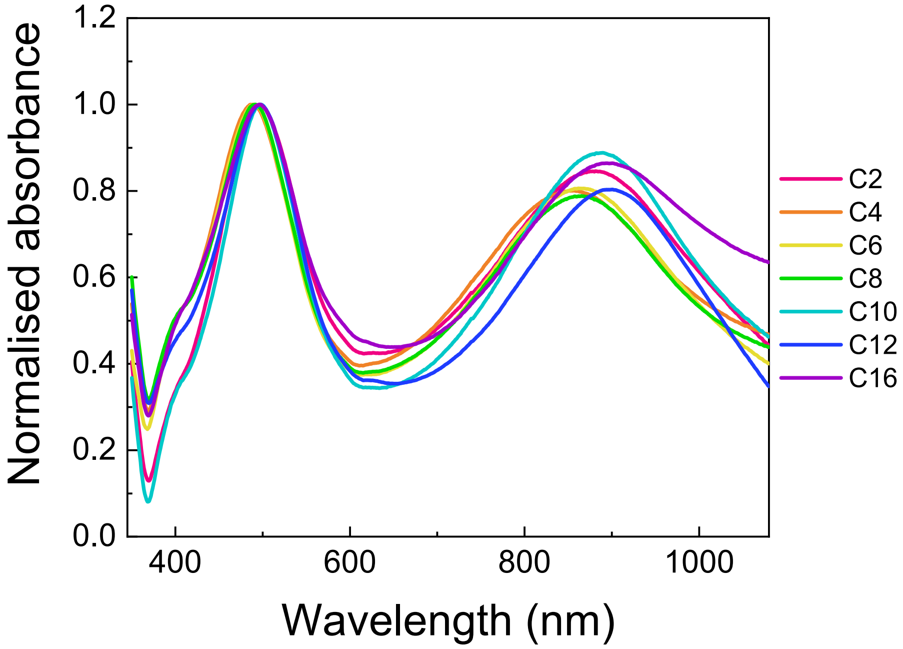

Figure S32. Thin-film UV-Vis absorption spectra for the entire **p(g$_7$NC$_n$N)** series.

3.2. Cyclic Voltammetry

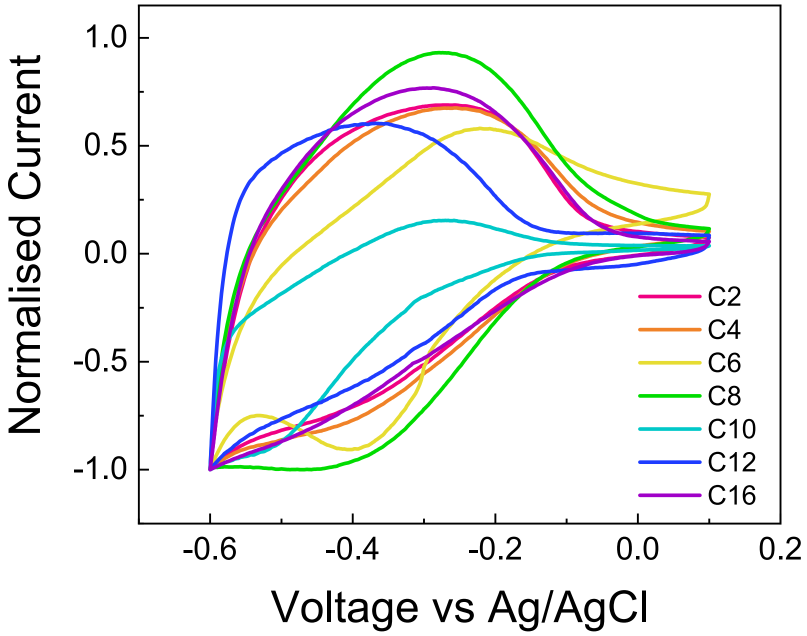

Figure S33. Organic electrolyte cyclic voltammetry spectra, obtained in 0.1 M tetrabutylammonium hexafluorophosphate in acetonitrile solution for the entire **p(g$_7$NC$_n$N)** series, acquired at a scan rate of 100 mV s^{-1}.

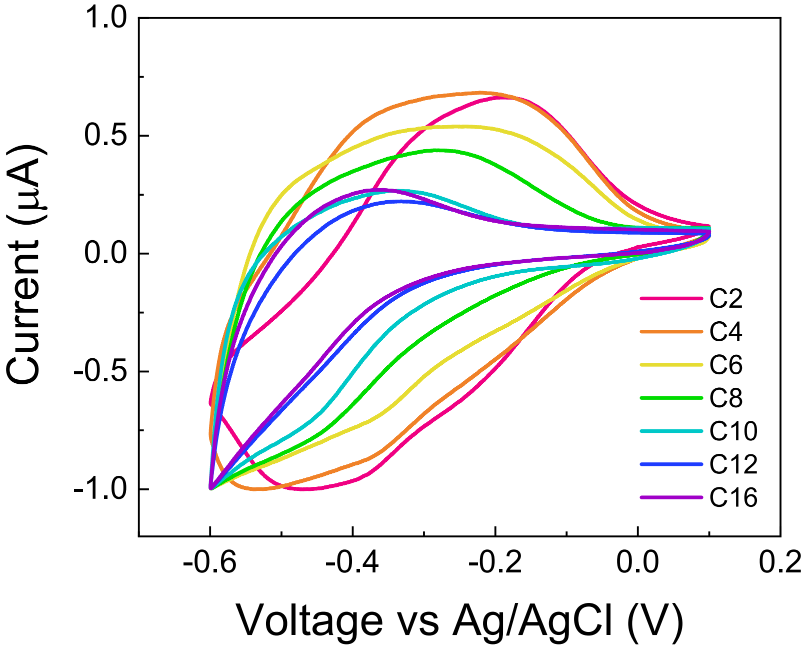

Figure S34. Aqueous electrolyte)cyclic voltammetry spectra, obtained in 0.1 M NaCl in H_2O for the entire **p(g$_7$NC$_n$N)** series, acquired at a scan rate of 100 mV s^{-1}.

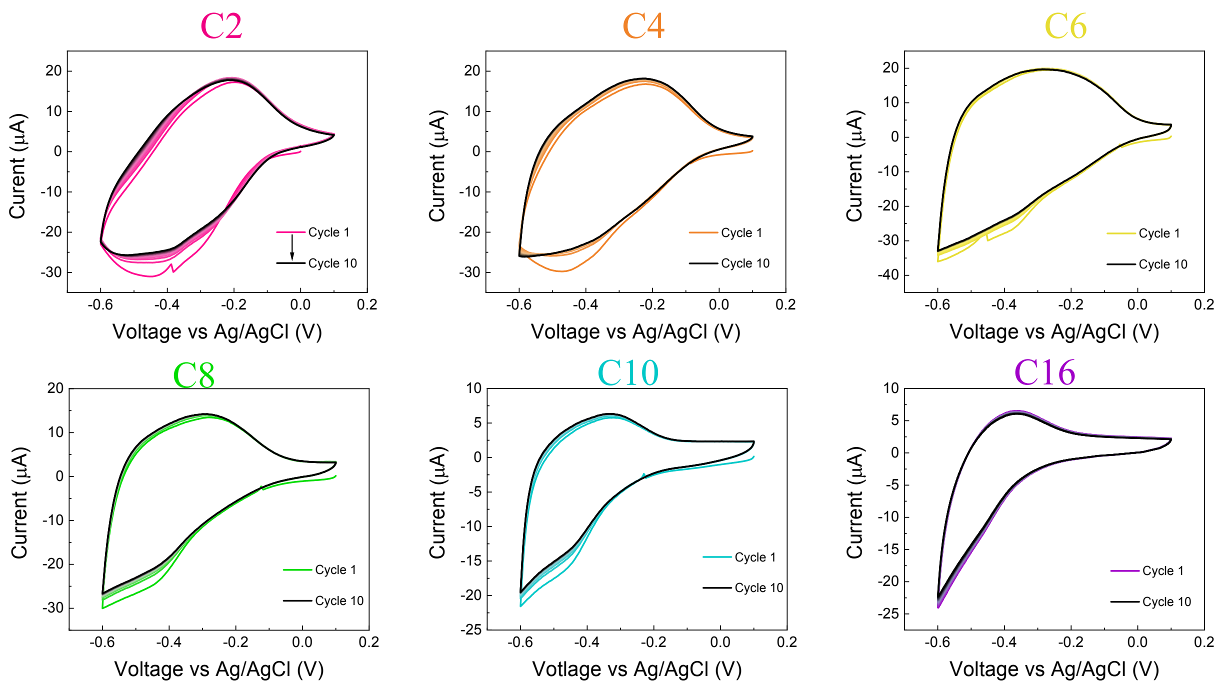

Figure S35. Stability cyclic voltammetry spectra, obtained in 0.1 M NaCl in H_2O for the entire **p(g$_7$NC$_n$N)** series, with ten cycles plotted for each polymer, acquired at a scan rate of 100 mV s^{-1}.

3.3. Spectroelectrochemistry

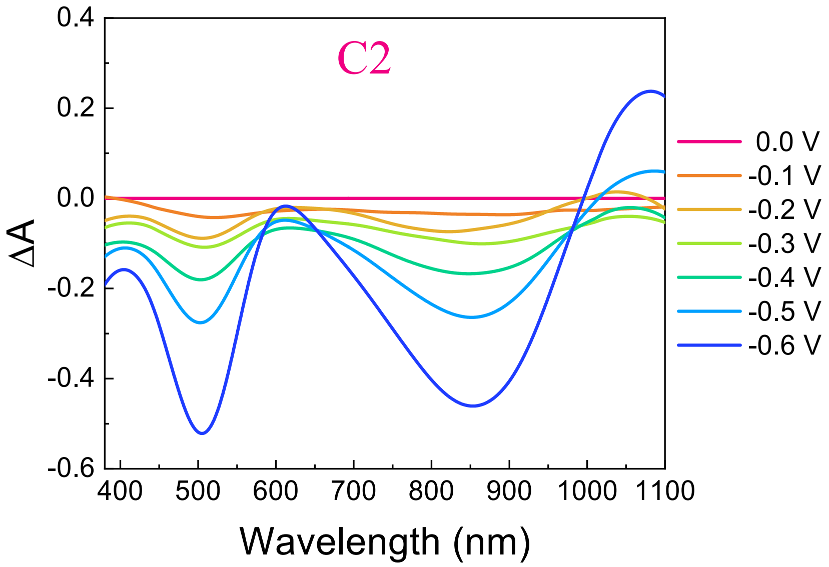

Figure S36. Spectroelectrochemistry of **p(g$_7$NC$_2$N)**, recording the change in normalized absorption upon increasing the applied reduction potential from 0 to -0.6 V, determined employing a 0.1 M NaCl in deionized water solution.

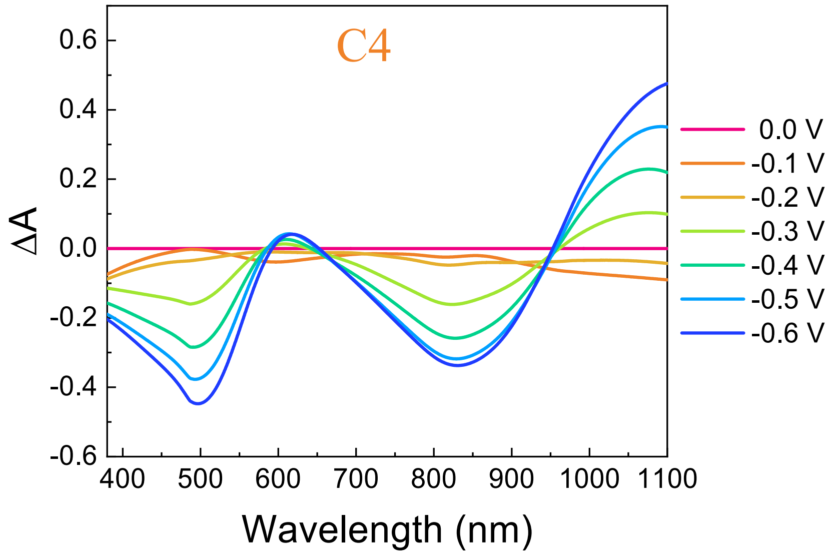

Figure S37. Spectroelectrochemistry of **p(g_7NC_4N)**, recording the change in normalized absorption upon increasing the applied reduction potential from 0 to -0.6 V, determined employing a 0.1 M NaCl in deionized water solution.

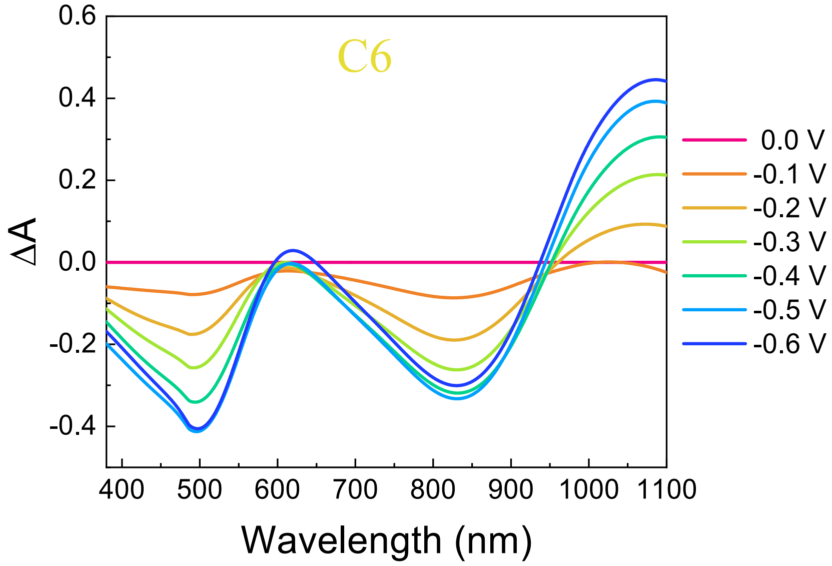

Figure S38. Spectroelectrochemistry of **p(g_7NC_6N)**, recording the change in normalized absorption upon increasing the applied reduction potential from 0 to -0.6 V, determined employing a 0.1 M NaCl in deionized water solution.

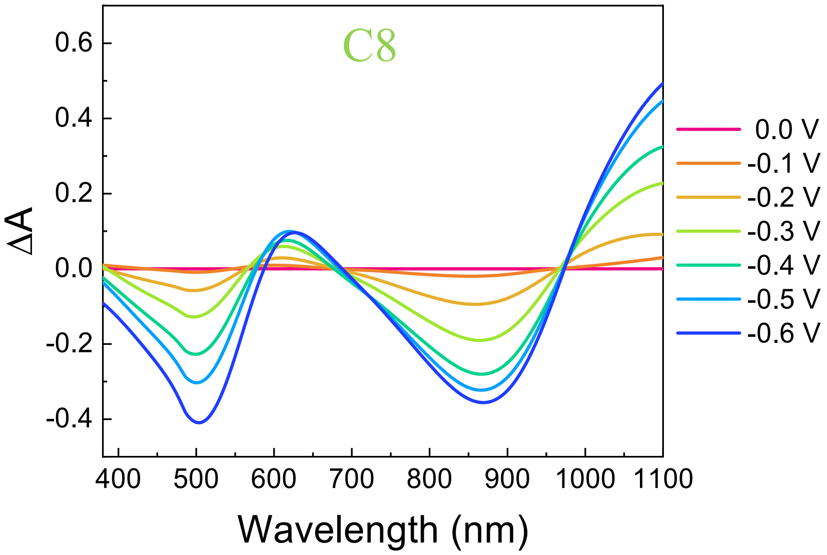

Figure S39. Spectroelectrochemistry of **p(g$_7$NC$_8$N)**, recording the change in normalized absorption upon increasing the applied reduction potential from 0 to -0.6 V, determined employing a 0.1 M NaCl in deionized water solution.

Figure S40. Spectroelectrochemistry of **p(g$_7$NC$_{10}$N)**, recording the change in normalized

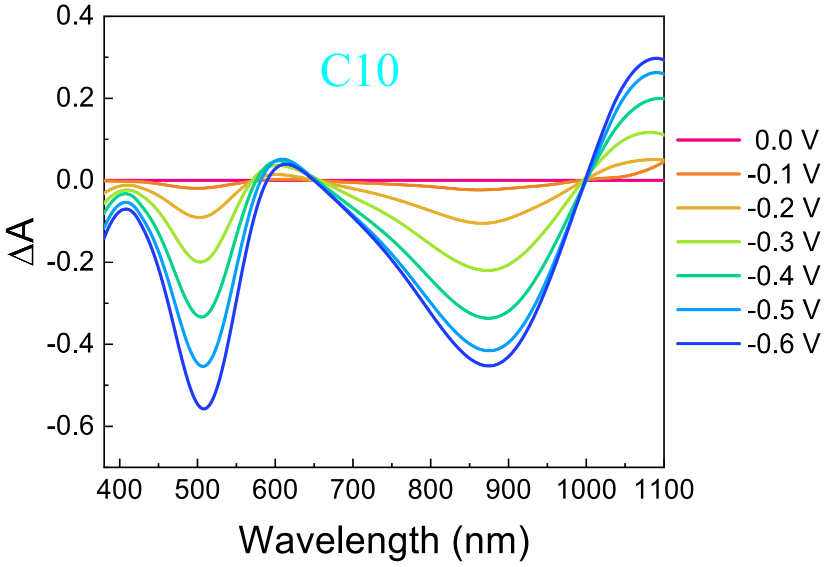

absorption upon increasing the applied reduction potential from 0 to -0.6 V, determined employing a 0.1 M NaCl in deionized water solution.

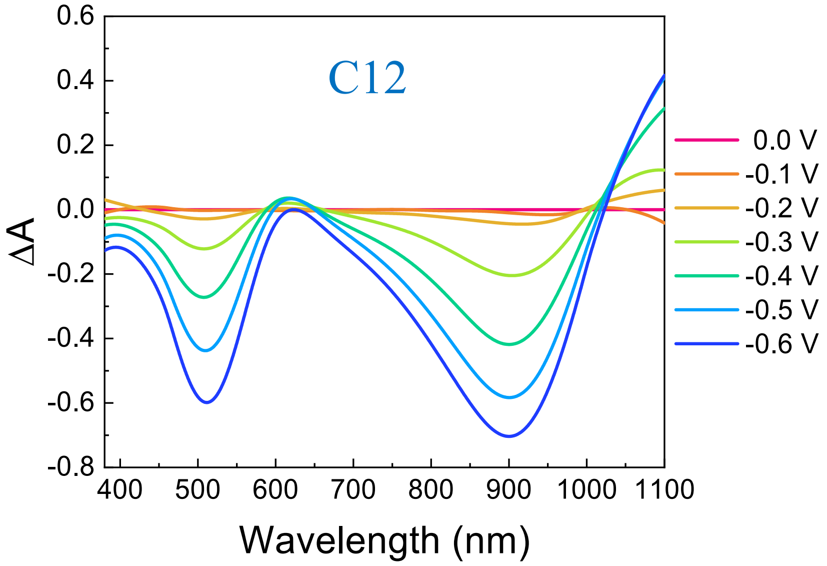

Figure S41. Spectroelectrochemistry of **p(g$_7$NC$_{12}$N)**, recording the change in normalized absorption upon increasing the applied reduction potential from 0 to -0.6 V, determined employing a 0.1 M NaCl in deionized water solution.

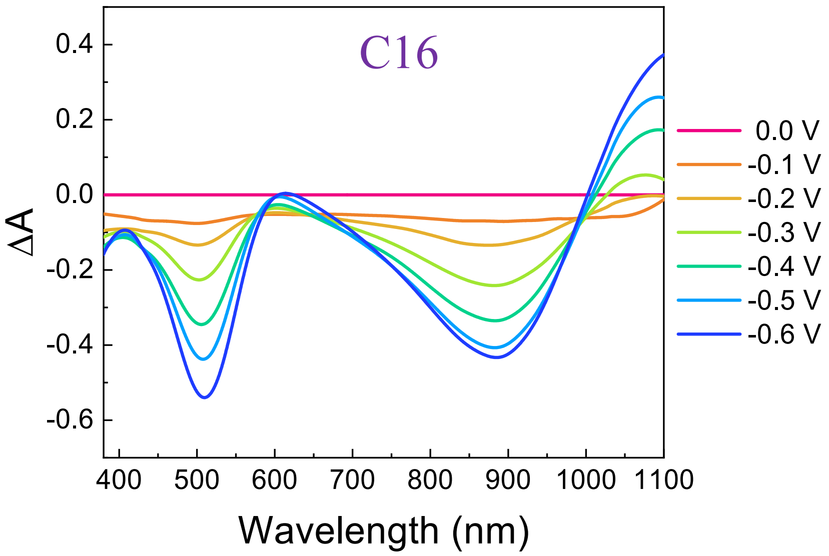

Figure S42. Spectroelectrochemistry of **p(g$_7$NC$_{16}$N)**, recording the change in normalized absorption upon increasing the applied reduction potential from 0 to -0.6 V, determined employing a 0.1 M NaCl in deionized water solution.

4. ORGANIC ELECTROCHEMICAL TRANSISTOR DATA

4.1. Electrochemical Impedance Spectroscopy

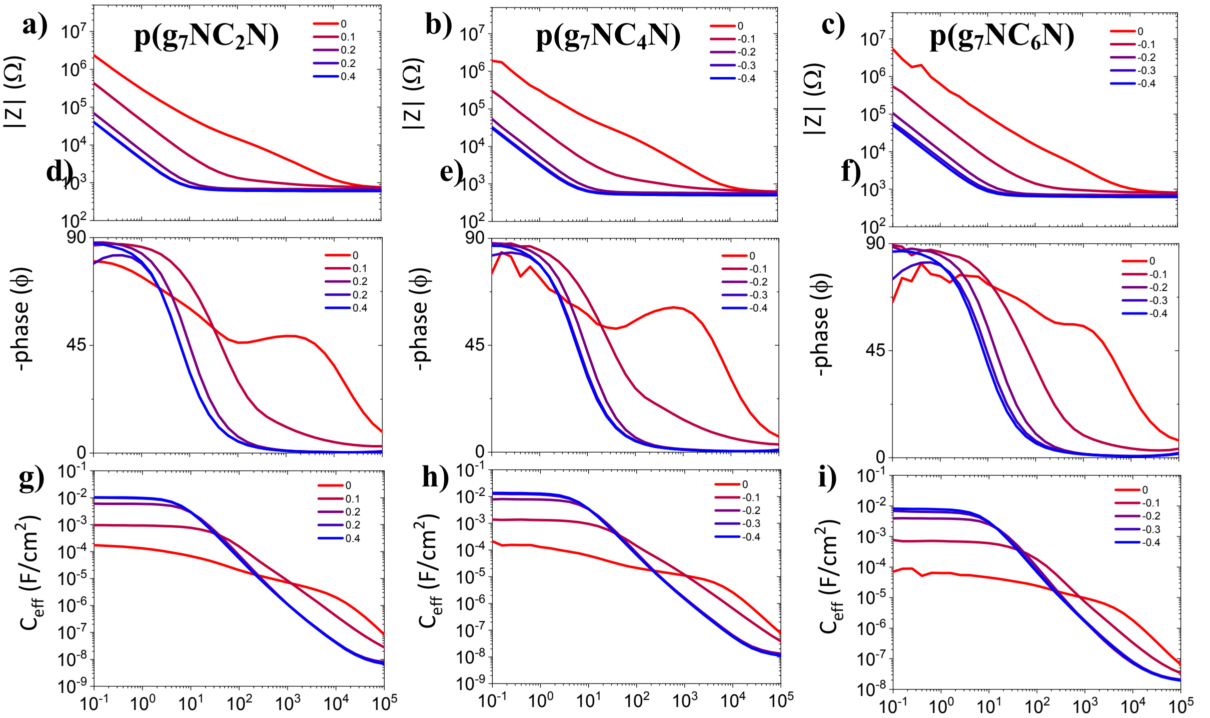

Figure S43. Electrochemical Impedance Spectroscopy: impedance (a,b,c), phase angle (d,e,f), and effective capacitance (g,h,i) of **p(g$_7$NC$_2$N)**, **p(g$_7$NC$_4$N)** and **p(g$_7$NC$_6$N)**, respectively.

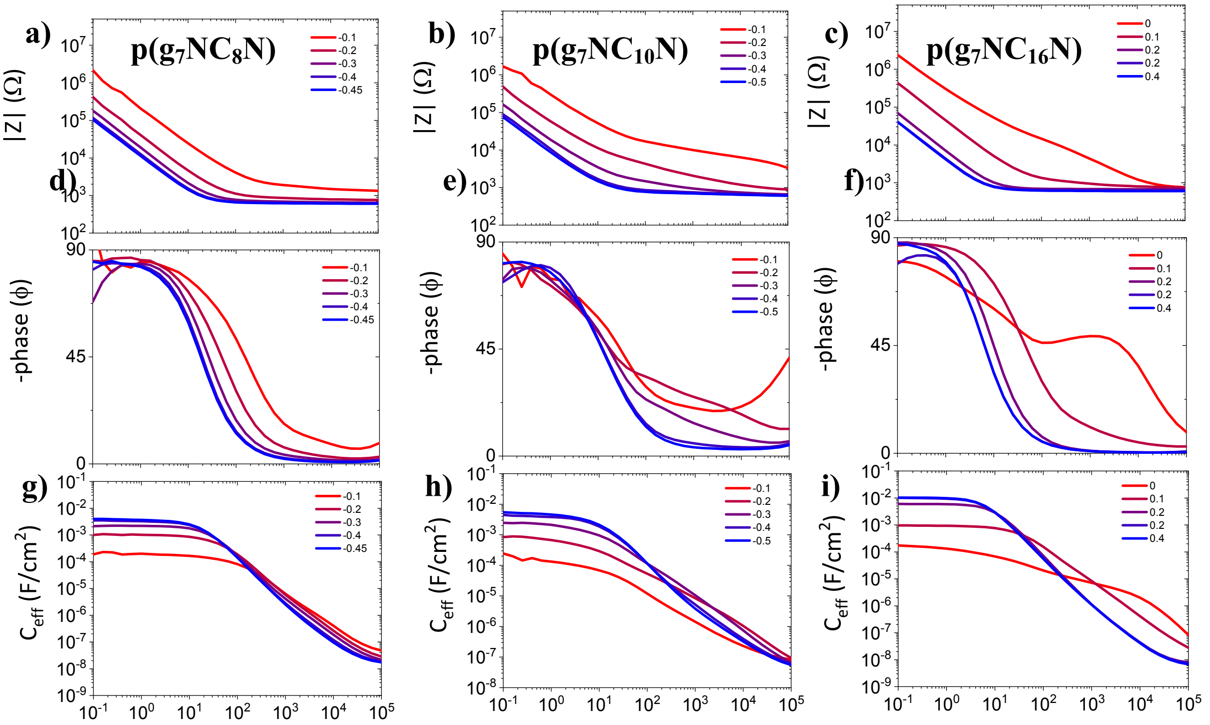

Figure S44. Electrochemical Impedance Spectroscopy: impedance (a,b,c), phase angle (d,e,f), and effective capacitance (g,h,i) of **p(g$_7$NC$_8$N)**, **p(g$_7$NC$_{10}$N)** and **p(g$_7$NC$_{16}$N)**, respectively.

4.2. OECT figures of merit Summary

Table S1. Summary of OECT parameters and material figures of merit of the polymers under investigation

**Polymer**	$^{a}\mu_{e,\,\mathrm{OECT}}$ **(cm^2 V^{-1} s^{-1})**	$^{b}C^*$ **(F cm^{-3})**	$^{c}\mu C^*$ **(F cm^{-1} V^{-1} s^{-1})**	g_m' **(S cm^{-1})**	$^{a}V_{th}$ **(mV)**	**Device Thickness (cm)**
**p(g$_7$NC$_2$N)**	(2.00 ± 0.41) ×10^{-3}	180 ± 16	0.36 ± 0.074	0.067	230 ± 3.3	(1.72 ± 2.61) ×10^{-4}
**p(g$_7$NC$_4$N)**	(1.46 ± 0.53) ×10^{-3}	126 ± 12	0.18 ± 0.067	0.035	210 ± 2.9	(4.60 ± 1.30) ×10^{-4}
**p(g$_7$NC$_6$N)**	(2.29 ± 0.70) ×10^{-3}	150 ± 4	0.34 ± 0.111	0.065	210 ± 6.4	(2.92 ± 0.43) ×10^{-4}
**p(g$_7$NC$_8$N)**	(6.01 ± 1.87) ×10^{-3}	199 ± 27	1.19 ± 0.371	0.240	250 ± 3.4	(4.37 ± 2.30) ×10^{-5}
**p(g$_7$NC$_{10}$N)**	(1.20 ± 0.07) ×10^{-2}	153 ± 34	1.83 ± 0.101	0.370	300 ± 3.0	(1.49 ± 0.26) ×10^{-4}
**p(g$_7$NC$_{12}$N)**[7]	(6.50 ± 1.01) ×10^{-3}	100 ± 6	0.66 ± 0.113	0.212	328 ± 5.3	(1.51 ± 2.70) ×10^{-5}
**p(g$_7$NC$_{16}$N)**	(3.80 ± 0.59) ×10^{-3}	86 ± 11	0.33 ± 0.074	0.047	360 ± 10.0	(1.33 ± 2.73) ×10^{-4}

[a]Saturation mobility and threshold voltage extracted from fits of $I_d^{1/2}$ vs V_g plots. [b]Average volumetric capacitance beyond threshold voltage determined by electrochemical impedance spectroscopy. Reported uncertainties are one standard deviation, with $n = 6$ devices. [c]Maximum transconductance and μC^* extracted from the slope of saturated transfer curves at −0.6 V.

4.3. Transfer, Output & Stability Curves

4.3.1. p(g_7NC_2N)

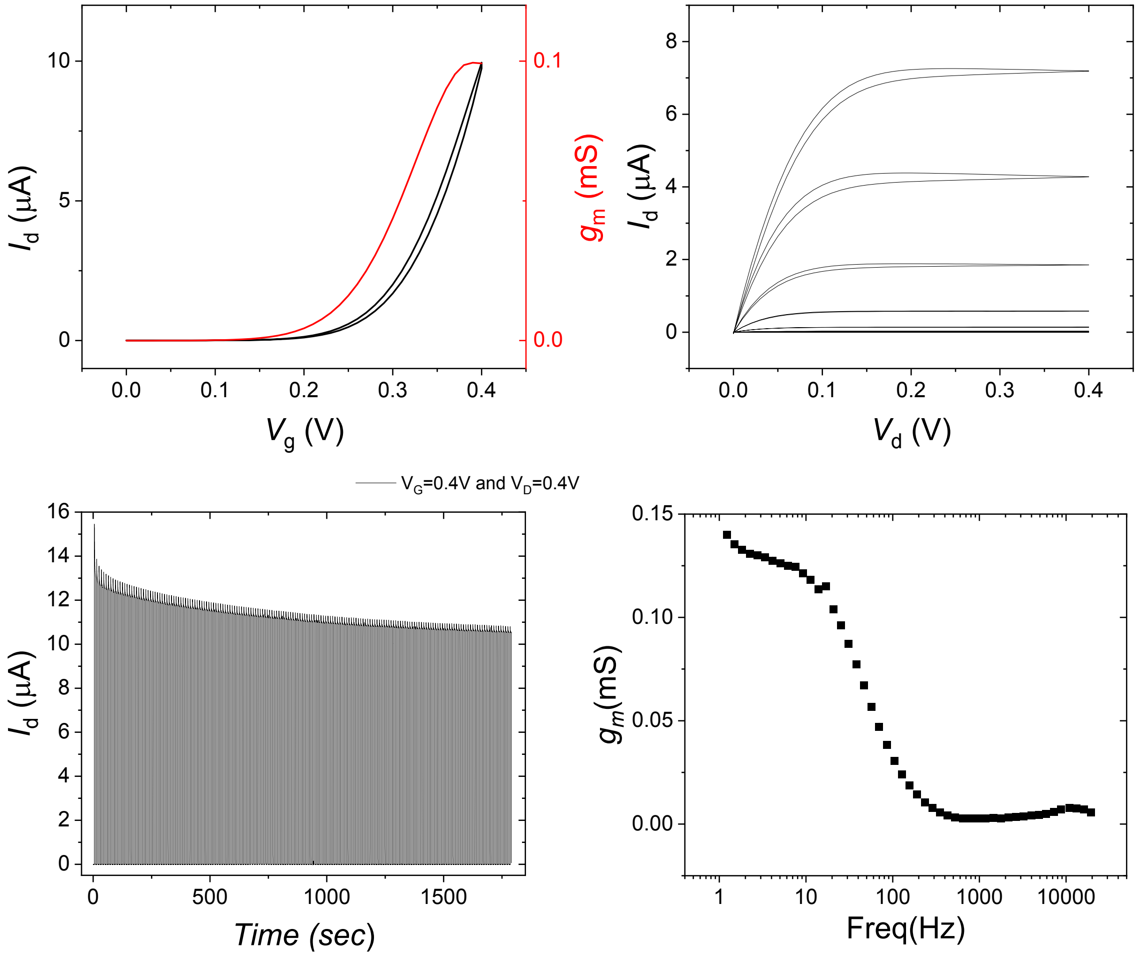

Figure S45. a) Transfer and transconductance curve, b) output curve, c) pulsed OECT stability and d) time dependent transconductance of a **p(g_7NC_2N)** containing OECT.

4.3.2. p(g_7NC_4N)

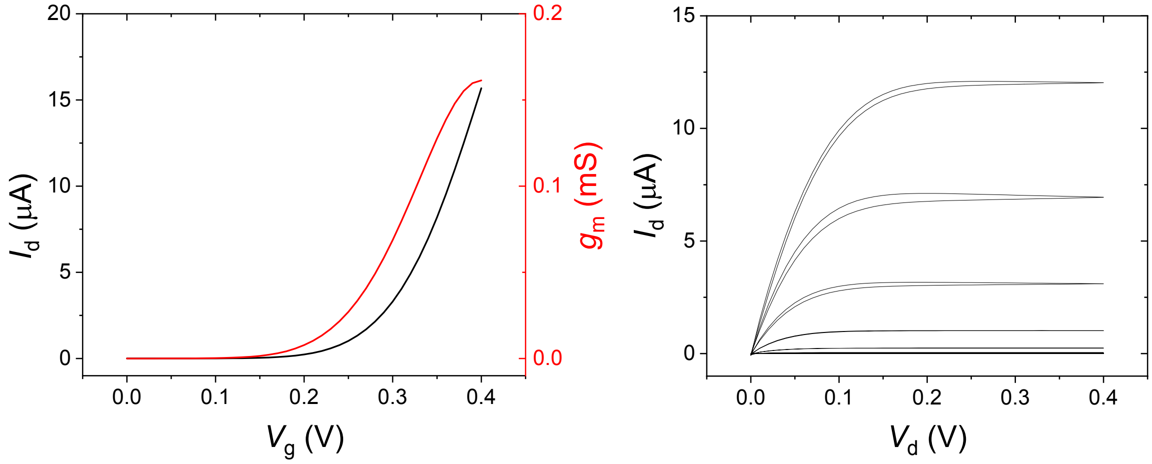

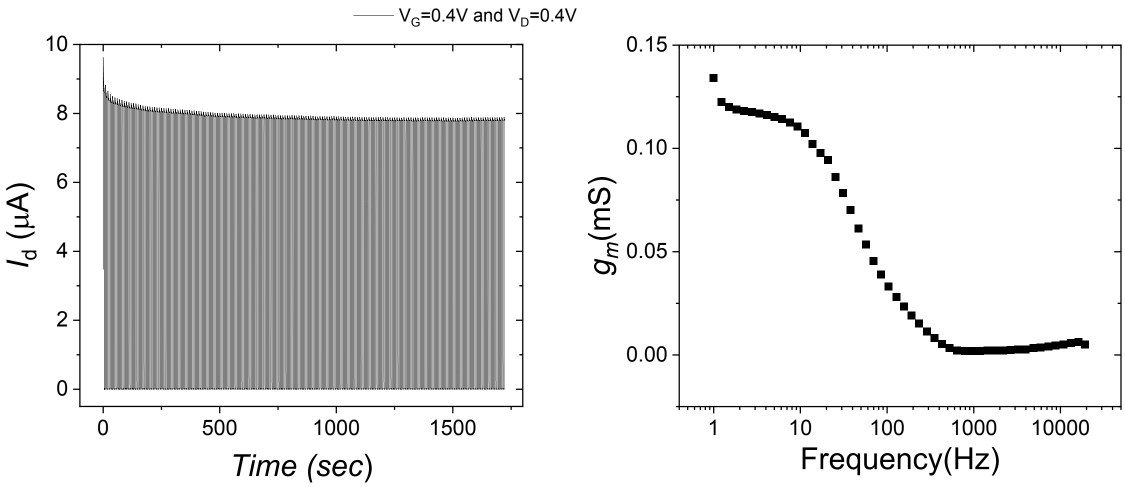

Figure S46. a) Transfer and transconductance curve, b) output curve, c) pulsed OECT stability and d) time dependent transconductance of a **p(g$_7$NC$_4$N)** containing OECT.

4.3.3. p(g$_7$NC$_6$N)

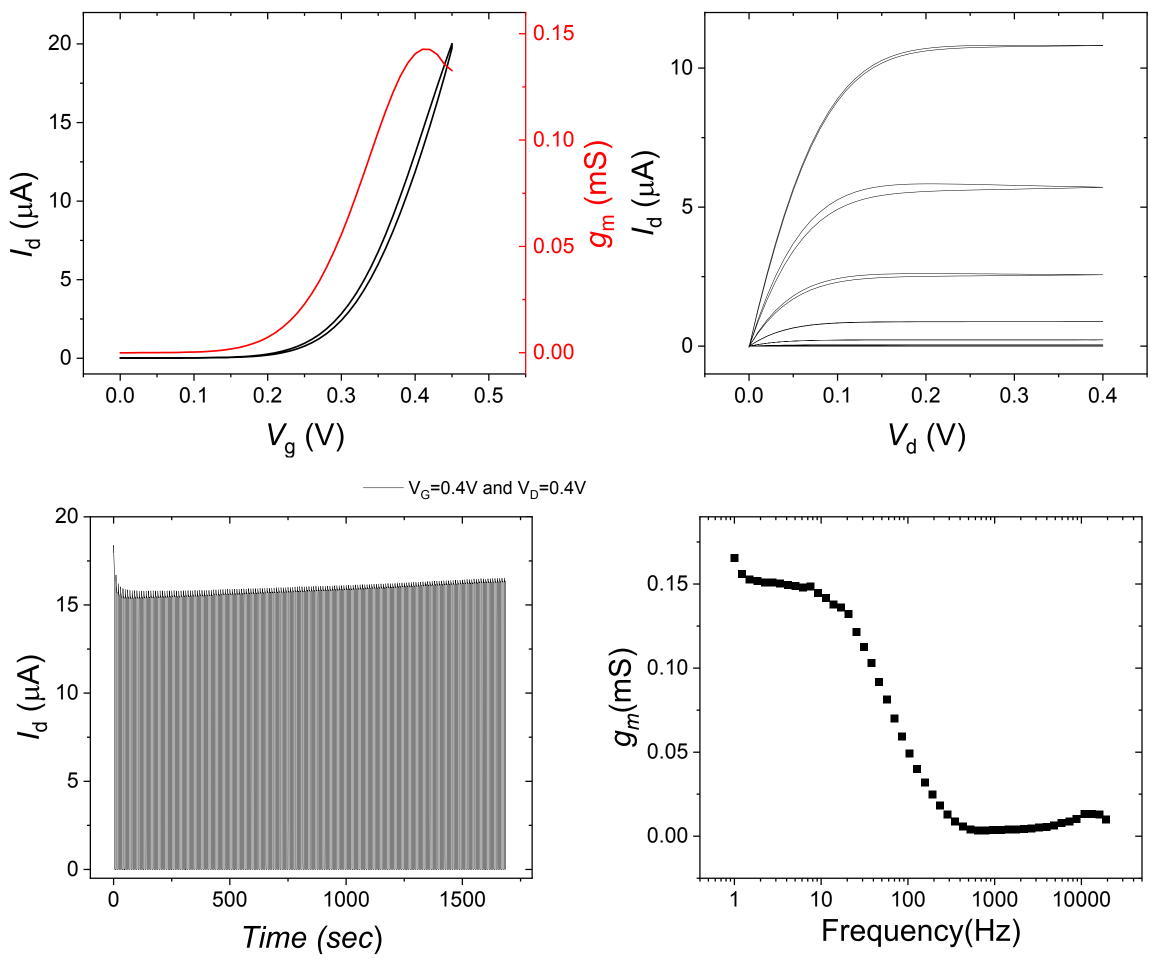

Figure S47. a) Transfer and transconductance curve, b) output curve, c) pulsed OECT stability and d) time dependent transconductance of a **p(g$_7$NC$_6$N)** containing OECT.

4.3.4. p(g_7NC_8N)

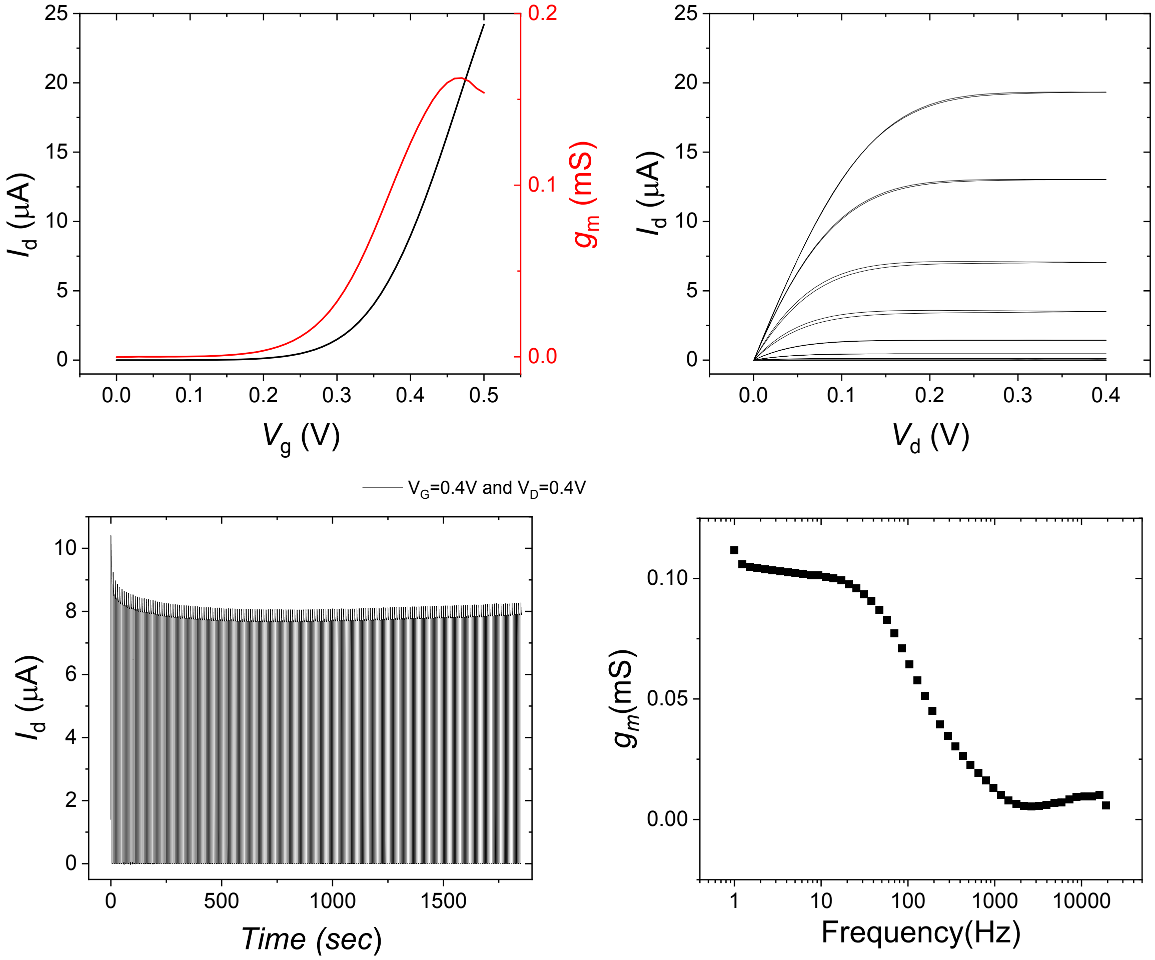

Figure S48. a) Transfer and transconductance curve, b) output curve, c) pulsed OECT stability and d) time dependent transconductance of a **p(g_7NC_8N)** containing OECT.

4.3.5. p(g_7NC_{10}N)

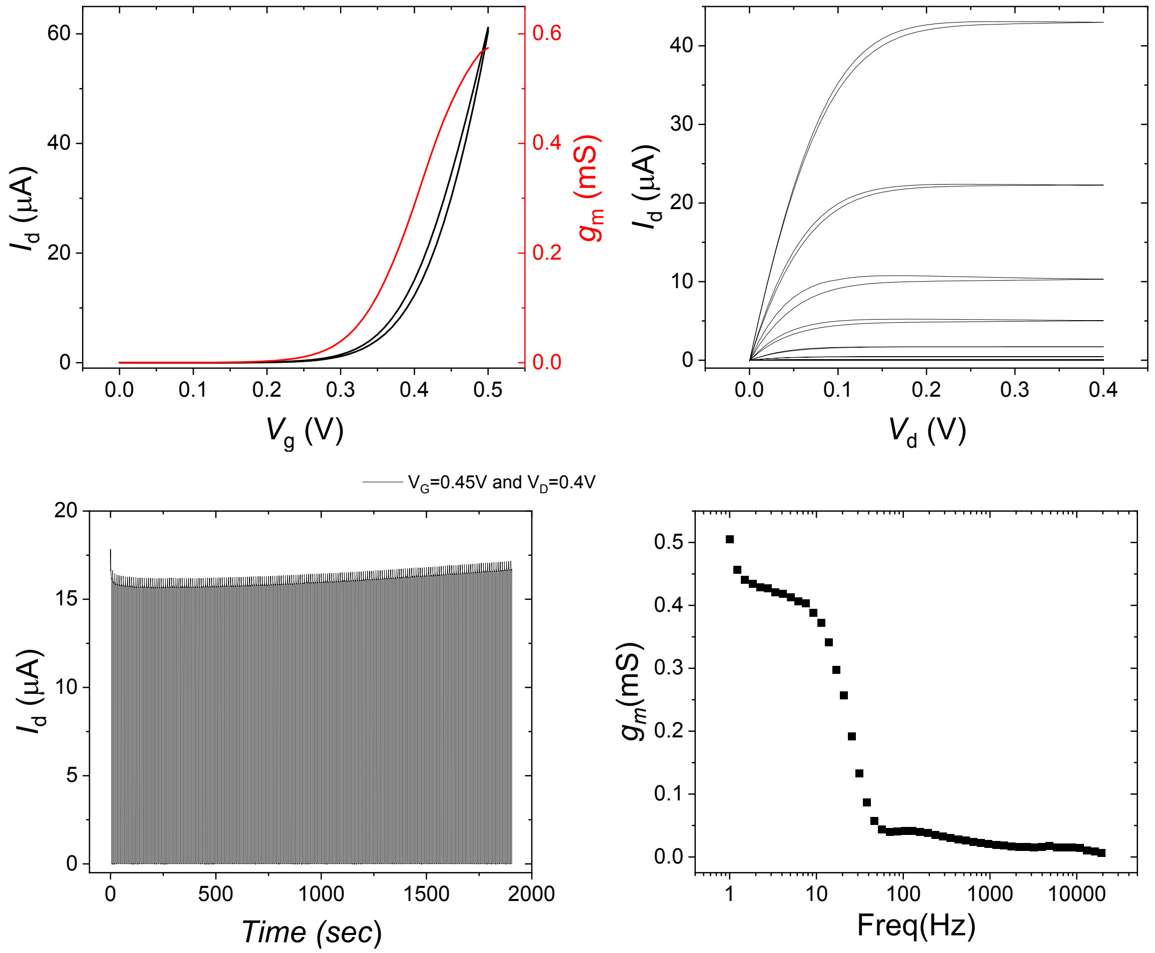

Figure S49. a) Transfer and transconductance curve, b) output curve, c) pulsed OECT stability and d) time dependent transconductance of a **p(g_7NC_{10}N)** containing OECT.

4.3.6. p($g_7NC_{16}N$)

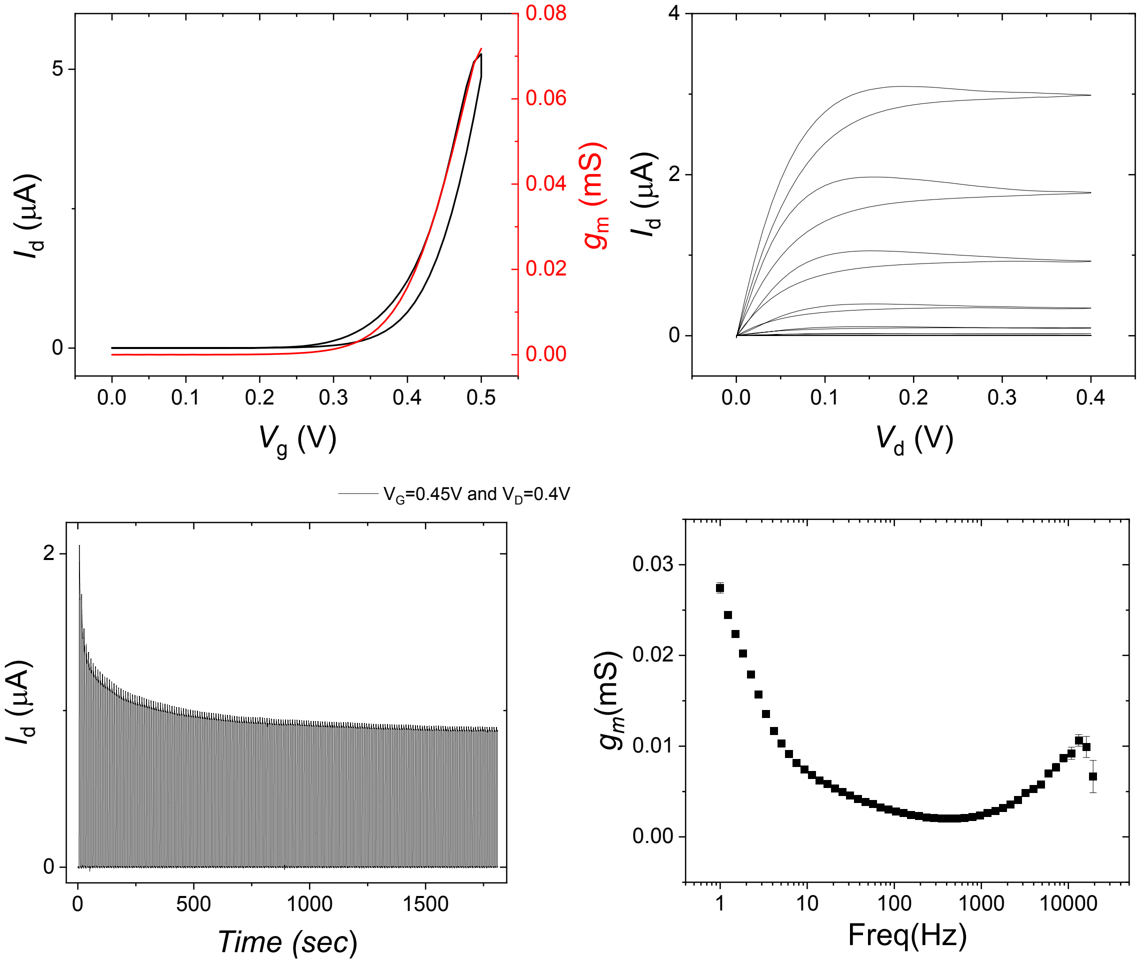

Figure S50. a) Transfer and transconductance curve, b) output curve, c) pulsed OECT stability and d) time dependent transconductance of a **p($g_7NC_{16}N$)** containing OECT.

5. NEAT GRAZING-INCIDENCE WIDE-ANGLE X-RAY SCATTERING DATA

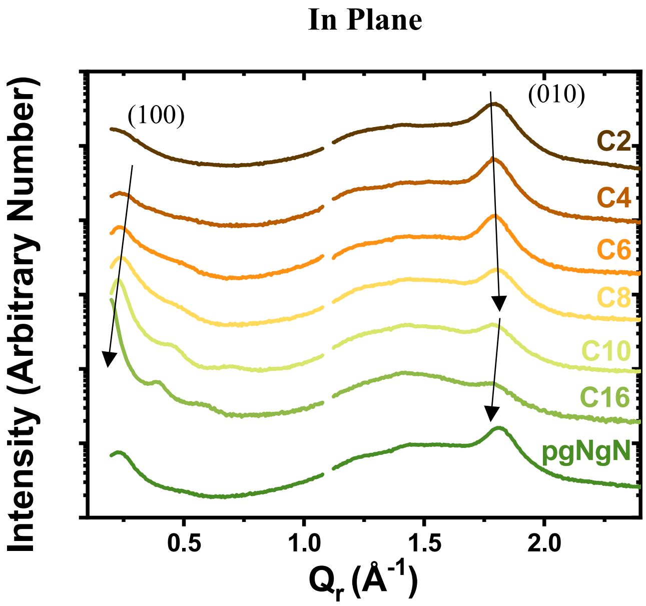

Figure S51. One-dimensional in plane GIWAXS profiles of **p(g_7NC_nN)** polymer neat films.

Table S2. In plane solid state packing parameters for the polymer series.

In plane	$q_{(100)}$ (Å^{-1})	$d_{(100)}$ (Å)	$q_{(010)}$ (Å^{-1})	$d_{(010)}$ (Å)	$L_{C,(010)}$ (Å)
(C2)	-	-	1.79	3.50	38.7
(C4)	0.234	26.9	1.80	3.50	48.1
(C6)	0.226	27.8	1.80	3.50	49.2
(C8)	0.237	26.5	1.81	3.47	38.5
(C10)	0.227	27.7	1.80	3.49	40.4
(C12)[7]	0.222	28.3	1.67	3.76	42.8
(C16)	0.396*	31.7	1.78	3.52	27.8

*Note: Used (200) peak for the polymer.

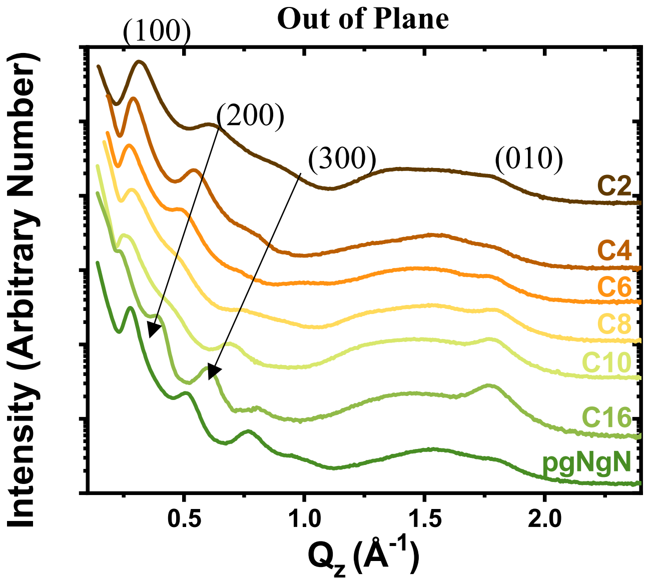

Figure S52. One-dimensional out of plane GIWAXS profiles of **p(g$_7$NC$_n$N)** series neat films.

Table S3. Out of plane solid state packing parameters for the polymer series.

Out of plane	$q_{(200)}$ (Å^{-1})	$d_{(100)}$ (Å)	$q_{(010)}$ (Å^{-1})	$d_{(010)}$ (Å)	$L_{C,(010)}$ (Å)
(C2)	0.611	20.6	-	-	-
(C4)	0.538	23.3	1.77	3.55	27.4
(C6)	0.483	26.0	1.78	3.52	44.9
(C8)	0.425	29.5	1.80	3.49	45.9
(C10)	0.408	30.8	1.79	3.52	40.3
(C12)[7]	-	-	1.67	3.77	47.7
(C16)	0.390	32.2	1.78	3.53	34.8

6. STM imaging

Scanning tunneling microscopy (STM) measurements were conducted on the best performing OECT polymer of the series, **p(g$_7$NC$_{10}$N)**, which was deposited in vacuum at room temperature onto an atomically clean and flat Au(111) surface by electrospray deposition (ESD).[8–11] High-resolution images, as shown in **Figure S53,** allow one to distinguish the backbone and the side chains of individual polymers and thus to sequence the molecules by visual inspection. The majority of the couplings between successive repeat units is as expected: repeat units with C_{10} alkyl side chains (blue in **Figure S53**) are followed by repeat units with g_7 ethylene glycol side chains (green in **Figure S53**) and *vice versa*.

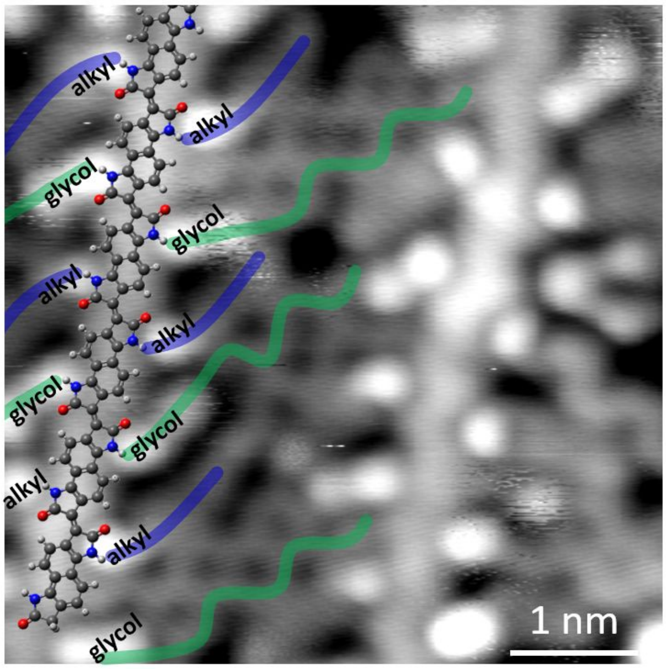

Figure S53. High-resolution STM image of **p(g$_7$NC$_{10}$N)** polymers vacuum deposited on Au(111) by means of ESD. On the left, a scaled and geometry-optimized molecular model (MMFF94 force field in the Avogadro molecular editor) of the polymer backbone is superposed on the image. Blue and green lines indicate the C_{10} alkyl and the g_7 ethylene glycol side chains, respectively. Image size 5 × 5 nm^2, bias voltage (applied to the sample) V = 141 mV, tunneling current I = 110 pA, acquisition temperature T = −196 ˚C.

7. THERMOELECTRIC MEASUREMENTS

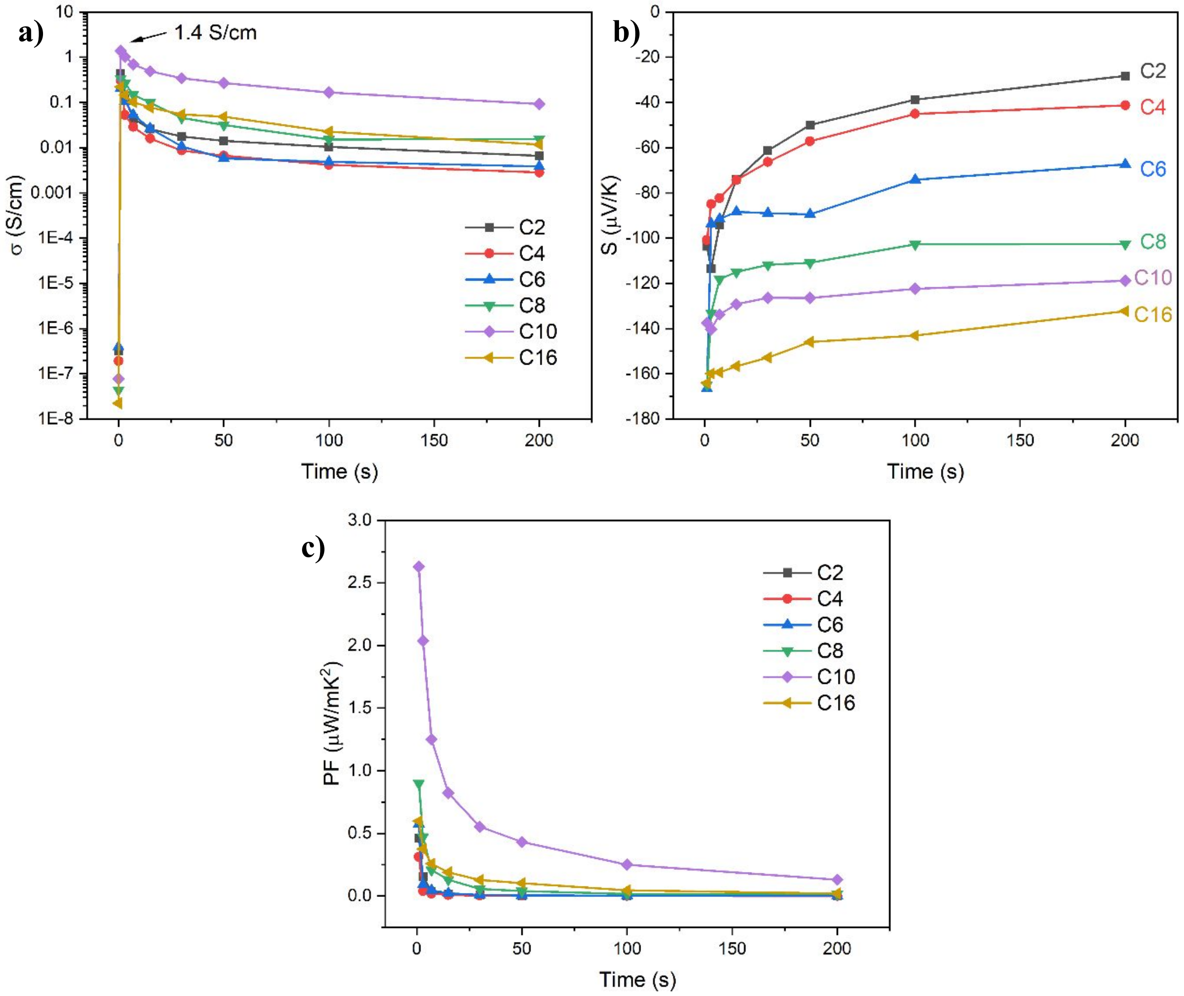

Figure S54. a) Electrical conductivity, b) Seebeck coefficients and c) power factor values recorded for the **p(g$_7$NC$_n$N)** series as a function of time after vapor doping with **TDAE**.

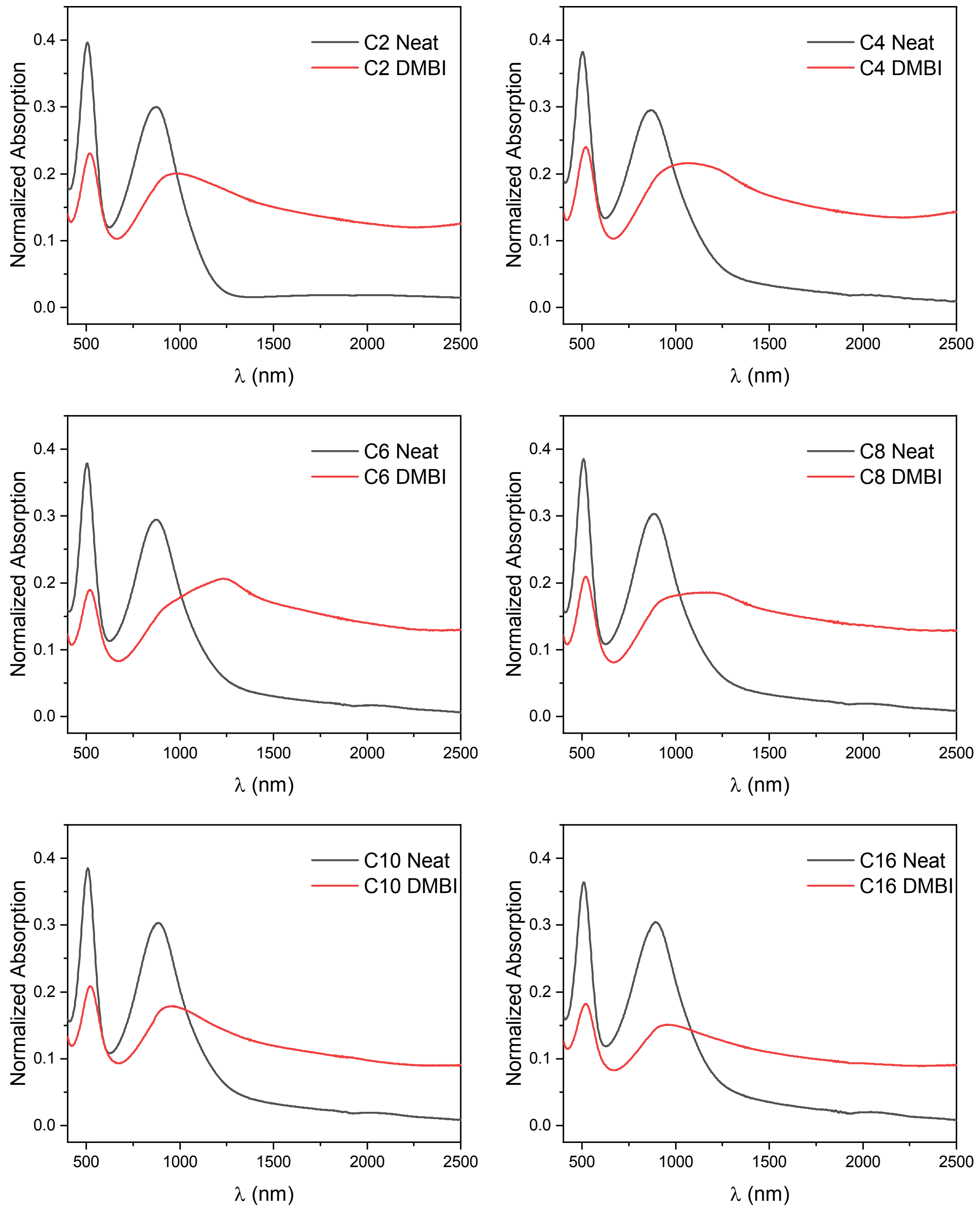

Figure S55. UV–vis spectrum of neat as cast samples and films doped with N-DMBI for the entire **p(g$_7$NC$_n$N)** series.

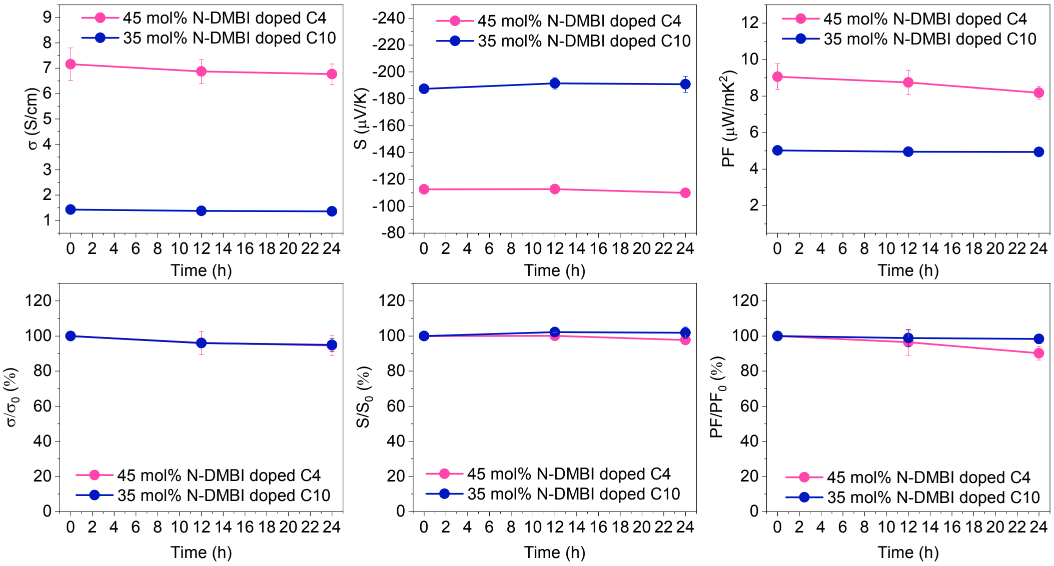

Figure S56. Seebeck coefficient (*S*), electrical conductivity (*σ*) and power factor (*PF*) stability, true values (top) and as a percentage (bottom), of N-DMBI doped (C4) and (C10) polymers, over a period of 24 hours held at 25 °C.

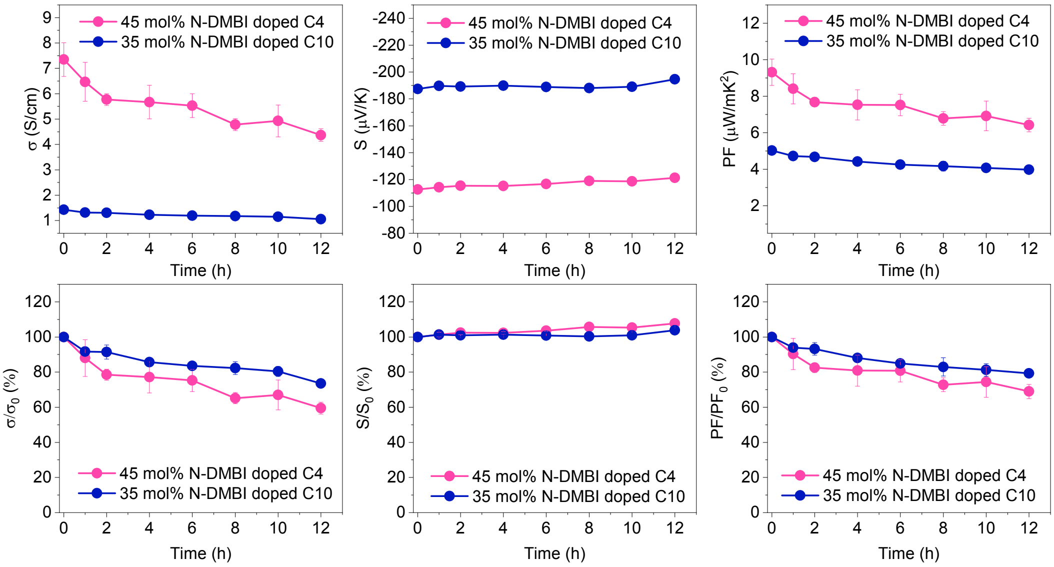

Figure S57. Seebeck coefficient (*S*), electrical conductivity (*σ*) and power factor (*PF*) stability, true values (top) and as a percentage (bottom), of N-DMBI doped (C4) and (C10) polymers, over a period of 12 hours held at 100 °C.

8. DOPED GRAZING-INCIDENCE WIDE-ANGLE X-RAY SCATTERING DATA

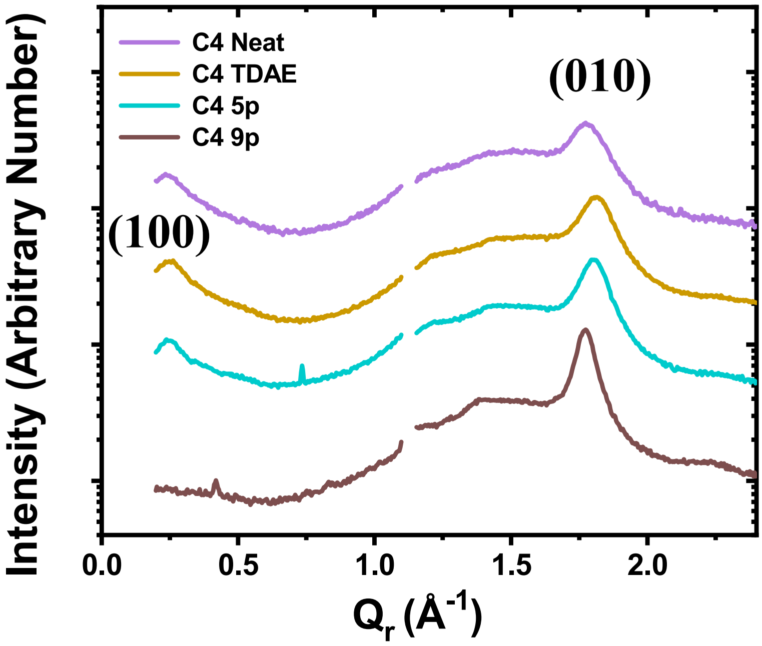

Figure S58. One-dimensional in plane GIWAXS profiles of **p(g$_7$NC$_4$N)** polymer films, neat, vapor doped with TDAE, and chemically doped with 5 and 9 wt% N-DMBI.

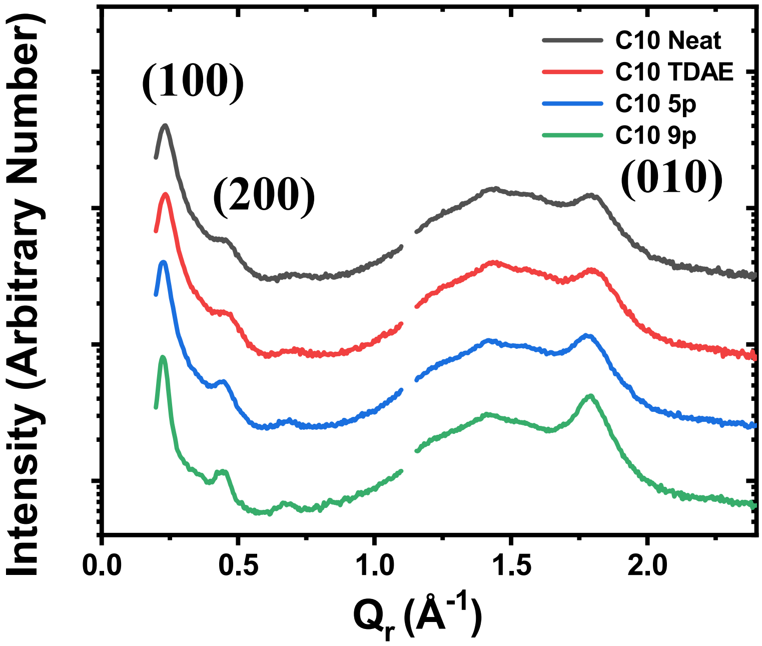

Figure S59. One-dimensional in plane GIWAXS profiles of **p(g$_7$NC$_{10}$N)** polymer films, neat, vapor doped with TDAE, and chemically doped with 5 and 9 wt% N-DMBI.

Table S4. In plane solid state packing parameters for neat and doped **p(g$_7$NC$_4$N)** and **p(g$_7$NC$_{10}$N)** films.

Dopant	$d_{(100)}$ (Å)	$L_{C,(100)}$ (Å)	$d_{(010)}$ (Å)	$L_{C,(010)}$ (Å)
C4 Neat	28.3	21.3	3.52	46.6
C4 TDBA	26.6	22.0	3.46	48.0
C4 5%	26.2	28.0	3.48	56.2
C4 9%	-	-	3.54	80.1
C10 Neat	27.1	78.2	3.49	36.9
C10 TDBA	27.0	84.3	3.47	35.2
C10 5%	27.9	98.2	3.51	44.0
C10 9%	27.9	147	3.50	46.6

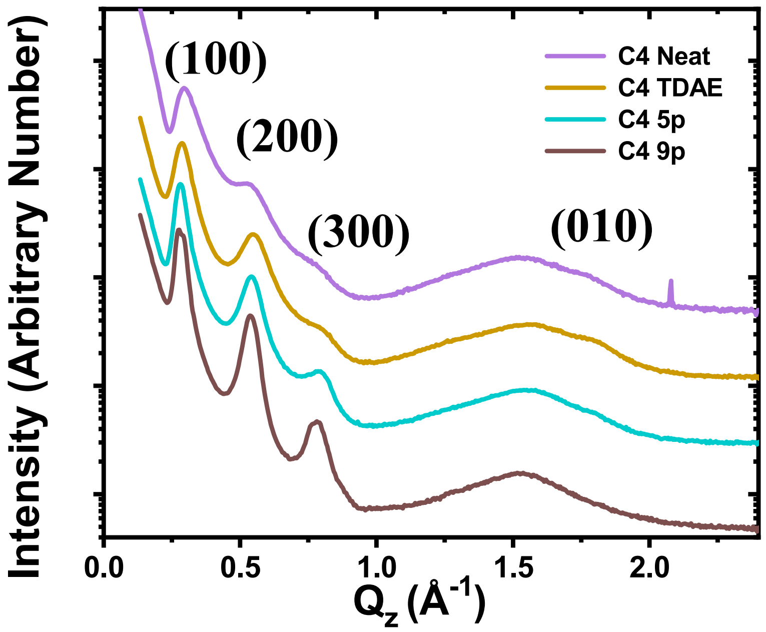

Figure S60. One-dimensional out of plane GIWAXS profiles of **p(g$_7$NC$_4$N)** polymer films, neat, vapor doped with TDAE, and chemically doped with 5 and 9 wt% N-DMBI.

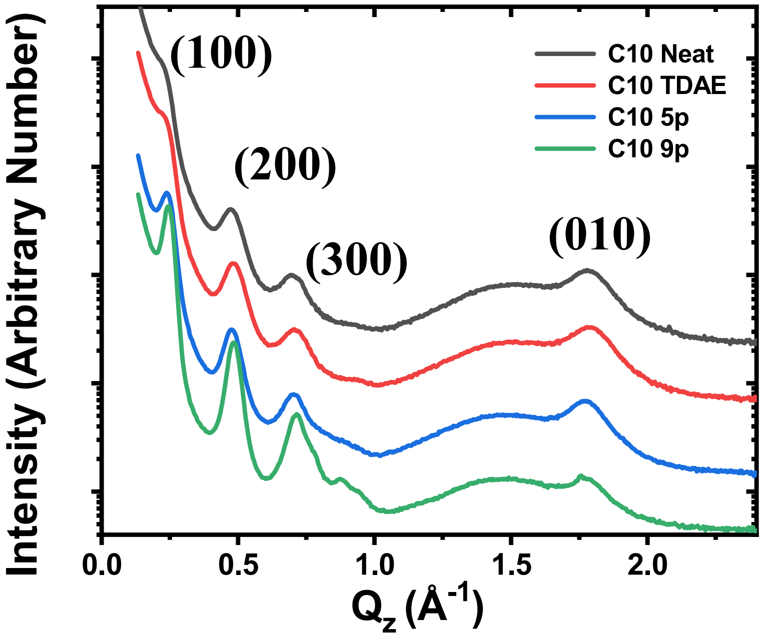

Figure S61. One-dimensional out of plane GIWAXS profiles of **p(g$_7$NC$_{10}$N)** polymer films, neat, vapor doped with TDAE, and chemically doped with 5 and 9 wt% N-DMBI.

Table S5. Out of plane solid state packing parameters for neat and doped **p(g$_7$NC$_4$N)** and **p(g$_7$NC$_{10}$N)** films.

Dopant	$d_{(100)}$ (Å)*	$L_{C,(100)}$ (Å)	$d_{(100)}$ (Å)**	$L_{C,(100)}$ (Å)	$d_{(010)}$ (Å)	$L_{C,(010)}$ (Å)
C4 Neat	24.0	66.5	-	-	3.51	24.0
C4 TDBA	23.0	104	23.9	325	3.46	23.0
C4 5%	23.3	140	23.9	223	3.48	23.3
C4 9%	23.3	223	24.1	289	-	23.3
C10 Neat	26.3	159	26.8	188	3.51	26.3
C10 TDBA	25.9	157	26.5	179	3.50	25.9
C10 5%	26.3	187	26.7	166	3.53	26.3
C10 9%	26.0	243	26.4	270	3.52	26.0

*Listed are $d_{(100)}$ calculated from (200).
**Listed are $d_{(100)}$ calculated from (300).

9. REFERENCES

(1) Jiang, Z. GIXSGUI : A MATLAB Toolbox for Grazing-Incidence X-Ray Scattering Data Visualization and Reduction, and Indexing of Buried Three-Dimensional Periodic Nanostructured Films. *J. Appl. Crystallogr.* **2015**, *48* (3), 917–926.

(2) Parr, Z. S.; Halaksa, R.; Finn, P. A.; Rashid, R. B.; Kovalenko, A.; Weiter, M.; Rivnay, J.; Krajčovič, J.; Nielsen, C. B. Glycolated Thiophene-Tetrafluorophenylene Copolymers for Bioelectronic Applications: Synthesis by Direct Heteroarylation Polymerisation. *Chempluschem* **2019**, *84* (9), 1384–1390.

(3) Tarabella, G.; Mahvash Mohammadi, F.; Coppedè, N.; Barbero, F.; Iannotta, S.; Santato, C.; Cicoira, F. New Opportunities for Organic Electronics and Bioelectronics: Ions in Action. *Chem. Sci.* **2013**, *4* (4), 1395–1409.

(4) Lu, Y.; Yu, Z.-D.; Liu, Y.; Ding, Y.-F.; Yang, C.-Y.; Yao, Z.-F.; Wang, Z.-Y.; You, H.-Y.; Cheng, X.-F.; Tang, B.; Wang, J.-Y.; Pei, J. The Critical Role of Dopant Cations in Electrical Conductivity and Thermoelectric Performance of N-Doped Polymers. *J. Am. Chem. Soc.* **2020**, *142* (36), 15340–15348.

(5) Yang, C.-Y.; Ding, Y.-F.; Huang, D.; Wang, J.; Yao, Z.-F.; Huang, C.-X.; Lu, Y.; Un, H.-I.; Zhuang, F.-D.; Dou, J.-H.; Di, C.; Zhu, D.; Wang, J.-Y.; Lei, T.; Pei, J. A Thermally Activated and Highly Miscible Dopant for N-Type Organic Thermoelectrics. *Nat. Commun.* **2020**, *11* (1), 3292.

(6) Wang, S.; Sun, H.; Ail, U.; Vagin, M.; Persson, P. O. Å. Å.; Andreasen, J. W.; Thiel, W.; Berggren, M.; Crispin, X.; Fazzi, D.; Fabiano, S. Thermoelectric Properties of Solution-Processed n-Doped Ladder-Type Conducting Polymers. *Adv. Mater.* **2016**, *28* (48), 10764–10771.

(7) Chen, X.; Marks, A.; Paulsen, B. D.; Wu, R.; Rashid, R. B.; Chen, H.; Alsufyani, M.; Rivnay, J.; McCulloch, I. N -Type Rigid Semiconducting Polymers Bearing Oligo(Ethylene Glycol) Side Chains for High-Performance Organic Electrochemical Transistors. *Angew. Chemie Int. Ed.* **2021**, *60* (17), 9368–9373.

(8) Hallani, R. K.; Paulsen, B. D.; Petty, A. J.; Sheelamanthula, R.; Moser, M.; Thorley, K. J.; Sohn, W.; Rashid, R. B.; Savva, A.; Moro, S.; Parker, J. P.; Drury, O.; Alsufyani, M.; Neophytou, M.; Kosco, J.; Inal, S.; Costantini, G.; Rivnay, J.; McCulloch, I. Regiochemistry-Driven Organic Electrochemical Transistor Performance Enhancement in Ethylene Glycol-Functionalized Polythiophenes. *J. Am. Chem. Soc.* **2021**, *143* (29), 11007–11018.

(9) Ponder Jr, J. F.; Chen, H.; Luci, A. M. T.; Moro, S.; Turano, M.; Hobson, A. L.; Collier, G. S.; Perdigão, L. M. A.; Moser, M.; Zhang, W.; Costantini, G.; Reynolds, J. R.; McCulloch, I. Low-Defect, High Molecular Weight Indacenodithiophene (IDT) Polymers Via a C–H Activation: Evaluation of a Simpler and Greener Approach to Organic Electronic Materials. *ACS Mater. Lett.* **2021**, *3* (10), 1503–1512.

(10) Xiao, M.; Kang, B.; Lee, S. B.; Perdigão, L. M. A.; Luci, A.; Warr, D. A.; Senanayak, S. P.; Nikolka, M.; Statz, M.; Wu, Y.; Sadhanala, A.; Schott, S.; Carey, R.; Wang, Q.; Lee, M.; Kim, C.; Onwubiko, A.; Jellett, C.; Liao, H.; Yue, W.; Cho, K.; Costantini, G.; McCulloch, I.; Sirringhaus, H. Anisotropy of Charge Transport in a Uniaxially Aligned Fused Electron-Deficient Polymer Processed by Solution Shear Coating. *Adv. Mater.* **2020**, *32* (23), 2000063.

(11) Warr, D. A.; Perdigão, L. M. A.; Pinfold, H.; Blohm, J.; Stringer, D.; Leventis, A.; Bronstein, H.; Troisi, A.; Costantini, G. Sequencing Conjugated Polymers by Eye. *Sci. Adv.* **2018**, *4* (6).
